# Chaotic Regulation of Exploration and Exploitation in Bio-Inspired Swarm Intelligence for Combinatorial Optimization

**DOI:** 10.3390/biomimetics11080540

**Published:** 2026-08-03

**Authors:** Felipe Cisternas-Caneo, Broderick Crawford, Jorge Mendoza, José M. Lanza-Gutiérrez, José Barrera-García, Ricardo Soto

**Affiliations:** 1 Escuela de Ingeniería Informática, Pontificia Universidad Católica de Valparaíso, Avenida Brasil 2241, Valparaíso 2362807, Chile; jose.barrera@pucv.cl (J.B.-G.); ricardo.soto@pucv.cl (R.S.); 2 Departamento de Ciencias de la Computación, Escuela Politécnica Superior, Universidad de Alcalá, 28805 Alcalá de Henares, Spain; jm.lanza@uah.es; 3 Escuela de Ingeniería Eléctrica, Pontificia Universidad Católica de Valparaíso, Valparaíso 2362804, Chile; 4 Escuela de Negocios y Economía, Pontificia Universidad Católica de Valparaíso, Amunátegui 1838, Viña del Mar 2580129, Chile

**Keywords:** swarm intelligence, collective behavior, chaotic maps, binarization schemes, landscape adaptability

## Abstract

The transition from continuous swarm intelligence algorithms to discrete combinatorial domains remains a critical challenge in bio-inspired computing. Traditional binarization techniques frequently induce premature convergence in highly constrained landscapes. This paper presents a chaotic discretization framework that replaces the classical behavior of the two-step binarization technique to regulate the balance between exploration and exploitation. The proposal systematically integrates three leading continuous metaheuristics in the literature, with twenty-four binarization configurations, across three distinct NP-hard problem archetypes: capacity-constrained (0–1 Knapsack), sparse (Set Covering), and mathematically degenerate flat landscapes (Unicost Set Covering). Nonparametric statistical tests confirm that chaotic discretization acts as a powerful regulator in the landscape (*p* < 0.05). Empirical evidence shows that the highest-performing chaotic mapping is heavily influenced by the specific landscape morphology evaluated: the 0–1 Knapsack Problem is statistically optimized by the Circle map under standard rules; the Set Covering Problem achieves optimal median performance with the Tent map under elitist formulations, although severe matrix constraints ultimately force statistical ties; and the Unicost Set Covering Problem utilizes the nonlinear sequences of the sinusoidal map under complementary operators to break convergence stagnation.

## 1. Introduction

Population-based metaheuristics inspired by swarm intelligence operating within continuous search spaces update their decision vectors through continuous differential, velocity, or oscillatory trigonometric vector operations [1]. While these methodologies model emergent collective behaviors efficiently in continuous multi-dimensional functions, a structural mismatch occurs when they are applied to binary optimization problems, mandating the integration of two-step binarization techniques to map continuous coordinate displacements into the vertices of a discrete hypercube ${\left\{0,1\right\}}^{n}$ [2].

In canonical binarization procedures, state transitions are resolved by processing the continuous velocity vectors through a transfer function to yield a probability matrix, which is subsequently evaluated against a threshold generated by a uniform pseudo-random number generator (PRNG) where rand∼U(0,1) [3]. Despite its computational simplicity, uniform stochastic thresholding can constrain search-trajectory efficiency due to distribution saturation and the lack of intrinsic temporal correlation in purely random sequences, which can frequently trap the swarm in premature local optima. To address this structural limitation of uniform randomness, a compelling alternative is to integrate deterministic chaotic sequences into the conditional thresholding rules, leveraging properties such as ergodicity, non-periodicity, and high sensitivity to initial conditions [4]. Nevertheless, the physical impact of these chaotic dynamics on the macroscopic exploration and localized exploitation trade-off does not act in isolation; rather, it is strictly contingent on the constraints and morphology of the search space.

To comprehensively characterize the performance limits and topological adaptability of this discretization interface, this research evaluates algorithmic performance across three distinct combinatorial optimization paradigms, establishing a tripartite landscape-validation framework based on search-space morphology. First, capacity-constrained landscapes, represented by the 0–1 Knapsack Problem (KP), exhibit strict capacity limits and benefit–weight correlations that force the swarm to maintain granular accuracy near the feasibility limits [5]. Secondly, sparse coverage landscapes, represented by the Set Cover Problem (SCP), are dominated by highly sparse constraint matrices with densities between 2% and 20%, where cost minimization demands strong selection pressure to eliminate redundant variable activations while ensuring gross row coverage [6]. Third, symmetrical flat landscapes, represented by the Unicost Set Covering Problem (USCP), introduce uniform column weights ($c_{j}=1$) that fundamentally eliminate the informative gradient of the landscape, isolating extensive flat plateaus characterized by severe mathematical degeneracy where distinct binary solutions yield identical fitness costs [7].

To ensure that the characterization of this chaotic binarization framework is free from biases of the underlying algorithm, this binarization process is systematically integrated into three metaheuristics that feature fundamentally different continuous search mechanisms: the Grey Wolf Optimizer (GWO) [8], the Sine-Cosine Algorithm (SCA) [9], and the Whale Optimization Algorithm (WOA) [10]. In keeping with the interdisciplinary nature of bio-inspired design, the explicit contribution of this work is to establish a biomimetic statement on how artificial swarms adapt their collective intelligence to distinct morphologies, replacing standard uniform stochasticity with chaotic sequences to optimize phase transitions. This proposal uses a dual analytical validation pipeline that tracks macroscopic accuracy optimization via relative percent deviation (RPD), in parallel with microscopic recording via convergence and, in real time, population momentum using the Hussain distance-based diversity metric [11]. Through this microspatial approach, the validation methodology demonstrates the ineffectiveness of conventional percent phase metrics, which are blind to high-dimensional discretization constraints due to rapid spatial compression within constrained hypercubes [12].

In synthesis, the fundamental contributions of this research are structured as follows:

1. Characterization of Landscape Degradation and Chaotic Alleviation: We experimentally demonstrate how the structural characteristics of distinct search-space topologies significantly degrade baseline metaheuristic performance. Concurrently, we prove that the injection of chaotic binarizations provides a valuable empirical mechanism to combat convergence stagnation, while cautiously acknowledging the operational boundaries enforced by the No Free Lunch theorem.
2. Identification of Analytical Blindness in Traditional Phase Metrics: We identify and analyze a limitation in conventional macro-spatial exploration–exploitation percentage metrics within discrete frameworks. We demonstrate that these widespread indicators suffer from analytical blindness and a complete loss of operational resolution when applied to high-dimensional spaces, where the underlying algorithms undergo rapid spatial compression and accelerated convergence, highlighting their interpretive limits in scaled combinatorial domains.
3. Algorithmic Susceptibility Mapping: Through rigorous non-parametric multi-comparison validation, we map how chaotic binarization acts as a structural regulator that alters performance paths based on the base continuous architecture, functioning as an essential lifesaver against stagnation for trapping-prone solvers (GWO) while operating as a precision refiner for naturally resilient wave-based structures (SCA and WOA).

The remainder of this paper is organized as follows. Section 2 reviews the mathematical foundations of continuous swarm intelligence and outlines the baseline metaheuristics. Section 3 describes the chaotic binarization framework and integration pipeline. Section 4 provides the problem characterization and analyzes the distinct landscape topologies under study. Section 5 defines the complete experimental setup alongside the metrics and statistical validation protocol. Section 6 presents the comparative results and discussion structured across the three discrete morphologies. Finally, Section 7 outlines the primary conclusions and suggests future research directions.

## 2. Theoretical Foundations of Continuous Swarm Intelligence

Systematic evaluation of a discrete optimization framework requires a comprehensive characterization of baseline continuous algorithms to identify the operational dynamics that drive collective intelligence. Bioinspired population-based metaheuristics balance exploration and exploitation through mathematical models that simulate natural self-organization, social hierarchies, or predatory coordination mechanisms. To evaluate the generalization capability and robustness of the chaotic binarization schemes, three continuous swarm intelligence metaheuristics were selected: Sine Cosine Algorithm (SCA) [9], Grey Wolf Optimizer (GWO) [8], and Whale Optimization Algorithm (WOA) [10]. This selection is strategically designed to stress-test the discretization framework across a diverse spectrum of continuous search dynamics. To demonstrate that the chaotic integration is an effective regulator, it must be validated on base algorithms that manage the exploration–exploitation trade-off using fundamentally different mathematical paradigms:

- SCA utilizes trigonometric oscillations to create a highly dynamic transition between exploration and exploitation. Its single-leader guidance makes it particularly sensitive to the thresholding behavior of the binarization interface. SCA has proven to be an algorithm of great interest to researchers with applications for the optimization of construction programs [13], the diagnosis of corrosion of hydroelectric grounding networks [14], the improved identification of digital IIR filter systems [15], the detection of intrusions in the cloud [16], or improving multimodal biometric authentication [17].
- GWO employs a hierarchical leadership mechanism guided by the three best agents (α, β, and δ). This multi-leader approach creates a complex convergence pressure, serving as a rigorous benchmark for the chaotic maps’ ability to regulate bit-flipping in multi-directional search spaces. It is well known that GWO has aroused great interest in the scientific community in recent times [1]. This interest is reflected in applications such as the optimization of task allocation to construction teams [18], the non-invasive prediction of breast cancer biomarkers [19], UAV trajectory planning [20], robust single-image dehazing [21], and the resource leveling problem in construction projects [22].
- WOA integrates a probabilistic dual-mechanism during its exploitation phase, switching between shrinking circles and logarithmic spirals. This structural stochasticity adds a layer of continuous-displacement complexity, testing whether chaotic binarization can maintain stability amid high-variance continuous updates. WOA is a metaheuristic that, like GWO, is gaining interest among researchers [23]. WOA has been used for applications such as aircraft assembly scheduling [24], clinical classification of lung cancer from chest computed tomography images [25], optimization of the design of a remote center compliance device for pin-in-hole assembly [26], distributed generation allocation in distribution networks [27], and flexible shop scheduling problems with machinery deterioration effects [28].

By validating our framework across these distinct mathematical architectures, we ensure that the performance improvements are attributable to the chaotic discretization regulation rather than the specific bias of any single metaheuristic.

### 2.1. Grey Wolf Optimizer

Conceptually, the Grey Wolf Optimizer (GWO) is inspired by the strict social hierarchy and collective hunting maneuvers observed in grey wolf packs in nature [8]. From a biomimetic perspective, this algorithm models decentralized information transfer and swarm coordination through a social structure where the search trajectories of subordinate agents (ω) are simultaneously steered by an elite trio representing the three best candidate solutions localized in the phase space: alpha (α), beta (β), and delta (δ). Scientifically, the selection of GWO is justified by its strong relevance and widespread acceptance in the optimization literature [29,30,31], making it one of the most thoroughly validated metaheuristics for complex engineering design, owing to its high convergence speed and minimal parameter-tuning overhead. This continuous multi-leader architecture exerts a rigid constriction pressure on the swarm, forcing agents to rapidly concentrate their step vectors within a narrow radius, thereby establishing a directional search logic with significant local intensification capability.

Mathematically, the encirclement and tracking of the target prey are modeled by computing independent distance vectors relative to the three population leaders:

(1) $$D_{\alpha }=\left|C_{1}X_{\alpha ,j}^{t}-X_{i,j}^{t}\right|,\;X_{1}=X_{\alpha ,j}^{t}-A_{1}D_{\alpha }$$

(2) $$D_{\beta }=\left|C_{2}X_{\beta ,j}^{t}-X_{i,j}^{t}\right|,\;X_{2}=X_{\beta ,j}^{t}-A_{2}D_{\beta }$$

(3) $$D_{\delta }=\left|C_{3}X_{\delta ,j}^{t}-X_{i,j}^{t}\right|,\;X_{3}=X_{\delta ,j}^{t}-A_{3}D_{\delta }$$

where *t* denotes the current iteration, $X_{i,j}^{t}$ represents the position of the *i*-th individual in the j−th dimension, and $X_{\alpha ,j}^{t}$, $X_{\beta ,j}^{t}$, and $X_{\delta ,j}^{t}$ define the coordinates of the three leaders. The coefficients are formulated as $A_{n}=2ar_{1}-a$ and $C_{n}=2r_{2}$, where the control parameter *a* decreases linearly from 2 to 0 over the generations to simulate predatory constriction, while $r_{1}$ and $r_{2}$ are independent random numbers drawn from a uniform distribution in the interval [0,1]. The final coordinate update for the *j*-th dimension of the *i*-th agent is determined by computing the mathematical average of these three directional forces:

(4) $$X_{i,j}^{t+1}=\frac{X_{1}+X_{2}+X_{3}}{3}$$

### 2.2. Sine Cosine Algorithm

In contrast to the multi-point hierarchical guidance of GWO, the Sine Cosine Algorithm (SCA) is conceptually inspired by geometric principles and mathematical wave fluctuations to direct search agents [9]. The biomimetic argument supporting its integration within a complex systems framework lies in its capacity to model radial oscillation patterns observed in various natural kinetic phenomena, such as periodic biological foraging behaviors or nonlinear fluid turbulence. Scientifically, the selection of SCA reflects its status as a high-performance benchmark in the literature [32], widely valued for its exceptional capacity for macroscopic exploration, derived from the alternating use of trigonometric functions. This cyclic motion pattern introduces a continuous wave-like behavior that prevents the swarm from prematurely collapsing into single points of attraction, establishing a fluctuating search dynamic that serves as an ideal baseline for evaluating how continuous periodic operators perform within discrete search boundaries.

The continuous displacement of search agent coordinates in SCA is executed relative to the best global destination ($X_{best}$) through the stochastic switching of sine and cosine functions:

(5) $$X_{i,j}^{t+1}=\left\{\begin{array}{cc} X_{i,j}^{t}+r_{1}\sin\left(r_{2}\right)\cdot \left|r_{3}X_{best,j}^{t}-X_{i,j}^{t}\right| & \mathrm{if}\;r_{4}<0.5\\ X_{i,j}^{t}+r_{1}\cos\left(r_{2}\right)\cdot \left|r_{3}X_{best,j}^{t}-X_{i,j}^{t}\right| & \mathrm{if}\;r_{4}\geq 0.5 \end{array}\right.$$

Within this formulation, the primary control parameter is $r_{1}$, defined as $r_{1}=a-t\frac{a}{T}$, a deterministic coefficient that decreases linearly from a predefined constant *a* to 0 across generations to regulate the constriction of the search radius. The parameter $r_{2}$ is a uniform random number distributed in the interval [0,2π] that defines the angle of the trigonometric oscillation, whereas $r_{3}$ represents a uniform stochastic weight ∈[0,2] that scales or attenuates the influence of the global best position. Finally, $r_{4}$ is a uniform random number strictly bounded within [0,1] that operates as the probabilistic selector, providing a balanced 50% chance to switch between the sine and cosine displacement.

### 2.3. Whale Optimization Algorithm

The Whale Optimization Algorithm (WOA) is conceptually inspired by the complex cooperative foraging maneuver known as the bubble-net feeding strategy, executed by humpback whales in marine ecosystems [10]. The fundamental biomimetic argument justifying its inclusion within the scope of bio-inspired design is its capacity to model complex continuous helical trajectories, a sophisticated non-linear optimization pattern that accurately represents the spatial kinematics of macro-organisms. In the scientific domain, WOA is heavily accepted by the research community due to its minimal parametric tuning overhead compared to classical evolutionary algorithms, reducing structural complexity while maximizing exploratory flexibility [33,34,35,36]. By incorporating a motion duality based on continuous spirals perturbed by high stochastic volatility, WOA introduces a high-frequency continuous noise profile that rigorously tests the stability of binarization rules across degenerate search dimensions.

The optimization cycle in WOA evaluates search agent positions against a uniform random threshold probability (*p*) bounded within [0,1] to determine the switch between encircling maneuvers and helical updates. When the condition p≥0.5 is satisfied, the agents execute a localized exploitation phase that mimics the concentric spiral of a bubble-net attack around the current leader ($X_{best}$):

(6) $$X_{i,j}^{t+1}=\left|X_{best,j}^{t}-X_{i,j}^{t}\right|e^{bl}\cos\left(2\pi l\right)+X_{best,j}^{t}$$

where the deterministic constant *b* defines the geometric profile and aperture coefficient of the logarithmic spiral, while *l* is a uniform random variable inside the interval [−1,1] that controls the vertical oscillation along the path. Conversely, when p<0.5, the displacement logic is dictated by the magnitude of the adaptive vector coefficient A=2a·rand(0,1)−a, where the factor *a* decreases linearly from 2 to 0. If the condition |A|<1 is met, a shrinking encircling mechanism guided by the leader is executed:

(7) $$X_{i,j}^{t+1}=X_{best,j}^{t}-A\left|CX_{best,j}^{t}-X_{i,j}^{t}\right|$$

If the operational condition switches to |A|≥1, the global exploration phase is triggered, forcing search agents to diverge from the leader and update their continuous steps relative to a randomly selected individual from the population ($X_{random}$):

(8) $$X_{i,j}^{t+1}=X_{random,j}^{t}-A\left|CX_{random,j}^{t}-X_{i,j}^{t}\right|$$

In both encircling equations, the variable C=2·rand(0,1) functions as a uniform stochastic weight ∈[0,2] designed to simulate the non-linear resistance effects of the environment on the continuous step displacement.

In summary, the mathematical formulations of GWO, SCA, and WOA reveal three radically distinct continuous search dynamics, which justifies their systematic comparison within this research. GWO operates on a rigid social hierarchy that compresses the swarm inward using multi-leader attraction vectors, emphasizing high-precision local intensification. Conversely, SCA relies on symmetric, wave-based trigonometric equations that introduce periodic radial fluctuations, maximizing macroscopic space exploration and preventing early spatial collapse. Alternating between these paradigms, WOA implements a highly volatile probabilistic dual-mechanism, driven by logarithmic-spiral trajectories and random-vector divergence, injecting a complex non-linear noise profile into the continuous phase space. Evaluating the proposed chaotic binarization framework against these three contrasting continuous paradigms provides a rigorous validation environment.

## 3. Chaotic Binarization Framework

Adapting swarm intelligence metaheuristics to discrete domains is primarily justified by the No Free Lunch Theorem (NFL) [37]. The NFL theorem states that no optimization algorithm can achieve superior performance across all possible landscape topologies, motivating the adoption of novel optimization approaches. To address this limitation without causing a complete architectural collapse, researchers often modify the internal operating logic of metaheuristics to create native binary variants [3]. However, altering the underlying vector algebra risks disrupting or completely destroying the large-scale collective behaviors that made these search engines effective in their native continuous space. Consequently, this research uses an external continuous-to-discrete interface based on the two-step binarization paradigm [2]. This mechanism serves as a non-invasive topological bridge, preserving the integrity of the continuous update rules at the macro level while introducing a minimal, highly localized modification during threshold validation to satisfy discrete constraints.

### 3.1. The Two-Step Binarization

**Figure 1:**
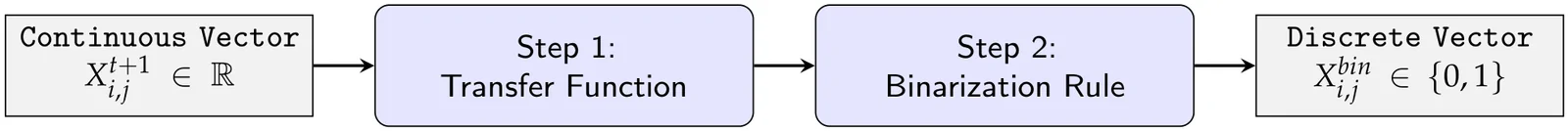
Structural pipeline of the two-step continuous-to-discrete binarization interface.

The standard two-step binarization technique operates as a structural pipeline that processes continuous coordinate displacements and transforms them into discrete binary variables. In the first step, the continuous coordinate variation $X_{i,j}^{t+1}$ computed by the metaheuristic is passed through a bounded transfer function $T(X_{i,j}^{t+1})$, normalizing the real value into a continuous probability interval [0,1]. In the second step, a discrete binarization rule processes this probability output against a selection threshold to establish the final discrete state variable within the hypercube vertex ${\left\{0,1\right\}}^{n}$. This structural transition is illustrated in Figure 1.

Once the continuous coordinate displacement $X_{i,j}^{t+1}$ is computed, it must be normalized into a probability space. Within the discretization literature, this mapping is traditionally achieved through two main taxonomies of transfer functions [2,3]: S-shaped and V-shaped curves, comprising eight classical mathematical formulations designed to alter the state-transition probability profiles differently. To provide a comprehensive structural overview of these mathematical interfaces, the complete taxonomy of classical S-shaped transfer functions is defined as follows:

(9a) $$\begin{array}{c} T_{S1}\left(X_{i,j}^{t+1}\right)=\frac{1}{1+e^{-2X_{i,j}^{t+1}}},\;T_{S2}\left(X_{i,j}^{t+1}\right)=\frac{1}{1+e^{-X_{i,j}^{t+1}}} \end{array}$$

(9b) $$\begin{array}{c} T_{S3}\left(X_{i,j}^{t+1}\right)=\frac{1}{1+e^{\frac{-X_{i,j}^{t+1}}{2}}},\;T_{S4}\left(X_{i,j}^{t+1}\right)=\frac{1}{1+e^{\frac{-X_{i,j}^{t+1}}{3}}} \end{array}$$

Similarly, the mathematical formulations governing the V-shaped transfer function taxonomy are structured as follows:

(10a) $$\begin{array}{c} T_{V1}\left(X_{i,j}^{t+1}\right)=\left|\mathrm{erf}\left(\frac{\sqrt{\pi }}{2}X_{i,j}^{t+1}\right)\right|,\;T_{V2}\left(X_{i,j}^{t+1}\right)=\left|\tanh(X_{i,j}^{t+1})\right| \end{array}$$

(10b) $$\begin{array}{c} T_{V3}\left(X_{i,j}^{t+1}\right)=\left|\frac{X_{i,j}^{t+1}}{\sqrt{1+{\left(X_{i,j}^{t+1}\right)}^{2}}}\right|,\;T_{V4}\left(X_{i,j}^{t+1}\right)=\left|\frac{2}{\pi }\arctan\left(\frac{\pi }{2}X_{i,j}^{t+1}\right)\right| \end{array}$$

Once the probability value $P_{i,j}=T\left(X_{i,j}^{t+1}\right)$ is established, the discrete state transition is governed by one of three core structural binarization rules selected to alter the selection pressure of the search engine:

- Standard Binarization (STD): Directly evaluates probability against a threshold τ. The variable is activated if the probability exceeds the threshold, preserving a direct mapping of the continuous momentum:
  (11) $$X_{i,j}^{bin}=\left\{\begin{array}{cc} 1 & \mathrm{if}\;\tau \leq P_{i,j}\\ 0 & \mathrm{otherwise} \end{array}\right.$$
- Complement Binarization (COM): Functions as a mechanistic bit-flipping operator. If the probability criteria is met, the current binary coordinate is inverted, generating a dynamic cycle designed to break local stagnation:
  (12) $$X_{i,j}^{bin}=\left\{\begin{array}{cc} ∼X_{i,j}^{bin} & \mathrm{if}\;\tau \leq P_{i,j}\\ 0 & \mathrm{otherwise} \end{array}\right.$$
- Elitist Binarization (ELIT): Maximizes selective pressure by forcing the active coordinates to mimic the global best solution ($X_{best}$) directly, discarding redundant or unpromising variable activations:
  (13) $$X_{i,j}^{bin}=\left\{\begin{array}{cc} X_{best,j}^{bin} & \mathrm{if}\;\tau \leq P_{i,j}\\ 0 & \mathrm{otherwise} \end{array}\right.$$

### 3.2. Chaotic Discretization and Algorithmic Integration

The integration of chaotic maps into swarm intelligence and evolutionary metaheuristics has emerged as a highly prominent strategy to bypass the inherent limitations of standard stochastic optimization [38,39]. Traditionally, deterministic non-linear sequences generated by chaotic maps are used to replace standard pseudo-random number generators (PRNGs) that govern critical algorithmic phases, such as initial population distribution, local search perturbations, and adaptive step-size selection bounded within the [0,1] domain [4]. By injecting chaotic sequences into these random variables, the optimization process shifts from a memoryless, uncorrelated stochastic path to a dynamic trajectory driven by non-linear ergodicity, non-periodicity, and long-term multi-directional exploration [40,41]. This structural injection prevents the distribution saturation typical of uniform random sequences, effectively maintaining a healthy balance between macroscopic exploration and fine-tuned exploitation across multi-dimensional search spaces [42,43,44].

Fundamentally, the recent literature demonstrates that the performance improvements observed in swarm-based algorithms do not stem from the inherent unpredictability of chaos itself but rather from the distinct asymmetric probability distributions generated by these maps [45,46,47]. While a standard pseudorandom number generator strictly enforces a uniform distribution between [0,1], chaotic maps introduce specific biases that alter the threshold crossing frequencies. By replacing the uniform random variable with a chaotic sequence, the binarization framework fundamentally alters the statistical bias in the state transition phase. Consequently, the selection of a chaotic map acts as a directed mechanism for injecting a distinct yet necessary mathematical distribution that matches the selective pressure demanded by the underlying landscape morphology.

**Figure 2:**
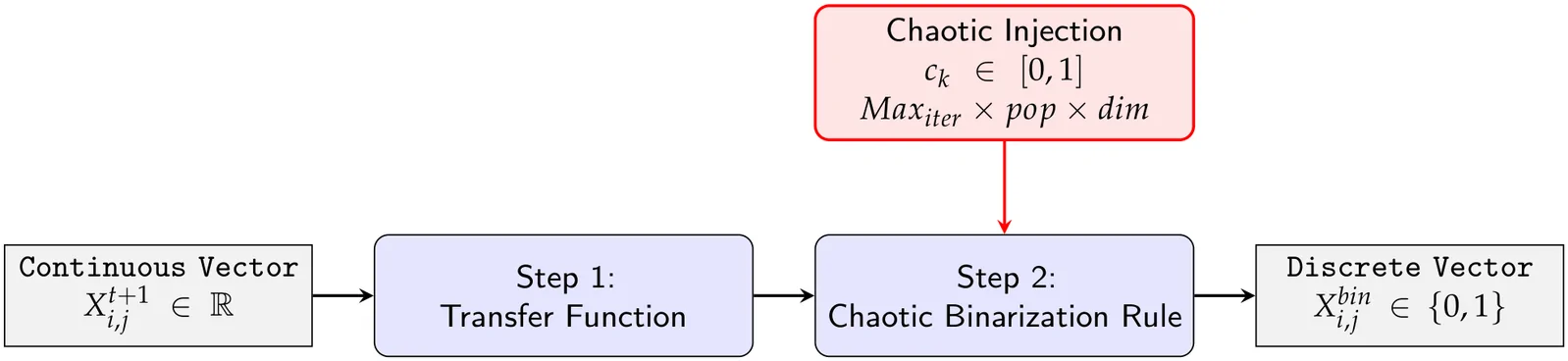
Proposed chaotic continuous-to-discrete pipeline highlighting the deterministic threshold injection ($c_{k}$).

This operational integration follows the chaotic binarization framework originally introduced by Cisternas-Caneo et al. [43], where the pseudo-random threshold τ∼U(0,1) within the external two-step interface is systematically replaced by a deterministic chaotic driver $c_{k}$. While its initial formulation was validated solely on a regularly graded, capacity-constrained landscape (the 0–1 Knapsack Problem), the present study rigorously tests and analyzes the behavioral dynamics of this paradigm across fundamentally different search-space topologies. Specifically, this work extends the framework to evaluate its structural resilience, adaptability, and preservation of diversity when confronting the severe spatial challenges posed by sparse matrices and mathematically degenerate zero-gradient flat plateaus. To maintain absolute operational synchronization across these diverse domains, the chaotic sequence is precomputed as a multi-dimensional tensor that matches the exact structural volume of the experiment, containing exactly $Max_{iter}\times pop\times dim$ sequential entries. The exact location of this chaotic threshold replacement inside the discretization pipeline is structurally mapped in Figure 2.

By incorporating the deterministic chaotic element $c_{k}$ directly into the conditional threshold logic, the canonical two-step state transitions are reformulated. This architectural replacement alters the selection pressure of the search engines, giving rise to the three core proposed chaotic binarization rules:

1. Chaotic Standard Binarization (CS-STD): Maps the continuous probability against the deterministic sequence, retaining a direct translation of the continuous velocity vectors while avoiding threshold saturation:
  (14) $$X_{i,j}^{bin}=\left\{\begin{array}{cc} 1 & \mathrm{if}\;c_{k}\leq T\left(X_{i,j}^{t+1}\right)\\ 0 & \mathrm{otherwise} \end{array}\right.$$
2. Chaotic Complement Binarization (CS-COM): Utilizes the chaotic orbit to govern a high-frequency bit-flipping operator, forcing structural disruptions designed to break convergence stagnation over mathematically degenerate flat basins:
  (15) $$X_{i,j}^{bin}=\left\{\begin{array}{cc} ∼X_{i,j}^{bin} & \mathrm{if}\;c_{k}\leq T\left(X_{i,j}^{t+1}\right)\\ 0 & \mathrm{otherwise} \end{array}\right.$$
3. Chaotic Elitist Binarization (CS-ELIT): Induces intense directional selection pressure by forcing active dimensions to rigorously replicate the current global best candidate coordinate ($X_{best}^{bin}$), optimizing variable pruning in sparse matrix representations:
  (16) $$X_{i,j}^{bin}=\left\{\begin{array}{cc} X_{best,j}^{bin} & \mathrm{if}\;c_{k}\leq T\left(X_{i,j}^{t+1}\right)\\ 0 & \mathrm{otherwise} \end{array}\right.$$

The combination of these three reformulated chaotic rules against the transfer functions establishes a highly adaptive discretization interface, allowing GWO, SCA, and WOA to preserve their distinct continuous properties while successfully navigating highly constrained landscapes.

## 4. Search-Space Morphologies and Problem Characterization

The systemic performance of any discrete optimization framework cannot be comprehensively understood without mapping the structural properties of its operating environment. Within combinatorics, a search space is formally conceptualized as a fitness landscape [48,49,50], comprising the set of all possible binary configurations within the hypercube vertex ${\left\{0,1\right\}}^{n}$ [51]. Evaluating discretization rules across diverse landscape configurations allows researchers to test the bounds of the No-Free-Lunch theorem. To rigorously stress-test the chaotic framework, this section defines three classic combinatorial problems characterized by radically different topological difficulties: capacity-constrained hyperplanes, ultra-sparse coverage matrices, and mathematically degenerate flat plateaus.

### 4.1. Capacity-Constrained Landscapes: 0–1 Knapsack Problem (KP)

From a structural perspective, the search space of the 0–1 Knapsack Problem (KP) is geometrically partitioned by the linear inequality boundary of its capacity constraint. Within the high-dimensional discrete hypercube ${\left\{0,1\right\}}^{n}$, the restriction $\sum w_{j}x_{j}\leq W$ defines a rigid linear hyperplane that separates the space into two distinct regions: a feasible subspace where solutions satisfy the weight limit and an infeasible domain where the capacity constraint is violated. Because the optimization goal is maximization, high-performing candidate solutions tend to cluster near the hyperplane boundary since maximizing profit requires using the available capacity *W* as fully as possible. Furthermore, the landscape features a distinct, informative fitness gradient directly derived from the objective function and constraint coefficients. Every discrete dimension *j* presents a specific efficiency ratio defined by profit per unit of weight ($p_{j}/w_{j}$). Consequently, a modification in the binary state vector produces an immediate and predictable shift in both total profit and weight allocation. This mathematical correlation provides directional cues that guide the continuous swarm agents toward promising regions, although the proximity of the optimal solutions to the feasibility boundary demands high trajectory precision to avoid entering the penalized infeasible domain.

Mathematically, given a set of *n* items, where each item *j* has a designated profit $p_{j}$ and a weight $w_{j}$, the objective is to select a subset of items to maximize the total profit without exceeding a maximum capacity *W*. The formulation is defined as follows:

(17) $$\mathrm{Maximize}\;f\left(X\right)=\sum_{j=1}^{n}p_{j}x_{j}$$

(18) $$\mathrm{Subject}\;\mathrm{to}\;\sum_{j=1}^{n}w_{j}x_{j}\leq W$$

where $X=[x_{1},x_{2},\ldots ,x_{n}]$ represents the discrete decision vector, with $x_{j}\in \left\{0,1\right\}$ indicating whether item *j* is selected ($x_{j}=1$) or rejected ($x_{j}=0$).

To illustrate this morphological constraint, consider a small instance with n=4 items, profits P=[40,30,20,10], weights $W_{items}=\left[4,3,2,1\right]$, and a knapsack capacity W=5. A continuous swarm agent might converge toward an optimal position yielding the binary vector $X_{A}=\left[1,0,0,1\right]$, which consumes exactly 4(1)+1(1)=5 units of capacity, achieving a maximum feasible fitness of 40(1)+10(1)=50. If a memoryless pseudo-random threshold causes a single bit-flip on the third dimension, mutating the vector to $X_{B}=\left[1,0,1,1\right]$, the capacity demand instantly escalates to 4+2+1=7. Because 7>5, this single modification pushes the agent past the strict feasibility edge into an infeasible zone, triggering heavy penalty functions that drop its fitness to zero. This numerical behavior demonstrates why the binarization rule must avoid erratic stochastic variations to preserve fragile, high-performing boundary combinations.

### 4.2. Sparse Coverage Landscapes: Set Covering Problem (SCP)

In contrast to the continuous hyperplane partition observed in the KP, the Set Covering Problem (SCP) introduces a highly restricted search space topology defined by a sparse binary constraint matrix $A_{m\times n}$. In typical combinatorial instances, this matrix exhibits a low density of active coefficients, where only 2% to 20% of the entries equal one ($a_{ij}=1$). Mathematically, this structural sparsity implies that most randomly generated binary configurations fail to satisfy the simultaneous row-coverage inequalities ($\sum_{j=1}^{n}a_{ij}x_{j}\geq 1$), resulting in a search space where the feasible region is highly constrained and difficult to sample uniformly. Furthermore, because the objective function targets cost minimization, the optimization process is heavily affected by variable redundancy. The “greater-than-or-equal-to” constraint in the inequality allows over-covered rows, inflating the total cost without improving feasibility. Consequently, the search landscape demands a high selection pressure to identify and prune these redundant column activations (1→0). Concurrently, due to the low density of ones in the matrix, specific rows are often covered by only a few critical columns; thus, the binarization interface must avoid erratic coordinate variations that could accidentally deactivate these essential variables and immediately drive the solution into the infeasible domain.

Formally, let M={1,2,…,m} be a set of rows to be covered and N={1,2,…,n} be a set of columns available to achieve the coverage, where each column j∈N carries a specific cost $c_{j}>0$. The binary matrix $A={\left[a_{ij}\right]}_{m\times n}$ defines the structural coverage, where $a_{ij}=1$ if row *i* is covered by column *j* and $a_{ij}=0$ otherwise. The mathematical optimization model is structured as follows:

(19) $$\mathrm{Minimize}\;f\left(X\right)=\sum_{j=1}^{n}c_{j}x_{j}$$

(20) $$\mathrm{Subject}\;\mathrm{to}\;\sum_{j=1}^{n}a_{ij}x_{j}\geq 1,\;\forall i\in M$$

where $x_{j}\in \left\{0,1\right\}$ is the decision variable tracking whether column *j* is integrated into the final covering solution.

To model this sparse behavior, consider an instance with m=3 rows and n=4 columns, with column costs C=[3,5,2,4], and a sparse binary coverage matrix defined as:

$$A=\left(\begin{array}{cccc} 1 & 0 & 0 & 1\\ 0 & 1 & 1 & 0\\ 0 & 0 & 1 & 1 \end{array}\right)$$

An active solution $X_{A}=\left[1,1,1,0\right]$ satisfies all constraints, since row 1 is covered by column 1 and rows 2 and 3 are covered by column 3, yielding a cost of 3(1)+5(1)+2(1)=10. However, column 2 is entirely redundant because row 2 is already taken care of by column 3. If a binarization rule lacks sufficient selective pressure to prune this dimension, the agent remains trapped in a sub-optimal state. Conversely, if a standard random threshold accidentally deactivates column 3 ($X_{B}=\left[1,1,0,0\right]$), row 3 instantly becomes completely uncovered because no other active column contains a 1 in that position. This numerical example shows that because the matrix is sparse, minor accidental bit changes can instantly render the system infeasible, which justifies using elite chaotic rules to target and prune redundant columns without dropping vital coverage.

### 4.3. Symmetrical Flat Landscapes: Unicost Set Covering Problem (USCP)

The Unicost Set Covering Problem (USCP) introduces a mathematically degenerate search landscape configuration derived directly from the homogenization of its objective function coefficients. By enforcing a strictly uniform cost vector where the weight of every column is fixed to unity ($c_{j}=1,\forall j\in N$), the objective function collapses into a direct cardinality metric that counts the total number of active columns ($\sum x_{j}$). Algebraically, this parameterization completely eliminates the informative cost gradient that typically guides optimization paths in general covering problems. As a consequence, the search space becomes characterized by extensive, symmetrical flat plateaus where diverse binary column combinations yield identical fitness costs, provided they share the same number of active variables. Because distinct search trajectories yield zero variation in fitness feedback, canonical continuous metaheuristics operating with memoryless uniform pseudo-random number generators suffer a complete loss of directional guidance. Without localized gradient indicators to distinguish between alternative feasible configurations, the swarm’s continuous step displacements lose structural orientation, causing agents to drift aimlessly across these zero-gradient basins and increasing susceptibility to premature convergence stagnation.

The mathematical model of the USCP inherits the exact row-coverage constraint matrix *A* from the standard SCP, but collapses the objective function into a direct sum of active columns, formulated as follows:

(21) $$\mathrm{Minimize}\;f\left(X\right)=\sum_{j=1}^{n}x_{j}$$

(22) $$\mathrm{Subject}\;\mathrm{to}\;\sum_{j=1}^{n}a_{ij}x_{j}\geq 1,\;\forall i\in M$$

where the elimination of individual column costs isolates the cardinality of the active subset as the sole optimization metric.

To demonstrate how this flat degeneracy stalls optimization, consider a matrix with m=3 rows and n=5 columns, where all costs are set to $c_{j}=1$. Let two distinct solution vectors be defined as $X_{A}=\left[1,0,1,0,0\right]$ and $X_{B}=\left[0,1,0,0,1\right]$, and assume that both configurations satisfy the row-covering constraints $\sum a_{ij}x_{j}\geq 1$. Evaluating their fitness yields $f(X_{A})=1+0+1+0+0=2$ and $f(X_{B})=0+1+0+0+1=2$. Even though $X_{A}$ and $X_{B}$ occupy completely opposite positions inside the high-dimensional hypercube, their fitness output is identical. If an algorithm lands on $X_{A}$, a uniform random threshold will see a flat gradient (2→2) and cannot determine whether moving toward $X_{B}$ is beneficial or harmful. The swarm stalls because the landscape gives no feedback. This numerical scenario shows why the high-frequency cyclic disruptions of chaotic maps under complementary operators are absolutely vital; they provide the non-periodic momentum needed to force the agents to break out of these deceptive flat basins.

## 5. Experimental Setup

To isolate and rigorously evaluate the performance of the chaotic binarization framework across contrasting search-space morphologies, a highly controlled and symmetric experimental environment is established. This section defines the mathematical characterization of the seven selected chaotic maps, the structural properties of the benchmark instances extracted from the literature, and the global parametric configurations enforced to guarantee a fair comparison among the updated metaheuristics.

### 5.1. Chaotic Map Characterization and Parameterization

The chaotic binarization framework systematically replaces the canonical pseudo-random number generator τ∼U(0,1) in the threshold validation phase with a deterministic chaotic sequence generator. The selection of the seven chaotic maps implemented in this framework is designed to cover a broad spectrum of mathematical dynamical behaviors. Rather than relying on trial-and-error parameter sweeps, the maps are chosen based on their underlying algebraic structures to ensure a diverse representation of non-linear threshold controls. This taxonomy encompasses quadratic dynamical systems (logistic map by Equation (23a)), higher-degree polynomial systems (Singer map by Equation (23d)), trigonometric systems (sine map by Equation (23c) and sinusoidal map by Equation (23e)), piecewise linear systems (piecewise map by Equation (23b) and tent map by Equation (23f)), and module-based systems (circle map by Equation (23g)). The mathematical definition of each of these chaotic maps is as follows:

(23a) $$\begin{array}{cc} \mathrm{Logistic}\;\mathrm{Map}:\;c_{k+1} & =\alpha c_{k}\left(1-c_{k}\right),\;\alpha =4, \end{array}$$

(23b) $$\begin{array}{cc} \mathrm{Piecewise}\;\mathrm{Map}:\;c_{k+1} & =\left\{\begin{array}{cc} \frac{c_{k}}{\lambda }, & 0\leq c_{k}<\lambda ,\\ \frac{c_{k}-\lambda }{0.5-\lambda }, & \lambda \leq c_{k}<0.5,\\ \frac{1-\lambda -c_{k}}{0.5-\lambda }, & 0.5\leq c_{k}<1-\lambda ,\\ \frac{1-c_{k}}{\lambda }, & 1-\lambda \leq c_{k}<1, \end{array}\right.\;\lambda =0.4, \end{array}$$

(23c) $$\begin{array}{cc} \mathrm{Sine}\;\mathrm{Map}:\;c_{k+1} & =\alpha \sin(\pi c_{k}),\;\alpha =1, \end{array}$$

(23d) $$\begin{array}{cc} \mathrm{Singer}\;\mathrm{Map}:\;c_{k+1} & =\mu \left(7.86c_{k}-23.31c_{k}^{2}+28.75c_{k}^{3}-13.302875c_{k}^{4}\right),\;\mu =1.07, \end{array}$$

(23e) $$\begin{array}{cc} \;\mathrm{Sinusoidal}\;\mathrm{Map}:\;c_{k+1} & =\alpha c_{k}^{2}\sin\left(\pi c_{k}\right),\;\alpha =2.3, \end{array}$$

(23f) $$\begin{array}{cc} \mathrm{Tent}\;\mathrm{Map}:\;c_{k+1} & =\left\{\begin{array}{cc} \frac{c_{k}}{0.7}, & c_{k}<0.7,\\ \frac{10}{3}\left(1-c_{k}\right), & c_{k}\geq 0.7, \end{array}\right. \end{array}$$

(23g) $$\begin{array}{cc} \mathrm{Circle}\;\mathrm{Map}:\;c_{k+1} & =\left(c_{k}+\delta -\frac{\alpha }{2\pi }\sin\left(2\pi c_{k}\right)\right)\;\mathrm{mod}\;1,\;\alpha =0.5,\;\delta =0.2. \end{array}$$

**Figure 3:**
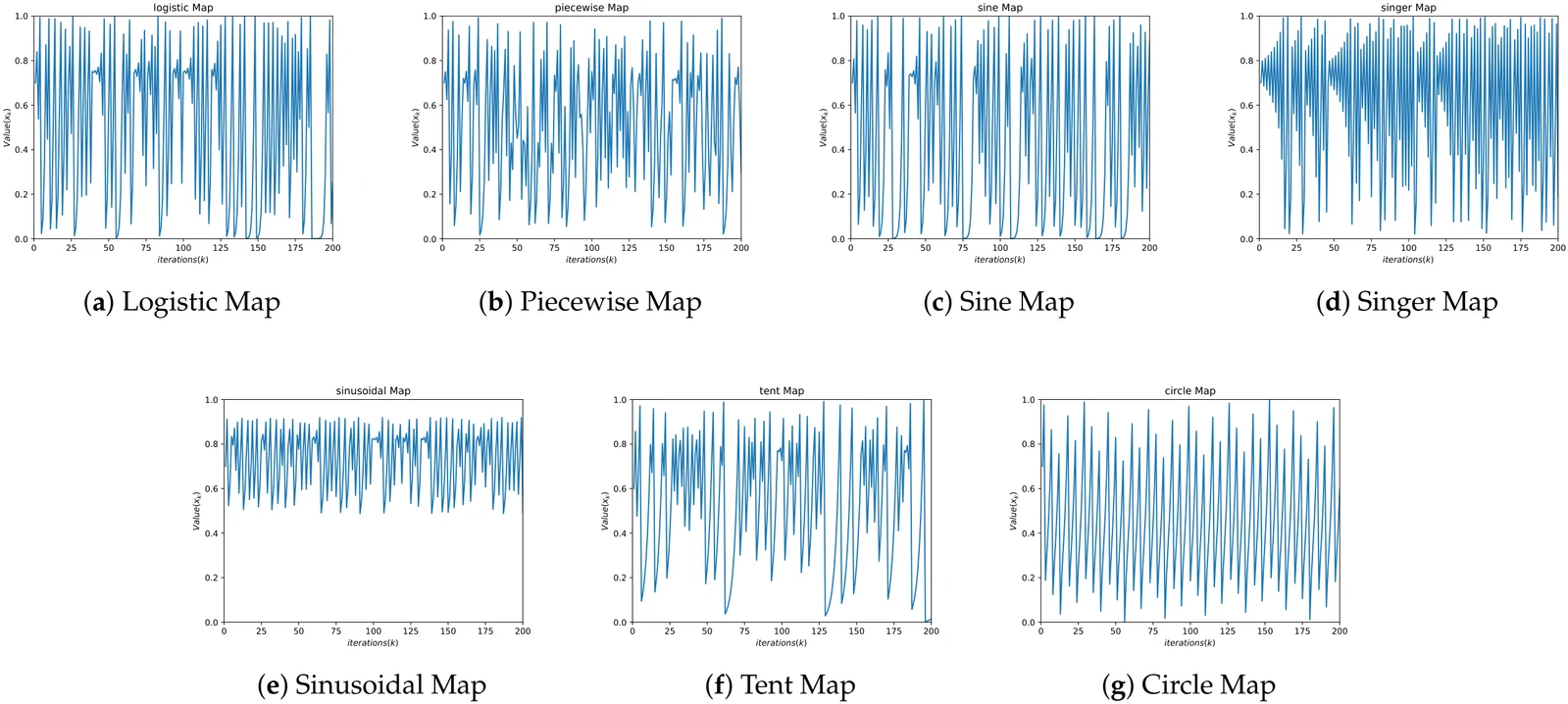
Chaotic maps.

To ensure that the chaotic trajectories exhibit fully developed bifurcation behavior and operational ergodicity, each map is rigorously parameterized according to the constants defined in the Equations (23a) to (23g). All chaotic maps are initialized with $s_{0}=0.7$, except for the chaotic map Tent, which starts with $s_{0}=0.6$. Figure 3 shows the chaotic sequence of each of these maps, projected up to 200 iterations. Each of these maps shows a distinct sequence that clearly highlights how the numbers in the interval [0, 1] are projected.

**Figure 4:**
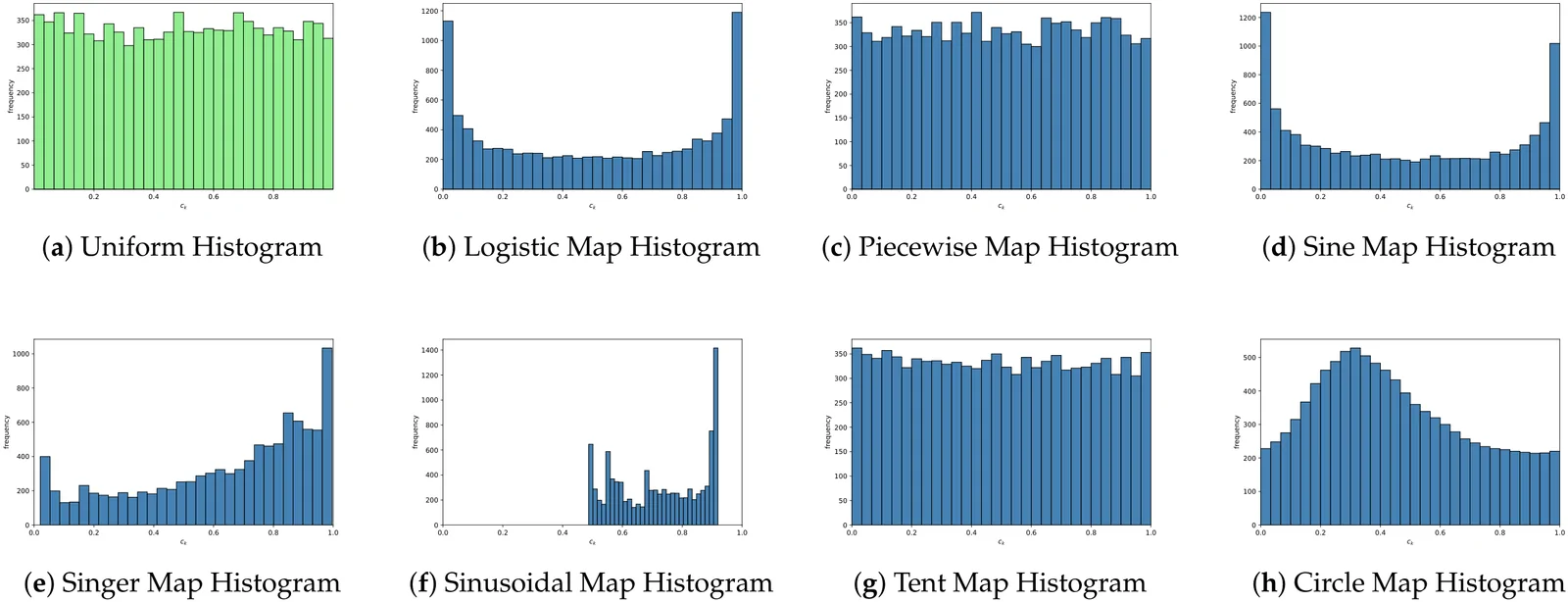
Histogram of the numbers obtained for each chaotic map and the uniform distribution between [0, 1] across 10,000 sequences.

A more in-depth analysis is shown in Figure 4, which displays a histogram obtained for each chaotic map and the uniform distribution in [0, 1] (highlighted in green) after sequencing 10,000 elements. This figure clearly shows that the uniform distribution (Figure 4a), the Piecewise chaotic map (Figure 4c), and the Tent chaotic map (Figure 4g) have similar histograms, in which all numbers between 0 and 1 show a similar frequency, indicating uniformity along the projected sequence of these chaotic maps.

On the other hand, the Logistic map (Figure 4b) and the Sine map (Figure 4d) show a clear tendency toward the extremes of the interval [0, 1]. Looking at the chaotic binarization rules presented in Section 3.2, the inflection point of each rule is the term $c_{k}\leq T\left(X_{i,j}^{t+1}\right)$. Thus, if we have a $c_{k}$ that mostly takes values of 0 or 1, this causes the rule to tend to be true or false in most cases due to the probability that $T(X_{i,j}^{t+1})$ takes values greater than or equal to $c_{k}$.

Then we have the Singer map (Figure 4e) and the Sinusoidal map (Figure 4f), which show a tendency to favor values greater than 0.5, especially the Sinusoidal map, which only has values between 0.5 and 0.9. Recalling the chaotic binarization rules again, we see that, due to probability, the rule tends to consider the term $c_{k}\leq T\left(X_{i,j}^{t+1}\right)$ as false.

Finally, the chaotic Circle map (Figure 4h) is the only chaotic map that shows a tendency towards values less than 0.5, grouping them between 0.2 and 0.5. If we do the cross-referencing with the chaotic binarization rules, we see that, by probability, the term $c_{k}\leq T\left(X_{i,j}^{t+1}\right)$; the binarization rule tends to favor the term being true.

These variations in the histograms will be validated in each binarization rule; therefore, in the present research, we experimented with a total of 24 binarization rules, corresponding to 8 standard binarization rules (7 chaotic plus the native version), 8 elite binarization rules (7 chaotic plus the native version) and 8 complementary binarization rules (7 chaotic plus the native version).

It is important to note that while the chaotic threshold tensor is precomputed deterministically to ensure experimental reproducibility, every independent run is initialized with a distinct, randomly generated population and unique PRNG seeds for the underlying metaheuristic operators. Consequently, each execution follows a divergent continuous trajectory, ensuring that the agents sample the chaotic threshold stream at unique points and intervals. This hybrid stochastic–deterministic approach preserves the statistical independence among the 31 execution runs, as the search-path variability is governed by the metaheuristics’ intrinsic stochastic processes, while the chaotic binarization serves as a consistent topological regulator.

### 5.2. Benchmark Problem Instances

To validate the behavioral dynamics and structural resilience of the chaotic binarization framework under severe combinatorial stress, the experimental evaluation incorporates large-scale test datasets extracted from prominent repositories in the literature. Each selected problem paradigm represents a distinct structural configuration matching the specific fitness landscape morphologies characterized in Section 4.

For the capacity-constrained landscapes, fifteen 0–1 Knapsack Problem (KP) instances are selected from Pisinger’s standardized test set [52]. These instances are classified into three distinct categories based on the correlation coefficient between item weights and profits, representing different operational difficulty tiers. In the Uncorrelated Instances (prefix knapPI_1), weights $w_{j}$ and profits $p_{j}$ are generated independently within predefined ranges. Because the search space exhibits fewer deceptive local optima regarding the profit-to-weight ratio, selection criteria do not involve conflicting item attributes. In the Weakly Correlated Instances (prefix knapPI_2), item profits are moderately related to their respective weights ($p_{j}\in \left[w_{j}-R/10,w_{j}+R/10\right]$), which introduces greater complexity and requires a more nuanced spatial search to identify optimal item combinations. Finally, the Strongly Correlated Instances (prefix knapPI_3) represent the most challenging optimization scenarios, where profits are strictly constrained as $p_{j}=w_{j}+K$, with *K* being a small constant. These instances feature a narrow search space of high-quality solutions, making them ideal for testing the capability of chaotic binarization rules to prevent rapid selection saturation. The detailed profile of these fifteen KP instances is summarized in Table 1.

To evaluate the discretization layer against sparse coverage landscapes, nine classical instances of the Set Covering Problem (SCP) are extracted from the standardized OR-Library [53]. These benchmarks vary extensively in terms of row constraints (*m*), variable dimensionality (*n*), and structural matrix density, with active coefficient concentrations spanning from 2.00% up to 20.00%. To facilitate a rigorous comparative analysis of selective pressure effects, a homogeneous column cost range of [1,100] is systematically maintained across all instances [6], as outlined in Table 2.

Finally, the framework’s capability to operate within symmetrical flat landscapes is stress-tested using eleven benchmark instances of the Unicost Set Covering Problem (USCP), incorporating three distinct structural topologies: homogeneous-weight conversions, patterned configurations, and cyclic networks [6]. The first category is constructed by directly transforming the core subset of standard SCP matrices and adjusting all column costs to unity ($c_{j}=1$), isolating cardinality as the sole performance metric [54]. The Structured Instances (uclr10–uclr13), derived from the OR-Library, feature constraints with regular patterns that exhibit increasing sparsity scaling proportionally with problem size. In contrast, the Cyclic Instances (ucyc06–ucyc07) represent complex network design challenges characterized by circular coverage structures, explicitly designed to evaluate the algorithms’ ability to break local stagnation in the presence of strict circular dependencies. For the structured and cyclic datasets, the true global optima remain unproven in the literature; hence, the best-reported values are integrated as baseline metrics (denoted in boldface and italics). The comprehensive structural properties of the USCP datasets are provided in Table 3.

### 5.3. Algorithmic Configuration and Computational Environment

Due to the distinct structural complexities and dimensional boundaries of the selected benchmark problems, the global computational budget was dynamically adapted based on extensive preliminary laboratory experiments. For the KP instances, a population size of 20 search agents operating over a maximum of 500 iterations provided an optimal balance for navigating the deceptive capacity constraints. Conversely, the SCP and USCP instances have high dimensionality, with up to 10,000 decision variables. For these covering problems, preliminary tuning revealed that restricting the computational budget to a compact population of 10 agents evolving over 100 iterations was highly beneficial. This restricted budget prevents excessive computational overhead and forces the metaheuristics to rely heavily on the exploitative and explorative efficiency of the chaotic binarization mechanisms rather than brute-force computational cycles, thereby isolating and highlighting the true impact of the proposed framework.

To ensure statistical robustness given the stochastic nature of the underlying algorithms, each of the 72 algorithmic configurations (3 MH × 24 binarization rules) was executed 31 times independently for each benchmark instance. This design strictly adheres to the requirements of the Central Limit Theorem, enabling the subsequent application of robust non-parametric statistical tests [6]. Thus, the present research contemplates a total of 78,120 experiments, of which 33,480 correspond to the Knapsack Problem (3 metaheuristics · 24 binarization schemes · 15 instances · 31 independent executions), 20,088 to the Set Covering Problem (3 metaheuristics · 24 binarization schemes · 9 instances · 31 independent executions), and 24,552 to the Unicost Set Covering Problem (3 metaheuristics · 24 binarization schemes · 11 instances · 31 independent executions).

The internal parameters governing the continuous operators of the base metaheuristics were selected in strict accordance with the standard recommendations provided by their respective original authors [8,9,10]. A comprehensive summary of these unified parameter settings is presented in Table 4. The choice of population size and number of iterations is the result of a previously performed experimental adjustment.

Crucially, the parameterization of the discretization layer follows a strict morphological rationale derived from the established literature. For the capacity-constrained KP, we employ the gradual monotonic S-shaped transfer function ($T_{S2}$), which, as validated by Cisternas-Caneo et al. [43], preserves the granular trajectory precision required to navigate near tight feasibility boundaries, such as those in the KP. Conversely, for sparse, mathematically degenerate flat landscapes such as the SCP and the USCP, the framework discards S-shaped functions in favor of the symmetric V-shaped transfer function ($T_{V3}$). This choice follows the seminal recommendation by Lanza et al. [6], where the hyperbolic square-root growth profile of $T_{V3}$ was shown to provide the exact selection pressure needed to prune redundant column activations in sparse matrices and force exploration across zero-gradient plateaus. Therefore, to isolate the regulatory impact of the chaotic threshold stream and avoid structural confounding variables, the parameterization of the discretization layer treats the transfer functions as pre-validated operational constants specific to each problem class, rather than as independent treatment variables.

Regarding computational overhead, the proposed chaotic discretization layer does not impose penalties on runtime performance. Because the non-linear chaotic sequences are precomputed as a multi-dimensional tensor matching the trial volume ($Max_{iter}\times pop\times dim$), the threshold validation phase replaces runtime mathematical map iterations with direct array indexing. Consequently, the time complexity of the discretization step remains bounded at O(1) per coordinate evaluation, mirroring the exact computational footprint of standard uniform pseudo-random number generator (PRNG) streams.

To empirically validate this theoretical O(1) efficiency, a comprehensive runtime analysis was conducted across the entire benchmark suite. We compared the mean wall-clock execution time of the chaotic-wrapper framework with the baseline uniform approach for each instance and metaheuristic configuration. As evidenced by the detailed results in Appendix C, the execution time difference between the two approaches is negligible, remaining well within the expected variance of standard system process scheduling. This empirical runtime data confirms that the chaotic discretization layer introduces no performance bottlenecks and successfully maintains the efficiency required for scalable combinatorial optimization.

All optimization engines, chaotic tensor precomputations, and microspatial data-logging loops were implemented natively in Python (version 3.10.5), leveraging NumPy and Pandas for data manipulation and computational throughput. The parallel experimental trials were compiled and executed in a high-performance decentralized workstation running Ubuntu Linux, powered by an Intel Core i9-14900K processor and equipped with 64 GB of DDR5 RAM operating at 6000 MT/s. This strict hardware homogeneity and environmental parameterization ensure that all convergence-tracking records, diversity metrics, and execution profiles remain entirely free from computational or environmental biases while providing consistent execution times and reliable data logging.

### 5.4. Performance Metrics and Statistical Validation

To execute a comprehensive, multi-layered validation of the chaotic binarization framework, the experimental analysis is divided into two operational dimensions supported by a robust non-parametric statistical validation suite.

#### 5.4.1. Macroscopic Accuracy Metric

The primary objective of the macroscopic analysis phase is to function as a high-efficiency tactical filter. Given the massive combinatorial volume of data generated across the experimental trials, evaluating every single configuration at a microscopic level would be computationally and analytically redundant. Consequently, the macroscopic review aggregates overall accuracy behaviors to identify and select the dominant families of chaotic binarization schemes, discarding sub-optimal variants before advancing the elite configurations to the advanced microspatial tracking stages.

To govern this selection process, the optimization accuracy and convergence stability across the R=31 independent runs are unified and quantified utilizing the Relative Percent Deviation (RPD). Instead of computing an aggregate metric from a single best-case scenario or a global final average, the evaluation process transparently calculates individual deviations for each execution. For any independent run r∈{1,2,…,R} within a capacity-constrained maximization landscape (KP), the individual percent error is formulated as follows:

(24) $$RPD_{max,i}^{r}=100\times \left(\frac{Opt_{i}-Fit_{i}^{r}}{Opt_{i}}\right)$$

where $Opt_{i}$ denotes the known theoretical global optimum for instance *i* and $Fit_{i}^{r}$ represents the final objective fitness achieved by the evaluated algorithmic variant during the specific run *r*. Once the complete array of individual errors is compiled, the final metric reported in our performance matrices is determined by computing the mathematical mean across the total execution horizon:

(25) $$RPD_{max,i}=\frac{1}{R}\sum_{r=1}^{R}RPD_{max,i}^{r}$$

Under this maximization topology, because any feasible solution is strictly bounded by the global maximum ($Fit_{i}^{r}\leq Opt_{i}$), the individual deviations are non-negative ($RPD_{max,i}^{r}\geq 0$). Consequently, a reported final $RPD_{max,i}$ of 0.0% functions as a strict indicator of absolute convergence stability, mathematically proving that the algorithm successfully hit the global optimum in 100% of its independent random initializations.

Conversely, because the SCP and USCP are strict minimization problems, the operational formulation is inherently inverted to evaluate how far the individual costs rise above the theoretical minimum. For any independent execution run r∈{1,2,…,R}, the individual percent deviation is defined as follows:

(26) $$RPD_{min,i}^{r}=100\times \left(\frac{Fit_{i}^{r}-Opt_{i}}{Opt_{i}}\right)$$

where $Opt_{i}$ denotes the known theoretical global minimum cost for instance *i* and $Fit_{i}^{r}$ represents the total column or cardinality cost accumulated by the algorithm during run *r*. Following the same protocol, the final macroscopic index reported in our benchmark matrices represents the arithmetic mean of these individual values:

(27) $$RPD_{min,i}=\frac{1}{R}\sum_{r=1}^{R}RPD_{min,i}^{r}$$

Under this minimization layout, since the cost of any feasible covering combination is stochastically and algebraically bounded from below by the global optimum ($Fit_{i}^{r}\geq Opt_{i}$), the individual components are strictly non-negative ($RPD_{min,i}^{r}\geq 0$). Consequently, a final averaged $RPD_{min,i}$ of 0.0% provides an absolute guarantee of multi-run stability, confirming that the updated search engine successfully converged to the absolute minimum cost in 100% of the 31 independent random initializations without a single outlier or sub-optimal activation.

#### 5.4.2. Microspatial Behavioral Tracking and Statistical Protocol

While macroscopic evaluation via Relative Percent Deviation (RPD) serves as a highly efficient structural filter for isolating dominant binarization families, aggregated final accuracy metrics cannot capture the real-time shifts that drive particle swarm trajectories. To gain a deep understanding, a microscopic analysis is required.

##### Convergence Velocity and Stability Profiling

This protocol monitors the evolutionary trajectory of the best-so-far fitness value at each discrete iteration *t*. The objective is to visually analyze the optimization speed and trajectory stability, identifying the exact generational milestones where alternative binarization configurations trigger premature stagnation or maintain active optimization momentum.

##### Exploration–Exploitation Phase Balances

To quantify the spatial kinematics of the population, the framework tracks real-time diversity at each iteration *t* using the dimension-wise metric [55]:

(28) $$Div\left(t\right)=\frac{1}{N\cdot D}\sum_{i=1}^{N}\sum_{j=1}^{D}\left|x_{i,j}^{t}-{\bar{x}}_{j}^{t}\right|$$

where *N* is the population size, *D* is the variable dimensionality, $x_{i,j}^{t}$ represents the coordinate position, and ${\bar{x}}_{j}^{t}$ is the instantaneous population mean of the *j*-th dimension.

Building on this indicator, the dynamic balance between the diversification and intensification phases is quantified according to the formulation by Morales-Castañeda et al. [12]. This approach resolves the explicit percentages of exploration (XPL%) and exploitation (XPT%) by normalizing the current state against the maximum historical diversity ($Div_{max}$):

(29) $$XPL\left(\%\right)=\frac{Div(t)}{Div_{max}}\times 100$$

(30) $$XPT\left(\%\right)=\frac{|Div\left(t\right)-Div_{max}|}{Div_{max}}\times 100$$

where $Div_{\max}=\max_{t\in \{1,\ldots ,T\}}Div\left(t\right)$ acts as the structural scaling baseline across the total execution horizon *T*. This metrics-driven protocol isolates whether a specific chaotic map sustains macroscopic space diversification or accelerates micro-local coordinate refinement.

##### Non-Parametric Statistical Arbitration

To mathematically validate the performance differences and discard stochastic anomalies, a two-phase non-parametric statistical assessment is enforced at a significance level of α=0.05:

1. Friedman Test: Executes a multi-comparison variance evaluation across the entire benchmark suite to establish a global hierarchical ranking and identify the dominant chaotic binarization families.
2. Mann-Whitney U Test with Holm–Bonferroni Correction: Performs localized pairwise comparisons between high-performing chaotic variants and their respective uniform pseudo-random baselines. This test was chosen to evaluate the statistical significance of the architectural enhancements by comparing the distributions of the 31 independent execution runs as unpaired, independent samples. To control the family-wise error rate (FWER) inherent in multiple pairwise comparisons, all calculated *p*-values were adjusted using the Holm–Bonferroni method, and significance is reported at the α=0.05 level after correction.

## 6. Experimental Results and Discussion

To evaluate the performance of the chaotic binarization framework without architectural biases, the data compiled from all independent computational trials are systematically analyzed utilizing a modular, cascade-based validation approach. This section is structured strictly by search-space morphology, isolating the capacity-constrained landscapes (KP), sparse-coverage matrices (SCP), and mathematically degenerate flat plateaus (USCP) as independent analysis domains.

### 6.1. Evaluation on Capacity-Constrained Landscapes: The 0–1 Knapsack Problem

The macroscopic evaluation begins with a comparative analysis of the final Relative Percent Deviation (RPD) metrics across the fifteen 0–1 Knapsack Problem (KP) instances. Table 5 unifies the performance vectors of the 24 unique configurations across the GWO, SCA, and WOA frameworks. The green color in the table indicates that a metaheuristic configuration achieved an average RPD of 0.0% across 31 independent runs of a single instance; that is, all runs reached the known optimum. Conversely, the orange color indicates that a metaheuristic configuration achieved the lowest average RPD in a single instance. Additionally, Table A1, Table A2 and Table A3 show the performance of the algorithms in greater detail.

The compiled results demonstrate that the choice of the binarization rule family defines a clear performance boundary across all tested metaheuristics. The Complement (COM) and Elitist (ELIT) rule families consistently exhibit highly suboptimal behavior under the linear capacity constraints (*W*) in this landscape. Specifically, the COM family generates baseline average RPD values between 4.74% and 5.77%, while the ELIT family registers higher errors ranging from 6.52% to 6.83% across the global instance suite.

In contrast, the Standard (STD) binarization family proves significantly superior in capacity-constrained domains, yielding remarkably lower baseline average errors (1.09% for GWO, 1.36% for SCA, and 0.26% for WOA). More importantly, the integration of chaotic dynamics within the standard framework unmasks a clear dominant operator. The STD_CIRCLE configuration emerges as the top-performing mechanism within the standard group across all three metaheuristic architectures. When driven by the Circle map, GWO optimizes its global average RPD to 0.50%, SCA reduces its average deviation to 0.46%, and WOA achieves a minimized global error of 0.10%. This specific chaotic variant consistently locates the absolute global optimum (RPD=0.0%) across multiple uncorrelated and weakly correlated instances, demonstrating that deterministic non-linear threshold sequences significantly improve upon the classical uniform pseudo-random baseline limits.

The structural dominance of the STD_CIRCLE configuration can be mathematically explained by the observed bias in its probability distribution (see Figure 4h). Because the Circle map concentrates its threshold values ($c_{k}$) tightly between 0.2 and 0.5, it completely avoids the extreme polarization of U-shaped distributions, such as those of the Logistic or Sine maps. Under the standard binarization rule ($c_{k}\leq T\left(X_{i,j}^{t+1}\right)$), this probability mass exerts a moderately permissive selection pressure. It allows search agents to maintain a constant, fine rate of coordinate updates, thereby promoting mobility, without triggering the massive, simultaneous bit-swapping that would instantly push high-quality candidate solutions beyond the strict linear capacity limit (*W*) into the heavily penalized infeasible domain.

Based on the conclusive performance divergence established in Table 5, the highly disruptive COM and strongly biased ELIT binarization families are structurally unsuited for navigating the tight feasibility boundaries of the KP landscape. Consequently, to isolate and accurately measure the true impact of deterministic non-linear dynamics without analytical redundancy, the remainder of this microscopic evaluation discards the COM and ELIT rules entirely. The subsequent microspatial tracking phases focus exclusively on an in-depth behavioral scrutiny of the high-performing Standard (STD) family and its chaotic variants.

To examine these optimization physics over time, the microscopic analysis tracks the evolutionary trajectories of the swarms. To isolate the effects of structural scaling and correlation complexity, the analysis focuses on the high-dimensional 2000-item instances under Uncorrelated (1_2000) and Strongly Correlated (3_2000) conditions, directly pitting the classical uniform stochastic baseline (STD) against the globally dominant chaotic variant (STD_CIRCLE).

**Figure 5:**
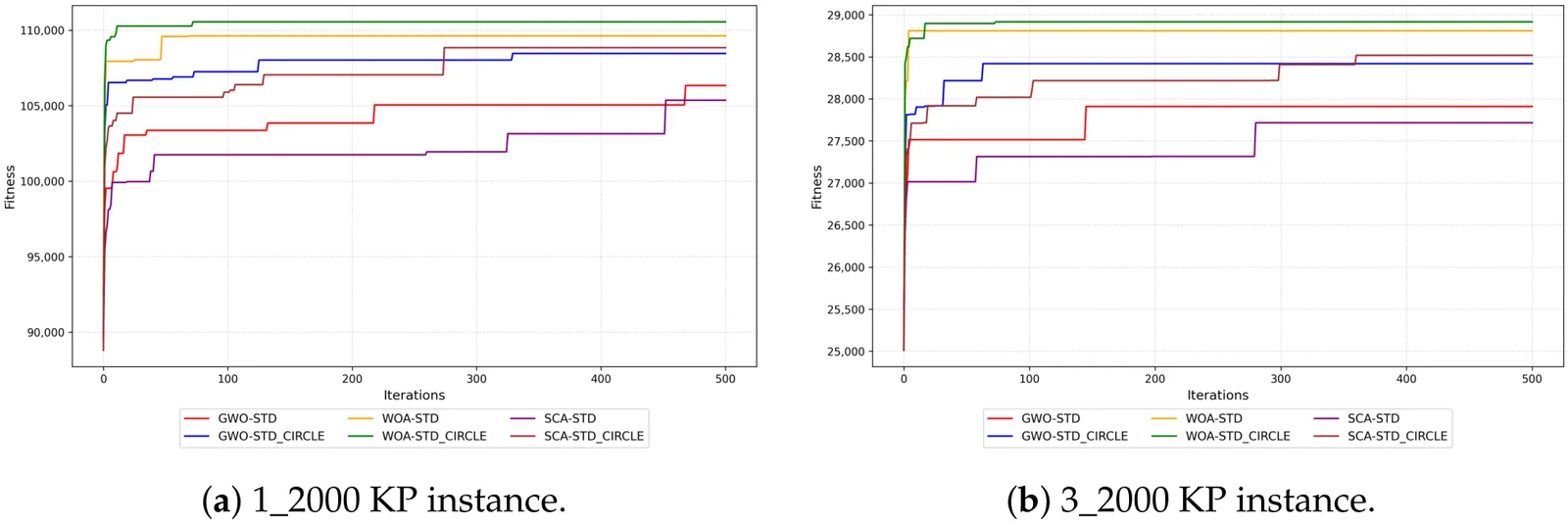
Convergence trajectories for the uncorrelated 1_2000 and strongly correlated 3_2000 KP instances.

Figure 5a illustrates the convergence behavior for instance 1_2000. The graphical trajectories show that while the uniform stochastic variant (WOA-STD) prematurely stagnates near iteration 50, the chaotic integration (WOA-STD_CIRCLE) leverages the ergodic properties of the Circle map to sustain active exploration, executing a decisive refinement leap prior to iteration 100. A similar qualitative shift is visible in GWO and SCA. The traditional baselines (GWO-STD and SCA-STD) suffer from prolonged horizontal stagnation plateaus. By replacing the pseudo-random number generator (PRNG) with the Circle map, GWO-STD_CIRCLE and SCA-STD_CIRCLE exhibit significantly steeper initial descent trajectories and a continuous capacity for coordinate fine-tuning throughout the search horizon.

This operational resilience is further confirmed in the strongly correlated 3_2000 instance (Figure 5b), where item profits are strictly proportional to their weights, creating a highly deceptive landscape. The classical stochastic variants exhibit extensive flat lines; for example, SCA-STD remains trapped in a sub-optimal state for over 200 iterations, illustrating premature convergence. In contrast, the integration of the Circle map enables SCA-STD_CIRCLE to replace this flatline stagnation with a distinct staircase progression, continually breaking out of local traps and improving fitness well beyond iteration 300. Similarly, WOA-STD_CIRCLE executes critical refinement jumps during the initial phase, ultimately plateauing at a superior fitness level.

**Figure 6:**
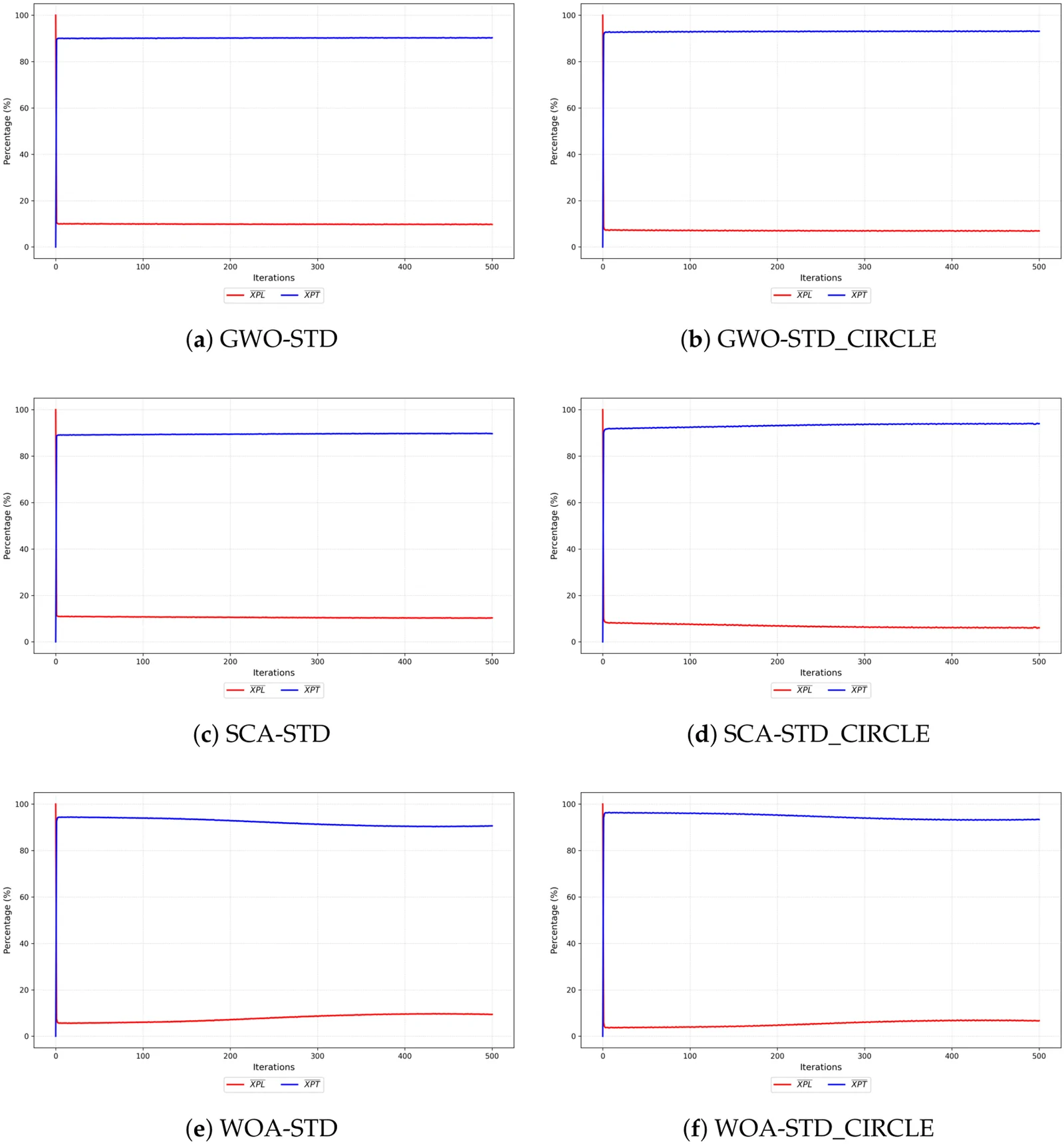
Exploration and exploitation dynamics for all metaheuristics on the 3_2000 instance.

To examine the underlying phase mechanics, the temporal exploration (XPL) and exploitation (XPT) percentages are monitored for the 3_2000 instance using the dimension-wise diversity metric. For Figure 6a–f, the horizontal axis indicates the optimization iterations, while the vertical axis represents the phase percentage. Additionally, they represent shaded scatter bands rather than single lines, capturing the variation across the 31 independent runs. This structure allows a simultaneous analysis of both the strategic phase change and the algorithmic stability through the thickness of the scattering bands.

As plotted in Figure 6, all six algorithmic configurations exhibit nearly identical behavior: an almost instantaneous collapse from 100% exploration at initialization to a sustained state of deep exploitation (XPT>90%) for the remainder of the optimization process. This uniformity is directly driven by the interaction between the high-dimensional scaling and the hyper-constrained topology of the strongly correlated KP landscape. At initialization, random binary distribution forces maximum coordinate dispersion across the hypercube, establishing a massive historical $Div_{max}$ baseline. However, as derived in Section 4, achieving high-quality feasible solutions requires the agents to immediately cluster near the rigid linear boundary of the capacity constraint.

When 2000 decision variables simultaneously compress their search radii to fit this narrow boundary neighborhood, the absolute diversity value undergoes a steep, order-of-magnitude reduction. Mathematically, because the phase allocation formula normalizes every state against the massive initial $Div_{max}$, this sharp dimensional contraction forces the metric to register absolute exploitation almost instantly. Consequently, the metric becomes numerically blind to the critical micro-oscillations, trajectory-step corrections, and chaotic perturbations that occur in the boundary neighborhood. This behavior demonstrates that while standard phase-percentage metrics capture large-scale spatial collapse, they lack the resolution required to characterize the fine-grained inner mechanics of chaotic binarization in heavily restricted combinatorial spaces.

To mathematically validate these performance shifts, the final fitness values undergo a two-phase non-parametric statistical assessment. The global Friedman test is conducted across all fifteen KP instances under the null hypothesis ($H_{0}$) that the performance distributions of the evaluated algorithms are not significantly different, against the alternative hypothesis ($H_{1}$) that at least one binarization configuration exhibits a structurally distinct performance distribution. The computational validation yielded an overall *p*-value of $2.8813\times 10^{-4}$. Since this value is strictly below the significance level (α=0.05), the null hypothesis is rejected, confirming that substituting the PRNG with chaotic maps induces statistically significant changes in algorithmic capabilities.

The directional rankings assigned by the Friedman test are reported in Table 6. The green color in the Table indicates that one configuration obtained the first position after applying the Friedman test. The orange color indicates that more than one configuration obtained the first position. For GWO and SCA, the integration of the Circle map systematically enhances global performance, with SCA-STD_CIRCLE shifting its average rank from 4.70 to 3.30. For the WOA framework, while the baseline WOA-STD achieves a highly competitive average rank (2.50) due to frequent optimization ties in small-scale instances, the chaotic WOA-STD_CIRCLE systematically secures the absolute first-place rankings (rank = 1.0) in the high-dimensional, strongly correlated scenarios (1_1000, 1_2000, 2_2000, 3_1000, and 3_2000).

The pairwise validation is completed via the directional Mann–Whitney U test at α=0.05. To control the family-wise error rate (FWER) inherent in multiple pairwise comparisons, all reported *p*-values were adjusted using the Holm–Bonferroni method. Table 7 outlines the resulting pairwise win matrix, counting the number of instances where the row algorithm statistically outperformed the column algorithm after the correction. The diagonal intersections show that the chaotic variants achieve decisive superiority over their direct stochastic baselines: GWO-STD_CIRCLE defeats its baseline in 7 out of 15 instances, SCA-STD_CIRCLE dominates its baseline in 8 out of 15 instances, and WOA-STD_CIRCLE mathematically outperforms its baseline in 5 out of 15 instances.

Furthermore, the matrix confirms that the underlying WOA architecture is statistically dominant for this problem topology, imposing a heavy win rate against all GWO and SCA variants. Ultimately, WOA-STD_CIRCLE consolidates its position as the pinnacle configuration for capacity-constrained landscapes, successfully hybridizing the continuous exploitation spirals of the base whale operator with the deterministic thresholding of the Circle map to maximize solution quality.

In short, STD_CIRCLE solidifies its position as the most effective configuration for the 0–1 Knapsack Problem. As statistical and visual evidence demonstrates, this superiority is not due to unpredictable chaotic noise but rather to the specific non-polarized distribution of the Circle map, which provides the precise microspatial tuning necessary to safely navigate the fragile viability limits of the knapsack topology.

### 6.2. Evaluation on Sparse Coverage Landscapes: The Set Covering Problem

The analytical cascade progresses to the SCP. Table 8 unifies the macroscopic RPD metrics compiled across the nine instances for the GWO, SCA, and WOA frameworks. Empirical data reveal a profound topological paradigm shift: the sparse density matrices of the SCP alter the structural effectiveness of binarization rule families. The green color in the table indicates that a metaheuristic configuration achieved an average RPD of 0.0% across 31 independent runs of a single instance; that is, all runs reached the known optimum. Conversely, the orange color indicates that a metaheuristic configuration achieved the lowest average RPD in a single instance. Additionally, Table A4, Table A5 and Table A6 show the performance of the algorithms in greater detail.

The macroscopic evaluation across the nine Beasley instances reveals a pervasive failure within the Complement (COM) family and establishes the absolute dominance of the Elitist (ELIT) binarization family over the Standard (STD) mechanisms. The indiscriminate inversions of the COM operator activate redundant pathways excessively, causing uncontrolled column inflation and leading to highly suboptimal average errors that peak at 7.68% in GWO and 6.14% in WOA. In contrast, the ELIT rule structures discrete updates by biasing the thresholding phase directly toward the coordinates of the lowest-cost agent, generating a highly focused exploitation dynamic that effectively minimizes the overall column cost. Consequently, the ELIT family outclasses the baseline stochastic STD family, with deviations of 2.89% for GWO, 3.34% for SCA, and 3.20% for WOA, capturing the vast majority of absolute theoretical minimum costs where the RPD equals 0.0%. Within this elite group, the ELIT_SINU variant driven by the chaotic Sinusoidal map achieves the absolute lowest global average deviations, minimizing the final RPD error to 2.23% in both the SCA and WOA execution suites.

This macroscopic superiority can be directly explained by combining the SCP constraints with the probability distributions of the chaotic maps detailed in Section 5.1. In highly sparse matrices, minimizing the objective cost requires aggressively pruning redundant active columns (1⟶0) without destroying the fragile row coverage. As shown in Figure 4f, the sinusoidal map strongly concentrates the sequence values ($c_{k}$) between 0.5 and 0.9. Under the ELIT rule formulation, this high spatial bias makes the transition condition ($c_{k}\leq T\left(X_{i,j}^{t+1}\right)$) statistically more difficult to satisfy. Consequently, the threshold mechanism is mathematically forced into its “otherwise” state ($X^{bin}=0$) much more frequently. This distinctive distribution asymmetry acts as a targeted, high-intensity pruning mechanism, aggressively reducing the number of columns more effectively than a uniform, flat distribution.

Following this conclusive macroscopic performance boundary, highly disruptive COM rules and unfocused STD mechanisms are discarded from subsequent evaluations to avoid analytical redundancy. To preserve analytical depth and isolate the mechanical impact of chaotic drivers on sparse coverage landscapes, all subsequent microscopic tracking protocols focus exclusively on the dominant ELIT binarization family.

**Figure 7:**
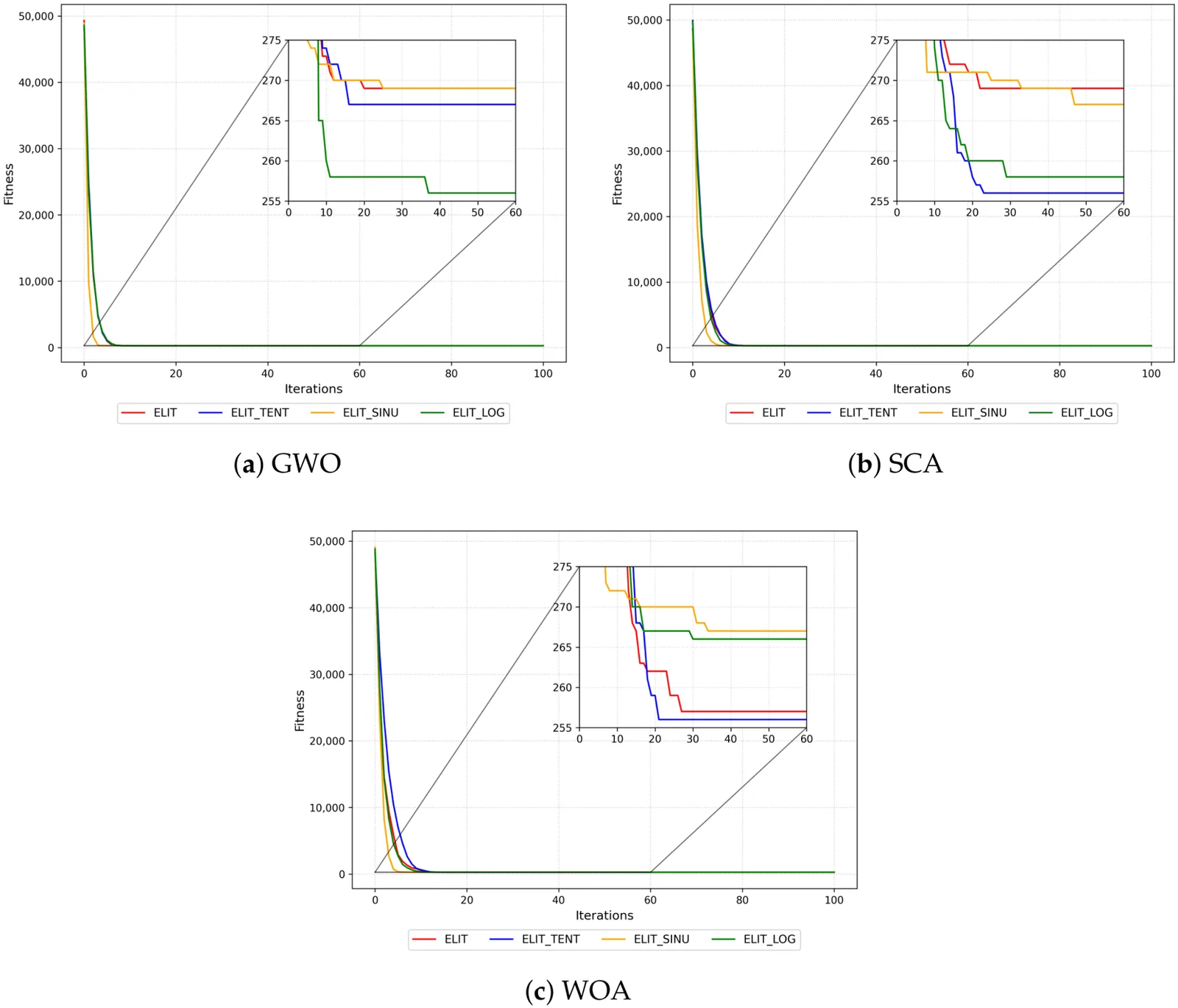
Convergence trajectories for the SCP instance 51.

To examine convergence speed, instance 51 will be used, as its performance profiles capture the global trends observed in the reference set. As illustrated in Figure 7, the Elitist (ELIT) binarization rule, in both its standard and chaotic versions, induces a sharp decline in convergence across all metaheuristics before the first 20 iterations. The enlarged windows reveal the specific micro-exploitation advantages introduced by the chaotic operators. Within the GWO framework, the chaotic variant ELIT_LOG decisively dominates the trajectory by entering the exploitation phase with a lower column cost, while the remaining configurations stagnate. Conversely, the SCA and WOA frameworks highlight the mechanical superiority of the Tent map, as the ELIT_TENT configuration performs a descent that actively breaks early local stagnation plateaus to achieve the lowest final costs.

The trajectories confirm that the most effective chaotic driver is strictly algorithm-dependent, a phenomenon rooted in the way that each map’s probability distribution interacts with continuous vector updates. For GWO, the logistic map’s U-shaped distribution (Figure 4b) pushes thresholds toward the extremes, polarizing binary decisions and perfectly complementing the rigid hierarchical fence of multiple wolfpack leaders. Conversely, the shop map acts as the ultimate topological specialist for SCA and WOA. Because its distribution (Figure 4g) remains level and nearly uniform, it provides the balanced and unbiased selection pressure needed to navigate smoothly through sparse constraints. It avoids the massive, destructive bit-switching typical of U-shaped distributions, which would immediately violate the problem’s constraints. In short, these empirical results confirm that chaotic dynamics is not a universal "plug and play" solution; the map-specific probability distribution must be structurally combined with continuous operators to safely navigate highly constrained geometries.

**Figure 8:**
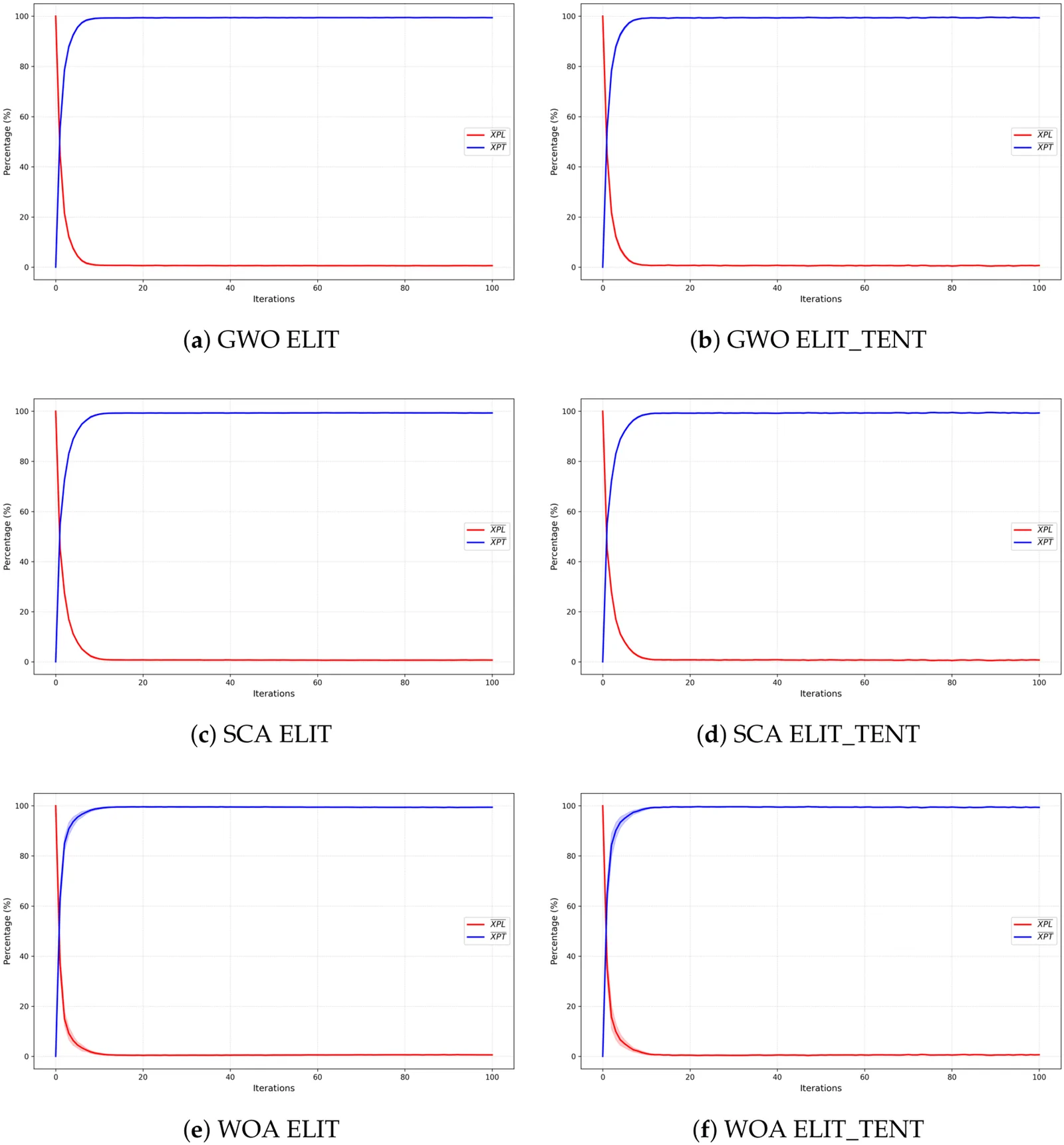
Exploration and exploitation dynamic for all metaheuristics on the SCP 51 instance.

This algorithm-dependent convergence behavior is directly mirrored by the internal phase mechanics, where the structural nature of the Elitist binarization rule unifies the swarm behaviors. To examine these underlying dynamics, the temporal exploration (XPL) and exploitation (XPT) percentages are monitored on the representative 51 instance. As plotted in Figure 8, all six algorithmic configurations exhibit a highly aggressive and nearly instantaneous phase collapse regardless of the underlying standard binarization or chaotic driver. Global exploration plummets immediately and intersects the rising exploitation curve within the first two iterations so that the swarm devotes essentially 100% of its computational effort to pure intensification by iteration 10.

Fundamentally, this uniform phase distribution highlights a recurring methodological limitation of macrospatial exploration and exploitation metrics when applied to restricted, sparse, and high-dimensional domains. While the high density of overlapping data points across the 31 independent runs confirms algorithmic stability within the narrow scatter band, the complete absence of visual variance demonstrates that the percentage metric cannot capture the internal dynamics observed during convergence tracking. Consequently, the macro percentage indicator becomes numerically blind to critical micro-oscillations and localized corrections of path steps driven by chaotic sequences. This behavior reinforces the conclusion that standard phase metrics do not provide the fine resolution needed to characterize chaotic binarization dynamics in cover landscapes, directly reflecting the analytical limitations previously identified in the KP environment.

To mathematically validate the observed optimization performance in the sparse-coverage landscape, the final fitness values are subjected to non-parametric global arbitration. The Friedman test is executed across all nine Beasley instances under the null hypothesis ($H_{0}$) that the performance distributions of the evaluated elitist configurations are statistically equal, against the alternative hypothesis ($H_{1}$) that at least one binarization scheme generates a distinct global ranking distribution. The computational validation yields a final *p*-value of $6.3176\times 10^{-2}$, which is strictly greater than the standard significance level (α=0.05). Consequently, the null hypothesis cannot be rejected at the 95% confidence level, mathematically confirming that substituting uniform random number generators with chaotic sequences does not introduce a generalized variance shift across the broader set of instances.

This statistical outcome is reported via the ranking distributions detailed in Table 9. The green color in the Table indicates that one configuration obtained the first position after applying the Friedman test. The orange color indicates that more than one configuration obtained the first position. The matrix is strongly characterized by tied-ranking positions because multiple algorithms readily converge to identical optimal fitness plateaus under the rigid selective pressure of the Elitist rule. While the WOA_SINU variant secures the best average ranking of 5.00, closely followed by the native GWO_ELIT baseline at 5.11, these minimal variations lack global mathematical significance. Rather than indicating an experimental failure, this statistical tie is a direct empirical manifestation of the No Free Lunch theorem in discrete interfaces. While microspatial tracking confirmed that targeted chaotic maps accelerate convergence velocity in specific scenarios, the sparse-density constraints ultimately force all configurations to converge to similar stagnation thresholds across the broader problem domain, demonstrating that chaos acts as a highly specialized neighborhood refiner rather than a universally dominant enhancement.

### 6.3. Evaluation on Symmetrical Flat Landscapes: The Unicost Set Covering Problem

The cascade evaluation concludes with the USCP. Table 10 unifies the final RPD metrics for the GWO, SCA, and WOA frameworks. The green color in the table indicates that a metaheuristic configuration achieved an average RPD of 0.0% across 31 independent runs of a single instance; that is, all runs reached the known optimum. Conversely, the orange color indicates that a metaheuristic configuration achieved the lowest average RPD in a single instance. Additionally, Table A7, Table A8 and Table A9 show the performance of the algorithms in greater detail.

In the GWO environment, the ELIT rule family demonstrates better average RPD, with the ELIT_SINGER variant standing out with an overall performance of 7.67. Conversely, this hierarchy changes completely in SCA and WOA, where the COM family emerges as the main discretization framework. For SCA, the best RPD is achieved by COM_LOG with 7.62, and for WOA, the best RPD is achieved by COM_PIECE with 7.03.

The unified performance distribution confirms that the STD family consistently underperforms across all three metaheuristics because it fails to provide the necessary selection pressure to escape the extensive flat optimal basins. The empirical evidence proves that navigating mathematical degeneracy requires a tailored match between the base continuous operator and the binary interface rules. While the strict selection pressure of the ELIT family is required to guide the hierarchical updates in GWO, the deterministic inversion mechanics of the COM family provide the necessary diversification of space to prevent the premature structural freezing of both SCA and WOA. Consequently, to maintain maximum search precision and avoid analytical redundancy, the COM family is selected as the definitive foundational framework governing all subsequent microspatial tracking loops and statistical arbitrations within this flat optimization landscape.

The mathematical necessity of the COM rule in this environment is directly linked to the threshold distributions demonstrated in Section 5.1. Because the USCP lacks an information–cost gradient, search agents are easily trapped in flat, symmetric basins and require severe structural perturbations to escape. Under the COM formulation ($c_{k}\leq T\left(X_{i,j}^{t+1}\right)\to ∼X_{i,j}^{bin}$), modifying the threshold distribution dynamically alters the bit inversion frequency. Using a flat, uniform distribution does not provide the necessary momentum to break algorithmic symmetry. However, replacing it with chaotic distributions mathematically alters this dynamic. Maps that heavily skew the threshold values inject the necessary target structural noise to continually force the swarm out of zero-gradient plateaus, generating search momentum that uniform randomness cannot sustain.

**Figure 9:**
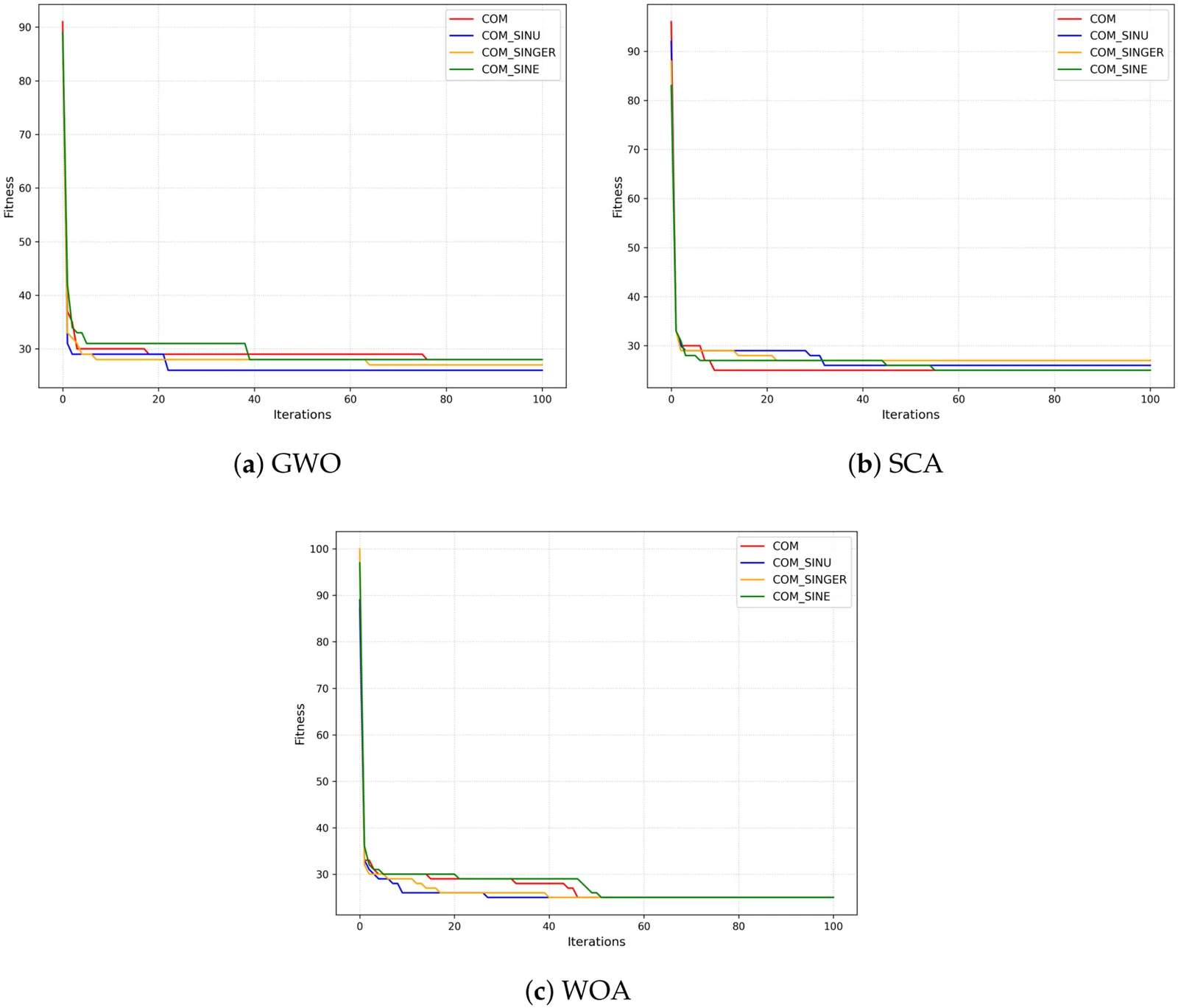
Convergence trajectories for the USCP instance *uclr10*.

To complement the operational performance metrics reported in the RPD tables, it is essential to evaluate convergence speed to understand the search dynamics of the algorithms. In the representative uclr10 instance (Figure 9), the dynamic behaviors reveal a distinct descent mechanism in each MH. For GWO, the COM_SINU variant shows early separation from the baseline and consistently maintains a lower column count trajectory throughout the search. Similarly, in SCA and WOA, the COM_SINGER and COM_SINE sequences exhibit superior plateau navigation. While the uniform baseline variants plateau relatively early, the chaotic maps allow the algorithms to perform secondary and tertiary descents in fitness, effectively locating deeper suboptimal basins in the symmetric search space.

The synthesis of global convergence behavior definitively answers how fitness evolves under complement binarization. Unlike the elitist rule, which triggers an instantaneous and permanent flat line in zero-gradient topologies, the COM framework induces a continuous cycle of binary destruction, manifesting as a recognizable stepped descent. However, the uniform stochastic baseline is prone to early stalling because its uniform distribution quickly exhausts its productive search directions, leading to stalls at suboptimal costs. In stark contrast, the specific probability density of chaotic drivers mathematically delays this stalling. For example, the heavy accumulation of values near the extremes in the sine map (see Figure 4d) generates alternating bursts of algorithmic behavior: near-zero values trigger coordinate inversions that inject massive structural noise to escape flat basins, while near-one values force variables to zero, aggressively reducing the cardinality of the active subset. This specific distribution mechanism provides the exact mathematical disruption needed to perform secondary and tertiary fitness downgrades, ultimately ensuring solid structural victories over purely memoryless uniform generators.

This transition in convergence paths is directly correlated with the internal search dynamics of the algorithms, which can be analyzed in detail by balancing the exploration ($\bar{XPL}$) and exploitation ($\bar{XPT}$) phases. Empirical validation focuses exclusively on the GWO algorithm to elucidate how the problem’s dimensionality affects the operational resolution of these traditional curves. By isolating this metaheuristic, we can contrast the behavioral mechanics between large-scale and small-scale search spaces without data redundancy. The remaining figures are shown in Appendix A.

**Figure 10:**
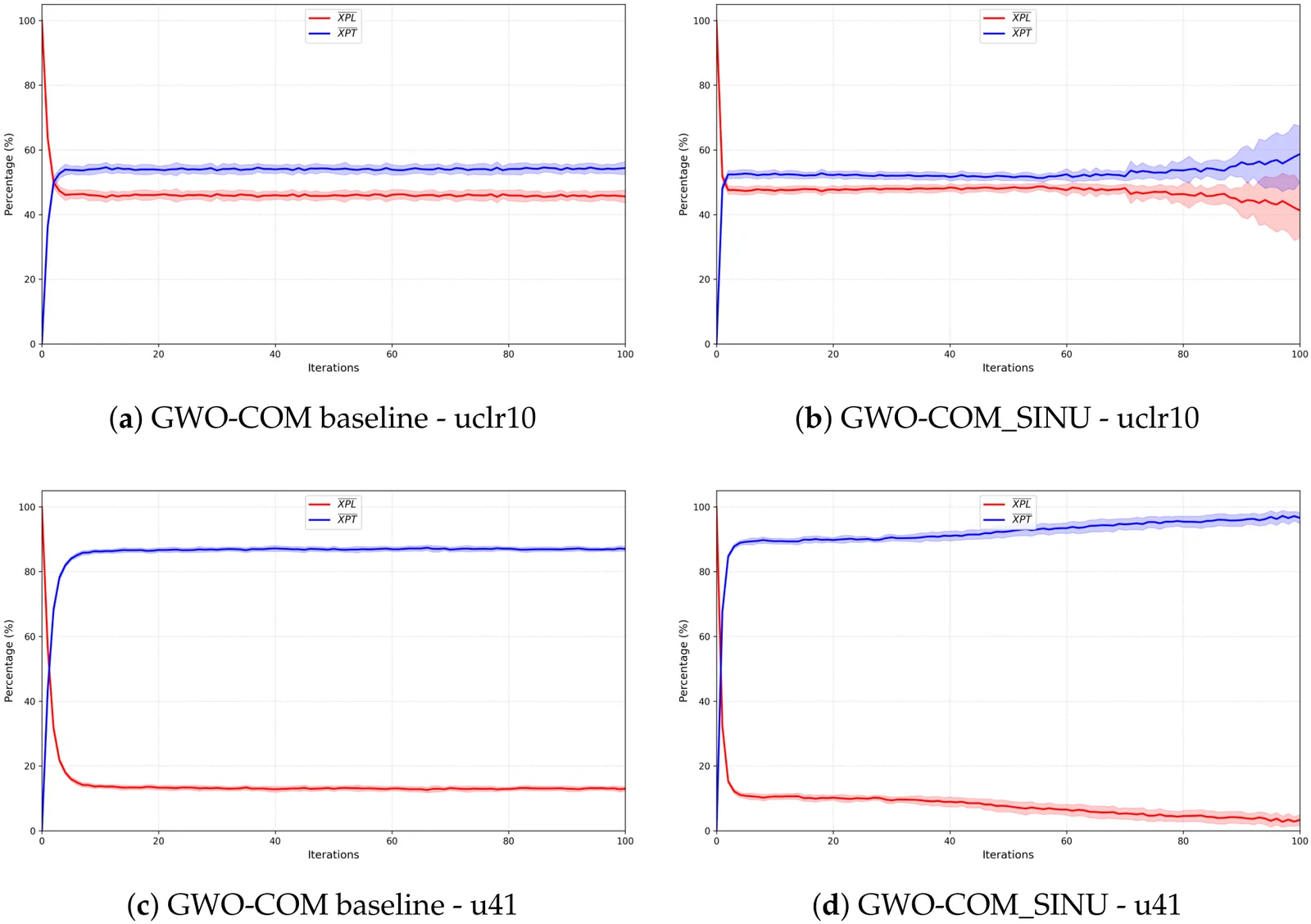
Exploration and exploitation dynamics for GWO on the uclr10 and u41 instances.

The trajectories for the low-dimensional instance *uclr10* with 210 decision variables exhibit an optimal algorithmic balance. As shown in Figure 10a, the core GWO operators sustain a steady transition where exploration phases remain near the 48% margin during the entire optimization timeline. This steady horizontal trend proves that the algorithm avoids premature convergence in low-scale landscapes because the population retains sufficient spatial diversity to continue scanning the search space effectively.

The most relevant methodological finding emerges when contrasting these results against the high-dimensional instance *u41* featuring 2000 decision variables. In this complex landscape, the spatial metric drops abruptly within the first five iterations, which forces the system into a rigid exploitation state around 80%, as seen in Figure 10c. This sudden drop occurs because traditional diversity metrics measure distance between positions in the decision space. When the number of variables grows to 2000, the population disperses through a massive landscape, which quickly saturates the standard mathematical formula.

Furthermore, these plots illustrate for the first time the statistical dispersion across different computational runs through the rendering of variance bands. In the low-dimensional environment (*uclr10*), the variance bands expand towards the final iterations, especially under chaotic modifications like the Sinusoidal map. This expanding band indicates that chaotic injection successfully shifts the search trajectories across different executions. Conversely, the high-dimensional instance (*u41*) exhibits narrow variance bands, which confirms that large-scale spaces restrict algorithmic flexibility and push all initialization runs toward the same rigid exploitation pattern.

To validate these macrospatial phase observations and confirm the global statistical significance of the final fitness distributions, a non-parametric statistical arbitration is conducted via the Friedman test. Under the null hypothesis ($H_{0}$), all complement configurations share identical median ranks across the problem instances, while the alternative hypothesis ($H_{1}$) asserts that at least one search engine exhibits structurally distinct optimization performance. The computational validation yields an extremely low *p*-value of $8.0030\times 10^{-10}$, which decisively rejects the null hypothesis and confirms that statistically significant performance differences exist among the evaluated binarization schemes. Table 11 details the ranking obtained after applying the Friedman test. The green color in the Table indicates that one configuration obtained the first position after applying the Friedman test. The orange color indicates that more than one configuration obtained the first position. As detailed in Table 11, the rank distribution contains multiple tied positions because unitary column costs force identical integer solutions within the discrete fitness function. Despite these local ties, specific chaotic variations avoid lower-tier assignments, allowing the WOA_SINGER and WOA_SINE configurations to secure the most robust average rankings of 4.59 and 4.86, respectively.

Pairwise arbitration is completed via the directional Mann–Whitney U test at a significance level of α=0.05 to uncover direct stochastic dominance. As outlined in the win matrix of Table 12, the chaotic injections are mathematically transformative within the susceptible GWO engine because the GWO_SINU and GWO_SINGER configurations achieve absolute statistical dominance by defeating the uniform stochastic GWO_COM baseline in up to 11 instances.

Conversely, the SCA and WOA frameworks reveal high structural resilience where the native stochastic baselines frequently match their chaotic variations on flat topologies. Crucially, the pairwise matrix uncovers an indisputable global framework hierarchy where virtually all SCA and WOA configurations assert overwhelming statistical superiority over the GWO suite by securing consistent victories across 10 out of 11 instances. Ultimately, this comprehensive statistical validation confirms that while chaos acts as a mandatory lifeline to prevent premature stagnation in vulnerable continuous frameworks, it successfully amplifies micro-exploitation mechanics in robust architectures to maximize overall global supremacy in the USCP landscape.

This statistical dominance of chaotic variants over uniform baselines in the USCP environment is fundamentally explained by the probability distributions visualized in Figure 4. In this degenerate landscape, uniform randomness (U(0,1)) provides a symmetric, memoryless thresholding process that fails to disrupt the stable, sub-optimal binary configurations favored by the metaheuristics. Conversely, chaotic drivers such as the Singer and Sinusoidal maps exhibit non-uniform distributions with sharp peaks and extended tails. When coupled with the Complement (COM) binarization rule, these skewed probability mass distributions generate non-periodic bursts of “destabilizing noise.” These bursts act as an essential topological disrupter, effectively forcing the search agents to oscillate out of the extensive, zero-gradient flat plateaus and toward higher-quality regions of the hypercube that uniform pseudo-random sequences cannot reach.

### 6.4. Summary and Key Findings

Multidimensional analysis across the three optimization domains provides clear architectural and methodological insights into the behavior of the binarization framework. For the 0–1 Knapsack pProblem (KP), the Standard (STD) binarization family is well-suited to capacity-constrained environments because it maintains a well-distributed coordinate search space. Within this domain, the integration of chaotic dynamics significantly improves solution quality, and the STD_CIRCLE configuration systematically minimizes the relative percentage deviation on scaled instances. This performance enhancement is observed to align with the unique, non-polarized probability mass distribution of the Circle map, which concentrates its sequences tightly between the [0.2,0.5] interval (as structurally analyzed in Figure 4h). Under the standard thresholding rule ($c_{k}\leq T\left(X_{i,j}^{t+1}\right)$), this bounded behavior appears to exert a moderately permissive selection pressure, promoting a constant, granular rate of coordinate updates and trajectory refinements near the maximum capacity limit (*W*) without triggering the massive, simultaneous bit-flipping characteristic of U-shaped distributions, which frequently pushes high-quality boundary combinations into the penalized infeasible domain. However, the standardized macrospatial exploration–exploitation curves do not accurately reflect internal exploration behavior due to instantaneous dimensional normalization, which renders the metric insensitive to localized particle modifications. Finally, non-parametric tests conclusively confirm that replacing uniform pseudo-random numbers with the Circle map produces statistically superior results in the 0–1 Knapsack Problem.

Regarding the Set Cover Problem (SCP), the Elitist (ELIT) family is the most relevant, as its high selection pressure prevents the accidental activation of stray variables. Incorporating chaos improves performance in specific real-time tracking tasks, with the ELIT_SINU variant being the most relevant. The empirical success of these chaotic maps correlates with their highly asymmetric probability distributions, such as the Sinusoidal map concentrating its mass heavily between [0.5,0.9] (Figure 4f), which statistically favors the false “otherwise” state ($X^{bin}=0$) much more frequently under the Elitist rule. This distinctive asymmetry is observed to operate as an effective column-pruning mechanism, reducing redundant column entries (1→0) to minimize total costs while safeguarding the single-column row coverages necessary to preserve feasibility. As for the internal dynamics, traditional percent-phase plots show a flat distribution that completely obscures the swarm’s micro-oscillations near the rigid constraint boundaries. This behavior is further clarified by statistical analysis: global arbitration fails to reject the null hypothesis, confirming that the characteristics of this sparse problem force all high-performance configurations into similar stagnation ranges, an empirical manifestation of the No Free Lunch theorem.

For the Unicost Set Cover Problem (USCP), the high-performance scheme is strictly algorithm-dependent, as GWO requires the selective pressure of the ELIT family, while SCA and WOA demand the diversification offered by Complement (COM). In this symmetric landscape, chaotic sequences significantly accelerate the initial optimization speed and delay premature freezing, consistently exceeding or matching the bounds of uniform random sequences. This capacity to navigate flat landscapes is empirically associated with the extreme probability mass of U-shaped chaotic drivers, which inject non-periodic, high-frequency bursts of deterministic noise directly into the complement bit-flipping loop. This structural disruption appears to destabilize the spatial equilibrium of the swarm across extensive zero-variation plateaus where uniform column costs ($c_{j}=1$) eliminate informative gradients, enabling agents to actively sample alternative regions of the hypercube. As in the previous problem, indicators of macrospatial diversification obscure the inner-neighborhood search paths and simultaneously demonstrate how high-dimensional scaling completely saturates standard positional formulas. However, global and peer statistical evaluations categorically reject the null hypothesis, confirming a decisive cross-sectional hierarchy in which SCA and WOA, leveraging specific chaotic distributions, statistically dominate the GWO set.

To contextualize the practical competitiveness of the proposed chaotic framework, it is essential to distinguish it from the latest generation of native discrete optimization algorithms. By leaving native continuous search engines intact and introducing only the chaotic binarization framework, it preserves the algorithmic flexibility of swarms. This non-invasive paradigm maintains the robust performance of metaheuristics in continuous spaces, offering a scalable alternative for NP-hard discrete industrial applications where complete algebraic redesigns are structurally impractical.

Despite these advantages, it is necessary to explicitly state the limitations of the proposed chaotic binarization framework. First, since the framework functions as an outer wrapper, its effectiveness is structurally limited by the optimization mechanics of the reference continuous solver; if the underlying continuous architecture is stuck, the chaotic layer cannot fully overcome this exploratory deficit. Furthermore, due to the pre-calculated nature of the chaotic sequence, allocated beforehand to preserve zero runtime overhead, the framework is currently unsuitable for dynamic or time-varying optimization environments where constraints change online, as real-time matrix reassignments would violate the static temporal alignment of the chaotic flows.

Finally, it is crucial to define the scope of this research. While the framework demonstrates clear advantages in preserving native swarm behavior for discrete optimization, direct experimental benchmarking with more advanced native discrete optimization solvers is beyond the scope of this study. This work focuses on validating the continuous-discrete interface mapping, rather than conducting a comparative study with purely discrete algorithms. Therefore, future research should consider direct benchmarking with native discrete techniques to quantify the specific performance trade-offs between binarized architectures and specialized native discrete architectures.

## 7. Conclusions

This research evaluated the operational limits and adaptability of a chaotic binarization framework across three distinct combinatorial optimization paradigms, providing empirical evidence on how continuous-to-discrete interface mappings behave within specific search-space topologies. By implementing a modular cascade validation across capacity-constrained (KP), sparse-coverage (SCP), and mathematically degenerate flat landscapes (USCP), the immediate experimental observations suggest that the effectiveness of the binarization rules is driven by the geometric constraints of the problem class. Within the scope of the investigated archetypes, the Standard family, coupled with the Circle map (STD_CIRCLE), appears to be well-suited for capacity-bounded over-saturation filters such as the 0–1 Knapsack Problem, as it enforces smooth coordinate updates near strict-feasibility boundaries. Conversely, the Elitist family integrated with the Sinusoidal map (ELIT_SINU) optimizes variable pruning in sparse coverage matrices such as the Set Covering Problem by aggressively deactivating redundant columns without destroying row coverage, while the Complement family provides the necessary non-periodic disruptions to combat convergence stagnation over zero-gradient plateaus in uniform-cost environments such as the Unicost Set Covering Problem.

Beyond these immediate numerical trends, the primary scientific contribution of this study lies in validating a non-invasive, zero-overhead wrapper architecture that operates in constant time O(1). This design successfully demonstrates that standard continuous swarms can navigate highly restricted discrete domains without modifying their underlying algebraic equations, thereby fully preserving their native collective kinematics. Furthermore, this research establishes an emergent taxonomic principle: chaotic binarization efficiency is governed by the structural alignment between chaotic distribution and the features of the discrete landscape. Methodologically, this study exposes a systemic limitation in the resolution of traditional macrospatial exploration–exploitation metrics when applied to highly dimensional discrete domains. The rapid convergence of algorithms causes spatial diversity to fall quickly, preventing conventional exploration–exploitation formulas from prematurely registering absolute exploitation, making metrics blind to the fine-grained micro-oscillations that drive chaotic performance variations over time.

To extend the operational boundaries of these findings, future research could be developed along three specific paths. First, the incorporation of an intelligent runtime selection mechanism for chaotic maps, guided by information from the search space, would allow the system to dynamically modify the chaotic map used and thus adjust to the conditions of the search space. Second, the architecture could be redesigned to support dynamic, time-varying combinatorial environments by replacing the precomputed chaotic map with a sliding-window allocation engine that updates chaotic flows in real time without compromising execution efficiency. Finally, the proposed discrete wrapper could be scaled up and applied to other combinatorial optimization problems. Specifically, automated machine learning pipelines could be incorporated to solve complex, high-dimensional feature selection challenges, focusing specifically on optimizing nonlinear coordinate reductions for physiological telemetry arrays in sensor-based pattern recognition platforms.

## Figures and Tables

**Table 1:** 0–1 Knapsack Problem (KP) benchmark instances categorized by correlation morphology.

ID	Instance Name	Items (*n*)	Correlation Type	Global Optimum
1_100	knapPI_1_100_1000_1	100	Uncorrelated	9147
1_200	knapPI_1_200_1000_1	200	Uncorrelated	11,238
1_500	knapPI_1_500_1000_1	500	Uncorrelated	28,857
1_1000	knapPI_1_1000_1000_1	1000	Uncorrelated	54,503
1_2000	knapPI_1_2000_1000_1	2000	Uncorrelated	110,625
2_100	knapPI_2_100_1000_1	100	Weakly Correlated	1514
2_200	knapPI_2_200_1000_1	200	Weakly Correlated	1634
2_500	knapPI_2_500_1000_1	500	Weakly Correlated	4566
2_1000	knapPI_2_1000_1000_1	1000	Weakly Correlated	9052
2_2000	knapPI_2_2000_1000_1	2000	Weakly Correlated	18,051
3_100	knapPI_3_100_1000_1	100	Strongly Correlated	2397
3_200	knapPI_3_200_1000_1	200	Strongly Correlated	2697
3_500	knapPI_3_500_1000_1	500	Strongly Correlated	7117
3_1000	knapPI_3_1000_1000_1	1000	Strongly Correlated	14,390
3_2000	knapPI_3_2000_1000_1	2000	Strongly Correlated	28,919

**Table 2:** Selected Set Covering Problem (SCP) benchmark instances from the OR-Library.

Instance	Rows (*m*)	Columns (*n*)	Cost Range	Matrix Density (%)	Optimal Solution
41	200	1000	[1, 100]	2.00	429.0
51	200	2000	[1, 100]	2.00	253.0
61	200	1000	[1, 100]	5.00	138.0
a1	300	3000	[1, 100]	2.00	253.0
b1	300	3000	[1, 100]	5.00	69.0
c1	400	4000	[1, 100]	2.00	227.0
d1	400	4000	[1, 100]	5.00	60.0
nre1	500	5000	[1, 100]	10.00	29.0
nrf1	500	5000	[1, 100]	20.00	14.0

**Table 3:** Benchmark instance profiles for the Unicost Set Covering Problem (USCP).

Instance	Rows (*m*)	Columns (*n*)	Matrix Density (%)	Optimal Solution
u41	200	1000	2.00	38.0
u51	200	2000	2.00	34.0
u61	200	1000	5.00	21.0
ud1	400	4000	5.00	24.0
unrf1	500	5000	20.00	10.0
uclr10	511	210	12.30	***25.0***
uclr11	1023	330	12.40	***23.0***
uclr12	2047	495	12.50	***23.0***
uclr13	4095	715	12.50	***23.0***
ucyc06	240	192	2.10	***60.0***
ucyc07	672	448	0.90	***144.0***

**Table 4:** Summary of experimental parameter settings for the base metaheuristics and benchmark problems.

Algorithm/Component	Parameter	Value
Global Settings (KP)	Population Size (pop)	20
	Maximum Iterations ($Max_{iter}$)	500
	Transfer Function	$T_{S2}$ (S-shaped)
Global Settings (SCP/USCP)	Population Size (pop)	10
	Maximum Iterations ($Max_{iter}$)	100
	Transfer Function	$T_{V3}$ (V-shaped)
Execution Framework	Independent Runs	31
SCA	Constant (*a*)	2.0
	$r_{1}$ parameter	Decreases linearly from *a* to 0
	$r_{2},r_{3},r_{4}$	U(0,1)
GWO	Control parameter (*a*)	Decreases linearly from 2 to 0
	$r_{1},r_{2}$	U(0,1)
WOA	Convergence parameter (*a*)	Decreases linearly from 2 to 0
	Logarithmic spiral shape (*b*)	1.0
	Probability threshold (*p*)	U(0,1)
	r,l	U(0,1) and U(−1,1)

**Table 5:** RPD obtained for each KP instance resulting from GWO, SCA, and WOA.

GWO																
BS	1_1000	1_100	1_2000	1_200	1_500	2_1000	2_100	2_2000	2_200	2_500	3_1000	3_100	3_2000	3_200	3_500	avg
STD	2.07	0.0	5.4	0.0	0.08	0.46	0.13	1.8	0.0	0.1	2.0	0.0	4.29	0.0	0.07	1.09
STD_LOG	4.28	0.0	7.82	0.0	0.58	0.95	0.13	2.79	0.0	0.19	3.38	0.0	6.23	0.0	1.41	1.85
STD_PIECE	2.34	0.0	5.66	0.0	0.09	0.45	0.13	1.92	0.0	0.0	2.0	0.0	4.38	0.0	0.17	1.14
STD_SINE	2.91	0.0	6.61	0.0	0.34	0.67	0.13	2.23	0.0	0.0	2.24	0.0	5.22	0.0	0.76	1.41
STD_SINGER	11.8	0.0	17.47	0.0	4.4	3.02	0.13	6.42	0.0	1.08	8.13	0.0	12.37	0.0	4.11	4.6
STD_SINU	14.02	0.0	18.0	0.0	8.97	5.06	0.13	7.41	0.04	2.3	9.29	0.0	12.6	0.0	6.36	5.61
STD_TENT	2.05	0.0	5.04	0.0	0.1	0.51	0.13	1.88	0.0	0.09	1.89	0.0	3.9	0.0	0.12	1.05
STD_CIRCLE	0.8	0.0	2.31	0.0	0.02	0.45	0.13	0.71	0.06	0.32	0.71	0.04	2.02	0.0	0.0	0.5
COM	12.41	0.0	17.83	0.0	5.17	3.86	0.13	6.9	0.0	1.37	8.77	0.0	12.48	0.01	4.91	4.92
COM_LOG	13.14	0.0	17.5	0.04	6.48	4.2	0.13	7.26	0.0	1.92	9.13	0.02	12.6	0.01	5.37	5.19
COM_PIECE	12.13	0.0	17.52	0.05	5.27	3.92	0.13	7.0	0.0	1.5	8.59	0.0	12.38	0.0	4.63	4.87
COM_SINE	13.34	0.0	18.01	0.01	5.43	4.09	0.13	6.94	0.0	1.62	8.78	0.01	12.23	0.0	4.82	5.03
COM_SINGER	14.63	0.0	18.05	0.32	9.41	4.78	0.13	7.41	0.06	2.77	9.29	0.07	12.64	0.17	6.11	5.72
COM_SINU	13.88	0.0	18.35	0.05	9.55	5.14	0.13	7.31	0.23	3.42	9.45	0.0	12.45	0.1	6.43	5.77
COM_TENT	12.24	0.0	17.82	0.01	5.08	3.71	0.13	6.92	0.0	1.52	8.95	0.0	12.61	0.0	4.64	4.91
COM_CIRCLE	8.16	0.0	16.94	0.08	5.4	2.75	0.13	7.06	0.05	1.29	5.28	0.03	12.3	0.0	2.52	4.13
ELIT	14.81	2.66	17.91	2.66	9.28	5.3	0.88	7.66	0.95	3.49	9.41	3.43	12.72	2.21	5.75	6.61
ELIT_LOG	14.39	2.62	18.29	3.57	8.73	5.09	0.79	7.37	0.83	3.3	9.26	3.76	12.28	2.11	6.03	6.56
ELIT_PIECE	14.45	2.59	18.03	3.17	9.14	5.13	0.91	6.97	0.99	3.44	9.6	2.78	12.27	1.72	6.59	6.52
ELIT_SINE	14.72	2.55	17.71	3.4	9.53	5.28	0.94	7.38	0.65	3.28	9.58	3.32	12.37	2.21	6.45	6.62
ELIT_SINGER	14.45	3.04	18.38	3.2	10.06	5.08	0.85	7.43	1.22	3.59	9.47	3.52	12.61	2.19	6.16	6.75
ELIT_SINU	14.84	2.34	18.32	3.1	9.64	5.2	0.86	7.29	0.9	3.88	9.22	3.8	12.52	2.13	6.4	6.7
ELIT_TENT	14.38	2.46	18.37	3.42	10.39	5.03	1.17	7.48	1.12	3.69	9.8	3.67	12.7	2.19	6.52	6.83
ELIT_CIRCLE	14.89	2.54	18.27	3.27	10.25	5.21	0.86	7.31	0.93	3.43	9.68	4.03	12.69	1.74	6.18	6.75
**SCA**																
BS	1_1000	1_100	1_2000	1_200	1_500	2_1000	2_100	2_2000	2_200	2_500	3_1000	3_100	3_2000	3_200	3_500	avg
STD	2.83	0.0	6.24	0.0	0.29	0.69	0.13	2.15	0.0	0.16	2.44	0.0	4.85	0.0	0.66	1.36
STD_LOG	4.95	0.0	8.27	0.0	0.8	1.15	0.13	3.02	0.0	0.26	3.63	0.0	6.65	0.0	1.41	2.02
STD_PIECE	3.3	0.0	6.49	0.0	0.23	0.7	0.13	2.18	0.0	0.12	2.5	0.0	4.95	0.0	0.82	1.43
STD_SINE	3.32	0.0	7.26	0.0	0.52	0.71	0.13	2.32	0.0	0.0	2.8	0.0	5.57	0.0	1.28	1.59
STD_SINGER	12.13	0.0	17.82	0.0	4.82	3.39	0.13	6.86	0.0	1.27	8.36	0.0	12.63	0.0	4.23	4.78
STD_SINU	14.51	0.0	17.69	0.01	9.35	5.11	0.13	7.06	0.16	2.81	9.34	0.0	12.15	0.01	6.45	5.65
STD_TENT	2.99	0.0	5.9	0.0	0.22	0.7	0.13	2.21	0.0	0.1	2.32	0.0	4.74	0.0	0.41	1.31
STD_CIRCLE	0.82	0.0	1.99	0.0	0.01	0.42	0.13	0.62	0.0	0.25	0.73	0.04	1.89	0.0	0.0	0.46
COM	14.19	0.0	18.09	0.18	7.87	4.87	0.13	7.22	0.03	2.45	9.29	0.07	12.43	0.06	6.24	5.54
COM_LOG	14.04	0.0	18.02	0.22	8.37	4.82	0.13	7.42	0.05	2.4	9.17	0.13	12.36	0.04	5.96	5.54
COM_PIECE	14.66	0.0	18.46	0.18	8.34	4.95	0.13	7.58	0.01	2.5	9.66	0.18	12.36	0.07	6.2	5.69
COM_SINE	13.97	0.0	18.12	0.06	7.83	4.66	0.13	7.46	0.02	2.42	9.28	0.07	12.51	0.05	5.75	5.49
COM_SINGER	14.93	0.0	18.34	1.05	9.69	4.98	0.13	7.52	0.09	3.34	9.44	0.66	12.42	1.5	6.46	6.04
COM_SINU	14.41	0.0	17.93	0.06	9.82	5.12	0.8	7.3	0.37	3.76	9.38	0.0	12.41	0.12	6.37	5.86
COM_TENT	14.54	0.0	18.26	0.1	7.99	4.82	0.13	7.44	0.03	2.42	9.6	0.06	12.35	0.06	5.87	5.58
COM_CIRCLE	13.32	0.0	18.36	0.89	8.11	4.4	0.13	7.48	0.21	2.71	9.42	0.14	12.38	0.13	5.87	5.57
BS	1_1000	1_100	1_2000	1_200	1_500	2_1000	2_100	2_2000	2_200	2_500	3_1000	3_100	3_2000	3_200	3_500	avg
ELIT	14.73	3.62	18.2	3.22	9.32	5.23	0.99	7.33	1.11	3.59	9.37	3.97	12.73	1.36	6.37	6.74
ELIT_LOG	14.56	2.71	18.0	3.31	9.8	4.98	0.89	7.34	1.05	3.32	9.22	3.36	12.59	1.97	6.08	6.61
ELIT_PIECE	14.38	2.28	17.69	3.57	9.87	5.05	1.35	7.3	0.76	3.41	9.45	3.24	12.57	1.4	6.4	6.58
ELIT_SINE	13.99	2.82	17.78	3.23	9.92	5.03	1.04	7.45	0.96	3.44	9.56	3.57	12.74	2.35	6.21	6.67
ELIT_SINGER	14.33	2.65	17.9	2.54	10.36	5.04	1.03	7.61	0.99	3.35	9.22	3.03	12.48	1.55	6.13	6.55
ELIT_SINU	14.49	2.24	18.26	3.08	9.18	5.14	0.8	7.6	0.92	3.42	9.3	3.35	12.56	2.07	6.47	6.59
ELIT_TENT	14.71	3.0	17.51	3.29	9.75	5.14	1.04	7.35	1.24	3.44	9.29	2.94	12.42	1.79	6.5	6.63
ELIT_CIRCLE	13.35	2.77	18.2	3.18	9.52	4.99	1.19	7.28	1.09	3.73	9.74	3.61	12.37	1.93	6.29	6.62
**WOA**																
BS	1_1000	1_100	1_2000	1_200	1_500	2_1000	2_100	2_2000	2_200	2_500	3_1000	3_100	3_2000	3_200	3_500	avg
STD	0.28	0.0	1.58	0.0	0.0	0.02	0.13	0.45	0.0	0.01	0.42	0.0	1.04	0.0	0.0	0.26
STD_LOG	1.05	0.0	3.29	0.0	0.04	0.23	0.13	0.96	0.0	0.01	1.07	0.0	2.45	0.0	0.0	0.62
STD_PIECE	0.24	0.0	1.44	0.0	0.0	0.03	0.13	0.44	0.0	0.0	0.44	0.0	1.16	0.0	0.0	0.26
STD_SINE	0.7	0.0	2.51	0.0	0.03	0.1	0.13	0.81	0.0	0.01	0.76	0.0	1.88	0.0	0.0	0.46
STD_SINGER	3.11	0.0	6.27	0.0	0.34	0.69	0.13	2.17	0.0	0.18	2.34	0.0	4.72	0.0	0.92	1.39
STD_SINU	4.32	0.0	9.11	0.0	0.63	0.92	0.13	3.04	0.0	0.11	3.59	0.0	6.76	0.0	1.32	2.0
STD_TENT	0.24	0.0	1.37	0.0	0.01	0.02	0.13	0.46	0.0	0.0	0.54	0.0	1.09	0.0	0.0	0.26
STD_CIRCLE	0.05	0.0	0.37	0.0	0.08	0.02	0.13	0.13	0.0	0.32	0.0	0.04	0.31	0.0	0.0	0.1
COM	11.92	0.0	17.03	0.01	5.1	3.38	0.13	6.85	0.0	1.27	8.54	0.0	12.22	0.0	4.59	4.74
COM_LOG	12.68	0.0	17.76	0.02	5.94	3.87	0.13	6.95	0.0	1.63	9.08	0.0	12.79	0.0	5.08	5.06
COM_PIECE	11.98	0.0	17.26	0.02	5.03	3.38	0.13	6.75	0.0	1.44	8.44	0.0	12.45	0.0	4.39	4.75
COM_SINE	11.99	0.0	17.58	0.02	5.06	3.71	0.13	6.9	0.0	1.41	8.42	0.0	12.61	0.0	4.62	4.83
COM_SINGER	14.33	0.0	18.1	0.04	7.86	4.89	0.13	7.29	0.03	2.36	9.59	0.0	12.37	0.04	5.87	5.53
COM_SINU	13.98	0.0	18.02	0.01	8.96	5.45	0.27	7.32	0.26	2.49	9.34	0.0	12.6	0.01	6.18	5.66
COM_TENT	11.59	0.0	17.42	0.0	4.71	3.38	0.13	6.65	0.0	1.28	8.75	0.0	12.4	0.0	4.62	4.73
COM_CIRCLE	10.01	0.0	16.52	0.04	4.37	3.02	0.13	6.52	0.03	1.28	6.69	0.0	12.28	0.0	4.23	4.34
ELIT	14.75	3.19	17.83	3.0	9.31	5.08	0.95	7.33	1.04	3.58	9.77	3.49	12.4	1.48	6.11	6.62
ELIT_LOG	14.46	2.37	18.01	3.24	9.75	5.31	1.02	7.16	1.15	3.23	9.25	3.12	12.52	2.6	6.27	6.63
ELIT_PIECE	14.75	1.92	17.96	3.34	10.04	5.07	1.24	7.33	1.03	3.63	9.35	3.53	12.3	2.15	6.42	6.67
ELIT_SINE	14.78	2.42	18.11	3.55	10.0	5.11	0.98	7.19	1.02	3.67	9.59	3.66	12.51	2.04	6.35	6.73
ELIT_SINGER	14.08	2.85	18.12	2.83	10.18	5.24	1.02	7.48	1.04	3.32	9.68	3.5	12.5	1.4	6.33	6.64
ELIT_SINU	14.73	2.56	18.02	3.04	9.6	5.32	1.05	7.5	1.25	3.65	9.4	3.76	12.76	1.8	6.32	6.72
ELIT_TENT	13.74	2.96	18.23	4.05	9.54	5.1	1.2	7.44	1.04	3.4	9.28	2.92	12.31	1.43	6.51	6.61
ELIT_CIRCLE	14.59	3.1	18.24	3.99	9.85	5.02	1.08	7.12	0.79	3.57	9.34	3.81	12.57	2.55	6.37	6.8

**Table 6:** Friedman ranking of the KP instances.

MH	GWO		SCA		WOA	
	STD	CIRCLE	STD	CIRCLE	STD	CIRCLE
	Rank	Rank	Rank	Rank	Rank	Rank
1_1000	5.0	3.5	6.0	3.5	2.0	1.0
1_100	3.5	3.5	3.5	3.5	3.5	3.5
1_2000	5.0	4.0	6.0	3.0	2.0	1.0
1_200	3.5	3.5	3.5	3.5	3.5	3.5
1_500	4.5	2.0	6.0	2.0	2.0	4.5
2_1000	4.0	5.0	6.0	3.0	1.5	1.5
2_100	3.5	3.5	3.5	3.5	3.5	3.5
2_2000	5.0	4.0	6.0	3.0	2.0	1.0
2_200	3.5	3.5	3.5	3.5	3.5	3.5
2_500	2.0	5.5	3.0	4.0	1.0	5.5
3_1000	5.0	3.0	6.0	3.0	3.0	1.0
3_100	2.0	5.0	2.0	5.0	2.0	5.0
3_2000	5.0	4.0	6.0	3.0	2.0	1.0
3_200	3.5	3.5	3.5	3.5	3.5	3.5
3_500	5.0	2.5	6.0	2.5	2.5	2.5
avg	4.00	3.73	4.70	3.30	2.50	2.77

**Table 7:** Pairwise Mann–Whitney U test win matrix (α=0.05). *p*-values are adjusted using the Holm–Bonferroni method to control the family-wise error rate. Each cell (i,j) represents the number of KP instances (out of 15) where the row algorithm statistically outperformed the column algorithm.

MH	BS	GWO		SCA		WOA	
		STD	CIRCLE	STD	CIRCLE	STD	CIRCLE
GWO	STD	–	3/15	9/15	2/15	0/15	2/15
	CIRCLE	7/15	–	8/15	0/15	0/15	1/15
SCA	STD	0/15	3/15	–	2/15	0/15	2/15
	CIRCLE	7/15	6/15	8/15	–	0/15	2/15
WOA	STD	9/15	10/15	9/15	8/15	–	3/15
	CIRCLE	7/15	7/15	8/15	6/15	5/15	–

**Table 8:** RPD obtained for each SCP instance resulting from GWO, SCA, and WOA.

GWO										
BS	41	51	61	a1	b1	c1	d1	nre1	nrf1	avg
STD	1.44	6.23	3.53	2.74	2.38	4.53	3.82	0.0	1.38	2.89
STD_LOG	1.98	7.15	3.86	4.25	2.85	5.67	5.38	0.0	5.76	4.1
STD_PIECE	1.35	5.97	3.18	3.0	2.29	4.41	4.19	0.0	0.23	2.74
STD_SINE	1.98	7.43	5.17	4.72	2.95	5.85	5.32	0.0	6.68	4.46
STD_SINGER	1.84	6.66	3.23	3.16	2.29	5.34	4.95	0.0	0.0	3.05
STD_SINU	1.63	6.38	2.9	3.39	1.5	4.77	3.87	0.0	0.0	2.72
STD_TENT	1.47	5.93	3.18	2.93	2.34	4.38	3.71	0.0	0.46	2.71
STD_CIRCLE	2.47	7.8	8.67	3.37	4.82	5.56	3.55	0.11	3.92	4.47
COM	6.71	10.99	8.63	9.22	4.16	10.37	7.85	0.0	0.0	6.44
COM_LOG	6.62	11.23	9.56	9.33	3.97	10.49	8.06	0.11	0.0	6.6
COM_PIECE	6.39	10.94	9.16	9.64	3.79	10.66	7.74	0.11	0.0	6.49
COM_SINE	6.56	10.43	8.79	9.58	3.79	10.4	7.8	0.11	0.0	6.38
COM_SINGER	7.03	11.68	9.86	10.43	5.19	10.97	8.23	0.67	0.0	7.12
COM_SINU	6.52	12.76	10.64	10.43	6.22	11.6	8.87	2.11	0.0	7.68
COM_TENT	6.48	10.29	9.63	9.46	3.79	10.63	7.63	0.11	0.0	6.45
COM_CIRCLE	6.84	10.28	8.44	9.13	4.3	10.52	7.69	0.0	0.0	6.36
ELIT	1.08	5.7	3.48	1.61	2.24	2.86	2.9	0.0	0.92	2.31
ELIT_LOG	1.04	5.62	3.6	1.86	1.82	3.61	3.33	0.11	0.92	2.43
ELIT_PIECE	1.05	5.8	3.67	1.63	1.96	2.91	2.96	0.11	1.84	2.44
ELIT_SINE	1.14	5.53	4.32	1.79	2.29	3.41	2.42	0.0	2.53	2.6
ELIT_SINGER	1.52	6.08	3.41	2.38	2.15	4.58	3.12	0.0	0.69	2.66
ELIT_SINU	1.56	6.31	2.62	3.23	1.64	4.5	4.35	0.0	0.0	2.69
ELIT_TENT	1.17	5.75	3.37	1.64	2.38	2.94	2.9	0.0	1.84	2.44
ELIT_CIRCLE	1.38	6.55	5.42	1.73	3.6	3.25	3.98	0.33	4.15	3.38
**SCA**										
BS	41	51	61	a1	b1	c1	d1	nre1	nrf1	avg
STD	1.6	6.36	4.98	3.39	2.62	5.0	4.25	0.0	1.84	3.34
STD_LOG	1.83	6.68	4.91	3.84	2.38	5.27	5.11	0.0	2.07	3.57
STD_PIECE	1.63	6.6	4.98	3.66	2.71	4.87	4.78	0.0	0.92	3.35
STD_SINE	1.92	6.74	4.42	3.77	2.43	5.22	4.19	0.22	3.46	3.6
STD_SINGER	2.06	7.08	3.55	3.58	2.76	5.98	4.35	0.0	0.0	3.26
STD_SINU	2.19	7.11	3.81	4.16	2.34	5.6	5.22	0.0	0.0	3.38
STD_TENT	1.7	6.29	5.24	3.39	2.2	4.96	4.46	0.0	2.07	3.37
STD_CIRCLE	2.3	7.03	6.66	3.88	3.65	5.07	4.89	0.11	3.69	4.14
COM	4.02	10.49	8.39	7.99	5.94	8.84	9.09	0.44	0.23	6.16
COM_LOG	4.04	10.84	8.81	7.48	5.42	8.67	8.66	0.67	0.23	6.09
COM_PIECE	4.06	10.43	8.98	8.12	5.38	9.07	9.35	0.89	0.46	6.3
COM_SINE	4.32	10.7	8.63	8.52	5.66	8.8	8.71	0.78	0.0	6.24
COM_SINGER	4.16	10.49	7.9	8.12	6.12	9.28	9.78	1.33	0.46	6.4
COM_SINU	4.11	10.37	7.57	8.11	5.94	8.95	9.78	1.0	0.23	6.23
COM_TENT	4.05	10.37	7.97	8.52	5.94	8.65	8.23	0.89	0.23	6.09
COM_CIRCLE	4.29	10.71	7.69	7.97	5.56	9.12	8.55	0.67	0.0	6.06
ELIT	1.17	5.83	4.3	1.68	1.96	3.11	2.74	0.0	1.15	2.44
ELIT_LOG	1.13	5.67	3.72	1.91	1.5	3.57	2.9	0.22	0.92	2.39
ELIT_PIECE	1.26	5.69	4.25	1.73	2.57	3.06	2.96	0.11	2.53	2.68
ELIT_SINE	1.29	5.75	4.49	1.9	1.59	3.41	2.9	0.22	1.61	2.57
ELIT_SINGER	1.56	5.84	4.0	2.77	2.2	4.72	3.6	0.11	1.38	2.91
ELIT_SINU	1.72	6.27	3.67	3.33	1.5	4.82	3.98	0.0	0.23	2.84
ELIT_TENT	1.17	5.53	4.07	1.68	1.78	2.97	3.01	0.0	1.15	2.37
ELIT_CIRCLE	1.22	6.15	4.6	1.82	3.37	3.27	3.06	0.33	3.0	2.98
BS	41	51	61	a1	b1	c1	d1	nre1	nrf1	avg
STD	1.42	6.06	4.84	2.21	3.09	3.84	3.66	0.44	3.23	3.2
STD_LOG	1.62	6.49	4.18	3.15	2.52	4.82	3.92	0.0	3.23	3.33
STD_PIECE	1.21	6.34	4.89	2.44	3.27	4.11	4.03	0.22	2.76	3.25
STD_SINE	1.7	6.25	4.91	3.32	3.27	4.55	4.35	0.33	4.38	3.67
STD_SINGER	1.8	6.43	3.48	3.06	2.48	5.37	4.14	0.22	1.61	3.18
STD_SINU	1.6	6.29	3.39	3.19	2.15	4.66	3.82	0.0	0.23	2.81
STD_TENT	1.41	6.04	4.79	2.37	3.13	3.94	3.92	0.44	3.46	3.28
STD_CIRCLE	3.62	9.05	7.18	3.14	4.96	4.86	6.72	1.56	7.14	5.36
COM	3.81	10.4	7.57	7.5	4.77	8.73	6.88	0.44	0.23	5.59
COM_LOG	4.16	9.51	7.9	6.77	5.05	8.38	7.53	0.11	0.0	5.49
COM_PIECE	4.13	10.0	7.62	7.89	5.05	8.21	7.37	0.11	0.46	5.65
COM_SINE	3.8	9.98	7.76	7.17	5.05	8.61	7.26	0.0	0.23	5.54
COM_SINGER	4.03	10.46	8.06	7.46	4.35	8.48	7.74	0.33	0.23	5.68
COM_SINU	4.56	10.35	8.37	8.15	5.42	8.98	7.85	1.33	0.23	6.14
COM_TENT	3.79	9.79	7.71	7.59	5.1	8.24	7.1	0.0	0.46	5.53
COM_CIRCLE	4.02	9.7	5.8	7.2	5.56	8.47	7.2	0.33	0.0	5.36
ELIT	1.26	5.62	4.7	1.61	2.66	3.04	3.6	0.44	2.07	2.78
ELIT_LOG	1.03	5.81	4.21	1.77	2.43	3.21	2.69	0.11	1.38	2.52
ELIT_PIECE	1.32	6.04	3.72	1.79	2.81	3.07	3.66	0.11	2.76	2.81
ELIT_SINE	1.2	5.87	4.3	1.8	2.52	3.11	3.92	0.22	3.0	2.88
ELIT_SINGER	0.94	6.04	3.65	1.8	2.57	3.34	3.17	0.22	2.07	2.64
ELIT_SINU	1.02	5.8	3.06	1.77	1.31	3.27	3.39	0.0	0.46	2.23
ELIT_TENT	1.38	5.83	4.16	1.67	2.1	3.17	3.55	0.33	2.76	2.77
ELIT_CIRCLE	2.24	8.02	6.99	2.45	5.19	3.6	5.59	1.89	6.22	4.69

**Table 9:** Friedman ranking of the SCP instances.

Inst.	GWO				SCA				WOA			
	ELIT	TENT	SINU	LOG	ELT	TENT	SINU	LOG	ELIT	TENT	SINU	LOG
	Rank	Rank	Rank	Rank	Rank	Rank	Rank	Rank	Rank	Rank	Rank	Rank
41	5.5	5.5	11.5	5.5	5.5	5.5	11.5	5.5	5.5	5.5	5.5	5.5
51	3.0	3.0	11.5	8.0	8.0	8.0	11.5	3.0	3.0	8.0	8.0	3.0
61	1.0	6.0	3.0	6.0	10.5	10.5	3.0	6.0	10.5	8.0	3.0	10.5
a1	4.5	4.5	11.5	9.5	4.5	4.5	11.5	9.5	4.5	4.5	4.5	4.5
b1	9.0	9.0	3.5	9.0	3.5	9.0	3.5	3.5	9.0	9.0	1.0	9.0
c1	4.5	4.5	11.0	9.5	4.5	4.5	12.0	9.5	4.5	4.5	4.5	4.5
d1	5.5	5.5	11.5	5.5	5.5	5.5	11.5	5.5	5.5	5.5	5.5	5.5
nre1	6.5	6.5	6.5	6.5	6.5	6.5	6.5	6.5	6.5	6.5	6.5	6.5
nrf1	6.5	6.5	6.5	6.5	6.5	6.5	6.5	6.5	6.5	6.5	6.5	6.5
avg	5.11	5.67	8.50	7.33	6.11	6.72	8.61	6.17	6.17	6.44	5.00	6.17

**Table 10:** RPD obtained for each USCP instance resulting from GWO, SCA, and WOA.

GWO												
BS	uclr12	ucyc07	ucyc06	uclr13	u61	uclr10	u51	uclr11	ud1	unrf1	u41	avg
STD	47.97	27.17	15.81	52.88	26.88	30.19	33.21	40.95	37.77	15.81	28.69	32.48
STD_LOG	57.22	33.87	24.62	63.11	41.94	37.42	53.8	51.33	50.27	20.65	49.49	43.97
STD_PIECE	47.83	27.35	15.97	51.75	26.57	28.9	34.16	41.37	38.71	17.1	29.97	32.7
STD_SINE	67.04	38.87	30.27	73.63	51.77	43.61	70.21	58.49	63.58	28.06	62.73	53.48
STD_SINGER	30.72	14.63	8.28	34.64	6.45	17.29	11.2	24.96	12.63	7.42	8.23	16.04
STD_SINU	19.35	6.41	3.06	25.95	0.0	12.77	3.7	10.1	4.17	0.0	4.33	8.17
STD_TENT	45.16	25.92	14.03	52.45	23.5	28.39	30.17	38.99	38.04	15.48	28.78	30.99
STD_CIRCLE	42.78	13.17	7.69	47.97	17.05	16.13	26.19	37.59	31.72	17.74	17.15	25.02
COM	28.89	11.02	5.75	33.24	6.3	15.74	11.1	23.28	11.42	6.77	11.21	14.97
COM_LOG	31.84	11.94	6.77	35.9	10.14	17.68	15.28	26.23	15.99	9.68	14.6	17.82
COM_PIECE	29.17	10.89	5.81	33.94	6.76	15.87	11.1	22.02	11.56	7.1	9.68	14.9
COM_SINE	32.82	12.68	6.83	37.59	10.75	17.94	17.46	26.79	18.28	9.68	16.04	18.81
COM_SINGER	22.72	9.68	3.23	30.58	2.92	13.29	7.5	17.67	7.8	0.65	7.13	11.2
COM_SINU	20.2	1.03	0.0	27.63	0.0	13.03	4.93	9.68	4.17	0.97	5.6	7.93
COM_TENT	28.89	11.04	5.54	33.94	6.14	16.0	11.2	22.58	11.42	8.06	10.61	15.04
COM_CIRCLE	29.17	10.69	5.86	33.94	6.14	16.0	11.39	22.3	9.95	5.81	10.78	14.73
ELIT	18.79	6.12	2.85	23.56	5.99	2.45	9.11	11.92	8.87	6.45	8.49	9.51
ELIT_LOG	15.43	5.89	3.01	23.98	5.07	3.61	8.92	12.06	8.87	5.16	6.96	9.0
BS	uclr12	ucyc07	ucyc06	uclr13	u61	uclr10	u51	uclr11	ud1	unrf1	u41	avg
ELIT_PIECE	16.83	6.14	3.12	24.82	5.38	3.35	8.82	9.82	9.14	6.13	7.47	9.18
ELIT_SINE	20.9	5.94	3.49	26.23	5.53	4.39	9.96	12.62	10.08	7.74	7.81	10.43
ELIT_SINGER	16.13	5.51	2.37	29.31	1.84	7.48	4.74	0.98	4.17	6.77	5.09	7.67
ELIT_SINU	23.14	5.89	1.51	33.94	1.69	14.32	4.17	11.64	4.97	6.77	4.58	10.24
ELIT_TENT	16.69	6.18	2.69	25.25	5.38	3.61	8.35	9.4	9.14	5.48	7.98	9.1
ELIT_CIRCLE	25.11	8.31	4.35	27.49	8.76	7.1	10.63	17.95	12.23	9.68	11.38	13.0
**SCA**												
BS	uclr12	ucyc07	ucyc06	uclr13	u61	uclr10	u51	uclr11	ud1	unrf1	u41	avg
STD	37.45	14.7	8.49	41.65	16.59	19.23	20.59	30.72	26.61	14.52	17.91	22.59
STD_LOG	36.04	15.46	9.73	39.97	13.82	21.03	19.26	30.43	22.31	11.94	18.59	21.69
STD_PIECE	36.75	15.03	9.41	41.51	16.59	20.52	20.3	29.45	25.27	13.87	18.93	22.51
STD_SINE	37.87	16.76	9.73	42.78	16.44	22.45	20.97	31.98	26.75	13.23	18.93	23.44
STD_SINGER	30.29	12.41	7.2	34.64	7.83	16.26	11.29	24.96	12.9	9.03	10.36	16.11
STD_SINU	28.05	10.53	5.16	31.7	4.61	14.45	8.06	20.9	9.01	5.81	7.47	13.25
STD_TENT	36.89	14.7	8.66	40.67	16.74	19.87	19.07	30.15	26.08	12.9	18.42	22.2
STD_CIRCLE	37.73	14.58	7.42	45.58	15.98	18.71	23.53	31.28	33.2	14.84	19.44	23.84
COM	15.43	0.9	0.0	25.53	2.92	9.29	4.74	8.13	5.65	5.48	7.13	7.75
COM_LOG	15.85	0.92	0.0	25.95	2.3	9.42	4.08	6.17	6.72	5.48	6.96	7.62
COM_PIECE	15.15	0.78	0.0	25.67	2.92	10.58	4.93	5.19	5.51	6.13	7.56	7.67
COM_SINE	15.15	1.55	0.0	24.82	2.92	9.68	4.84	6.73	6.05	6.13	7.13	7.73
COM_SINGER	16.83	0.43	0.0	25.67	1.69	11.35	4.17	8.42	6.32	5.81	7.39	8.01
COM_SINU	17.81	0.49	0.0	25.25	2.3	12.26	4.74	8.84	5.51	5.81	7.3	8.21
COM_TENT	16.97	0.85	0.0	25.81	3.23	9.42	5.31	7.43	5.65	7.42	7.64	8.16
COM_CIRCLE	15.01	0.74	0.0	27.49	3.38	8.65	5.98	10.1	5.65	6.77	7.39	8.29
ELIT	22.58	6.77	2.69	29.45	5.07	4.77	9.39	14.03	8.33	6.13	9.0	10.75
ELIT_LOG	22.16	6.52	2.74	28.47	4.92	6.06	9.3	15.71	8.06	5.81	7.64	10.67
ELIT_PIECE	21.18	6.63	3.12	28.05	5.22	4.77	9.01	15.57	7.66	6.45	7.81	10.5
ELIT_SINE	23.7	6.54	2.85	28.05	6.14	5.42	9.77	16.41	9.41	8.39	8.15	11.35
ELIT_SINGER	19.21	6.7	2.15	28.05	2.46	6.06	5.79	5.75	5.11	2.9	5.01	8.11
ELIT_SINU	19.64	7.01	2.85	26.23	0.92	8.65	4.65	8.84	4.44	5.16	5.01	8.49
ELIT_TENT	23.0	6.52	2.69	29.73	4.92	4.65	9.58	14.59	8.33	6.13	8.57	10.79
ELIT_CIRCLE	23.84	7.57	4.41	29.45	7.07	6.71	12.24	16.13	15.59	7.74	10.61	12.85
**WOA**												
BS	uclr12	ucyc07	ucyc06	uclr13	u61	uclr10	u51	uclr11	ud1	unrf1	u41	avg
STD	29.03	9.48	3.87	34.5	12.29	13.03	14.42	21.88	18.01	11.29	13.16	16.45
STD_LOG	30.29	10.78	4.68	36.04	11.37	13.81	14.04	24.26	17.34	10.32	12.39	16.85
STD_PIECE	29.59	9.07	4.78	34.36	12.75	12.65	15.37	23.28	19.35	11.61	13.33	16.92
STD_SINE	32.26	11.6	5.48	36.33	12.75	14.71	14.9	25.11	19.76	12.58	13.84	18.12
STD_SINGER	27.63	9.45	3.92	31.14	6.14	10.06	8.82	18.79	10.22	9.03	7.98	13.02
STD_SINU	18.65	6.52	2.69	26.09	3.53	7.48	6.83	11.64	5.91	5.48	5.94	9.16
STD_TENT	28.61	9.23	4.41	35.2	11.21	11.61	14.61	22.58	18.55	10.32	14.43	16.43
STD_CIRCLE	32.54	12.25	9.14	36.89	19.66	17.81	20.68	27.49	18.41	10.32	20.8	20.54
COM	13.32	0.16	0.0	25.25	3.38	6.32	5.03	3.37	6.99	7.42	6.62	7.08
COM_LOG	16.13	0.43	0.0	26.09	2.92	8.26	4.65	5.05	6.05	6.77	6.37	7.52
COM_PIECE	11.64	0.07	0.0	25.67	3.38	7.61	4.55	3.65	8.2	5.48	7.05	7.03
COM_SINE	12.62	0.27	0.0	23.7	3.69	5.81	4.93	4.49	6.72	6.77	6.71	6.88
COM_SINGER	15.29	0.34	0.0	25.11	1.84	8.52	4.65	3.93	5.78	5.16	6.88	7.05
COM_SINU	15.71	0.11	0.0	27.77	5.84	6.45	6.55	5.75	8.47	8.71	7.64	8.45
COM_TENT	12.9	0.16	0.0	25.11	3.38	6.19	4.36	3.09	7.26	6.77	7.3	6.96
COM_CIRCLE	13.18	0.07	0.0	26.79	4.3	6.84	5.22	4.77	6.32	7.1	7.56	7.47
ELIT	25.39	7.91	3.71	30.01	9.06	9.29	12.24	18.65	13.98	10.0	11.38	13.78
ELIT_LOG	23.7	7.28	2.74	27.35	7.53	6.19	10.25	15.15	10.75	9.35	9.76	11.82
ELIT_PIECE	26.23	7.84	3.49	30.72	7.99	6.06	11.76	20.2	13.58	9.68	11.54	13.55
ELIT_SINE	25.25	7.89	3.82	28.75	8.91	8.26	10.72	16.55	13.17	10.0	9.76	13.01
ELIT_SINGER	19.64	7.06	2.37	27.35	3.07	6.06	6.74	8.27	7.26	6.13	6.11	9.1
ELIT_SINU	16.41	5.91	2.37	26.65	2.61	5.16	6.07	5.19	6.05	4.52	5.26	7.84
ELIT_TENT	25.25	7.37	4.14	28.89	10.45	6.84	12.14	16.97	14.25	9.35	11.12	13.34
ELIT_CIRCLE	28.61	11.18	8.71	32.96	16.13	13.68	17.27	23.7	20.16	10.0	18.76	18.29

**Table 11:** Friedman ranking of the USCP instances.

Inst.	GWO				SCA				WOA			
	COM	SINU	SINGER	SINE	COM	SINU	SINGER	SINE	COM	SINU	SINGER	SINE
	Rank	Rank	Rank	Rank	Rank	Rank	Rank	Rank	Rank	Rank	Rank	Rank
u41	11.0	1.0	6.0	12.0	6.0	6.0	6.0	6.0	6.0	6.0	6.0	6.0
u51	11.0	5.5	10.0	12.0	5.5	5.5	1.0	5.5	5.5	5.5	5.5	5.5
u61	8.5	3.0	8.5	12.0	3.0	3.0	3.0	8.5	8.5	8.5	3.0	8.5
uclr10	11.0	7.5	7.5	12.0	4.0	7.5	7.5	7.5	2.0	2.0	7.5	2.0
uclr11	11.0	7.0	10.0	12.0	7.0	7.0	7.0	7.0	3.0	3.0	3.0	1.0
uclr12	11.0	9.5	9.5	12.0	3.5	7.5	7.5	3.5	3.5	3.5	3.5	3.5
uclr13	11.0	9.0	9.0	12.0	4.0	4.0	4.0	4.0	4.0	9.0	4.0	4.0
ucyc06	11.0	5.0	10.0	12.0	5.0	5.0	5.0	5.0	5.0	5.0	5.0	5.0
ucyc07	11.0	8.5	10.0	12.0	6.0	6.0	6.0	8.5	2.5	2.5	2.5	2.5
ud1	11.0	3.0	8.0	12.0	3.0	3.0	8.0	3.0	8.0	8.0	3.0	8.0
unrf1	7.5	1.5	1.5	7.5	7.5	7.5	7.5	7.5	7.5	7.5	7.5	7.5
avg	10.45	5.50	8.18	11.59	4.95	5.64	5.68	6.00	5.05	5.50	4.59	4.86

**Table 12:** Pairwise Mann–Whitney U test win matrix (α=0.05). *p*-values are adjusted using the Holm–Bonferroni method to control the family-wise error rate. Each cell (i,j) represents the number of USCP instances (out of 11) where the row algorithm statistically outperformed the column algorithm.

MH	BS	GWO				SCA				WOA			
		COM	SINE	SINGER	SINU	COM	SINE	SINGER	SINU	COM	SINE	SINGER	SINU
GWO	COM	X	11/11	0/11	0/11	0/11	0/11	0/11	0/11	0/11	0/11	0/11	0/11
	SINE	0/11	X	0/11	0/11	0/11	0/11	0/11	0/11	0/11	0/11	0/11	0/11
	SINGER	11/11	11/11	X	0/11	1/11	1/11	1/11	1/11	1/11	1/11	1/11	2/11
	SINU	11/11	11/11	9/11	X	4/11	4/11	4/11	4/11	4/11	4/11	4/11	5/11
SCA	COM	10/11	11/11	8/11	2/11	X	1/11	0/11	1/11	0/11	0/11	0/11	4/11
	SINE	10/11	11/11	8/11	3/11	0/11	X	0/11	1/11	0/11	0/11	0/11	3/11
	SINU	10/11	11/11	7/11	1/11	1/11	1/11	0/11	X	0/11	0/11	0/11	3/11
WOA	SINGER	10/11	11/11	8/11	3/11	1/11	1/11	X	0/11	0/11	1/11	0/11	3/11
	COM	10/11	10/11	7/11	4/11	3/11	3/11	3/11	3/11	X	0/11	1/11	2/11
	SINE	10/11	11/11	7/11	4/11	3/11	3/11	3/11	3/11	0/11	X	1/11	2/11
	SINGER	10/11	11/11	8/11	4/11	2/11	2/11	1/11	2/11	0/11	1/11	X	4/11
	SINU	9/11	10/11	5/11	4/11	3/11	2/11	3/11	3/11	0/11	1/11	1/11	X

**Figure A1:**
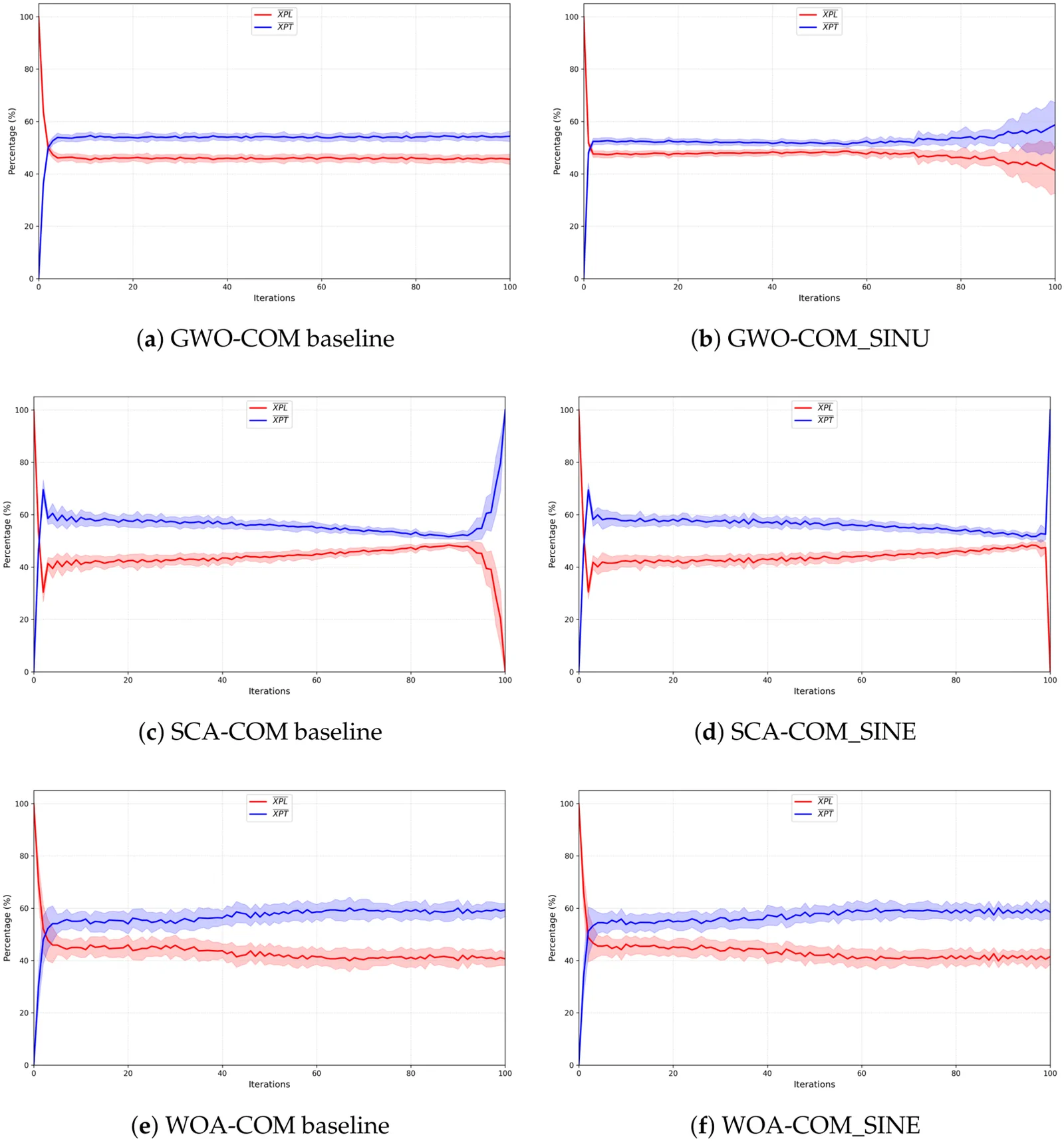
Exploration and exploitation dynamics for all metaheuristics on the *uclr10* instance.

**Figure A2:**
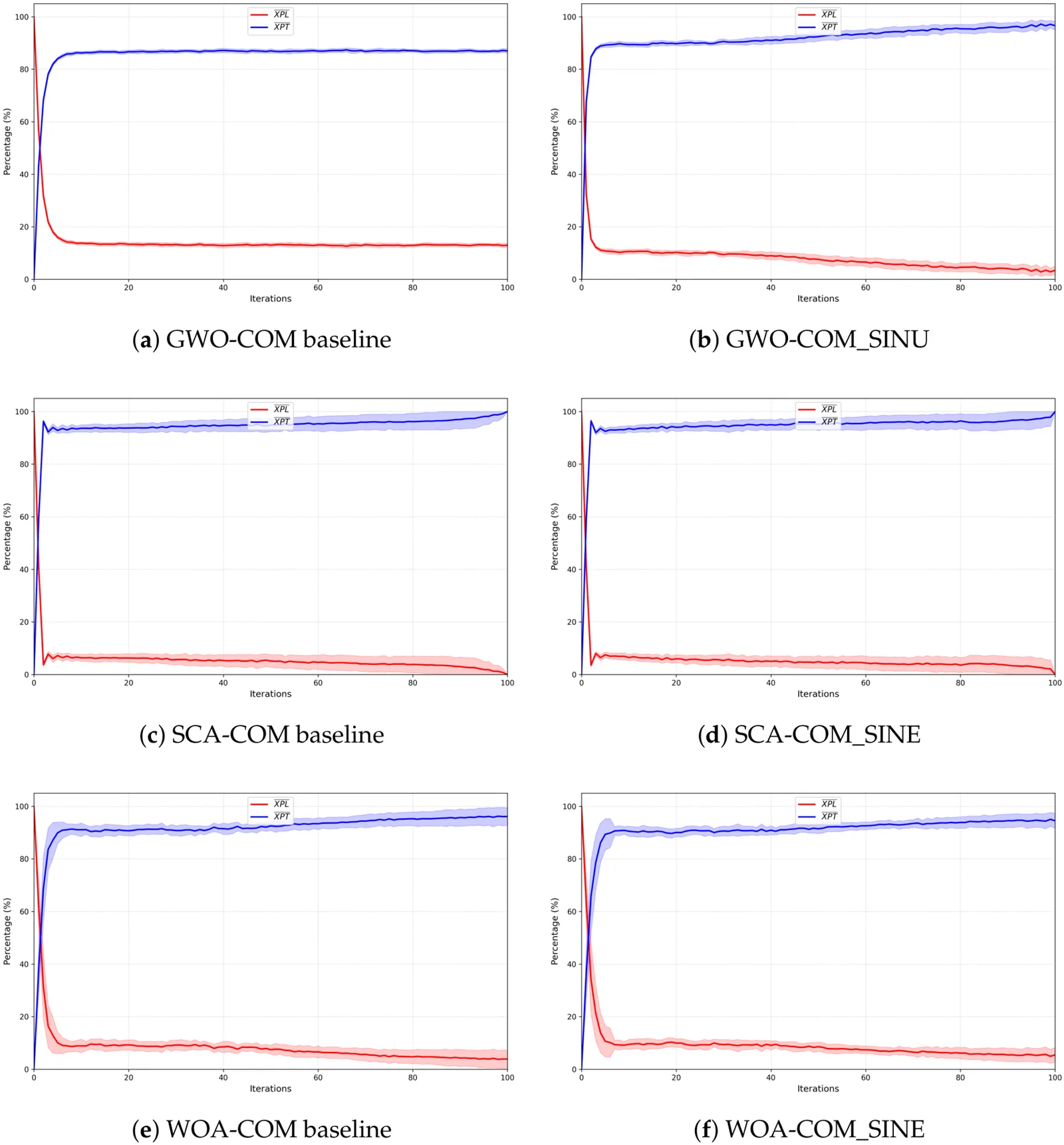
Exploration and exploitation dynamics for all metaheuristics on the *u41* instance.

## Data Availability

All code, tables, graphs, and raw data are available to you in our GitHub repository. The link is the following: https://github.com/FelipeCisternasCaneo/Chaotic-Regulation-of-Exploration-and-Exploitation.git (accessed on 29 July 2026). This repository includes the complete experimental setup, including: (i) the source code for the proposed chaotic discretization framework; (ii) the per-problem configuration parameters; (iii) the raw execution logs for all 72 configurations across the 31 independent runs; and (iv) the scripts used to generate the statistical matrices and performance comparisons presented in Section 6.

## Appendix A. Complete Exploration–Exploitation Profiles for USCP

This appendix provides the complete empirical evaluation regarding the dynamic balance between exploration ($\bar{XPL}$) and exploitation ($\bar{XPT}$) phases for all tested algorithms to ensure study reproducibility.

The extended results demonstrate behavioral consistency across runs and show how problem dimensionality influences structural diversity loss over the optimization horizon.

### Appendix A.1. The uclr10 Instance

The trajectories in Figure A1 correspond to the USCP instance *uclr10* with 210 decision variables. Under this low dimensional framework, the algorithms maintain a responsive phase transition. The incorporation of nonlinear chaotic mappings systematically prevents early diversity collapse because it sustains balanced exploration levels throughout the search process. Notable variations occur in SCA variants where the operators force a sharp exploitation convergence near the final iterations.

### Appendix A.2. The u41 Instance

Figure A2 gathers the operational profiles for the high-dimensional instance *u41*, which features 2000 decision variables. These trajectories illustrate a severe saturation phenomenon. Across all evaluated metaheuristics, the spatial diversity metrics drop abruptly within the first iterations, locking the population into a rigid exploitation state. This behavior supports the conclusion that traditional positional diversity metrics lose analytical resolution when applied to large-scale optimization landscapes.

## Appendix B. Statistical Variability Analysis

This appendix provides a comprehensive statistical variability analysis to supplement the aggregate performance results presented in the main manuscript. To assess the robustness and stability of the proposed chaotic binarization framework, the following tables report the best objective value achieved, the median performance, and the standard deviation (std) calculated over 31 independent experimental runs for the GWO, SCA, and WOA metaheuristics. The data is organized by problem domain and binarization rule: Table A1, Table A2 and Table A3 detail the results for the 0–1 Knapsack Problem (KP); Table A4, Table A5 and Table A6 present the findings for the Set Covering Problem (SCP); and Table A7, Table A8 and Table A9 contain the data for the Unicost Set Covering Problem (USCP) configurations.

**Table A1:** Statistical performance summary of the proposed chaotic binarization framework for the 0–1 Knapsack Problem (KP) instances. Columns report the known global optimum (Opt), best fitness achieved, median, and standard deviation (std) calculated over 31 independent runs for the GWO, SCA, and WOA metaheuristics under the Complement (COM) rule across different chaotic map configurations.

**GWO**																									
Instance	Opt	COM			COM_LOG			COM_PIECE			COM_SINE			COM_SINGER			COM_SINU			COM_TENT			COM_CIRCLE		
		best	median	std	best	median	std	best	median	std	best	median	std	best	median	std	best	median	std	best	median	std	best	median	std
1_1000	54,503	49,922	47,621	692.08	50,637	47,256	1031.82	49,765	47,860	576.9	49,002	47,095	632.17	49,632	46,235	1293.33	48,900	46,972	1021.38	49,271	47,873	657.4	50,789	49,908	439.6
1_100	9147	9147	9147	0.0	9147	9147	0.0	9147	9147	0.0	9147	9147	0.0	9147	9147	0.0	9147	9147	0.0	9147	9147	0.0	9147	9147	0.0
1_2000	110,625	94,380	90,580	1319.05	95,370	91,140	1781.26	95,187	90,986	1749.95	93,710	90,616	1201.91	93,587	90,600	1447.54	93,509	90,069	1361.82	95,343	90,440	1566.73	93,994	91,947	809.65
1_200	11,238	11,238	11,238	1.98	11,238	11,238	8.69	11,238	11,238	5.77	11,238	11,238	3.75	11,238	11,223	43.72	11,238	11,238	5.59	11,238	11,238	3.31	11,238	11,227	7.76
1_500	28,857	28,132	27,366	318.1	27,389	26,976	237.89	28,272	27,296	323.61	28,164	27,141	449.12	27,045	26,124	422.49	27,483	26,071	578.99	28,320	27,335	344.95	27,999	27,307	232.92
2_1000	9052	8764	8703	33.94	8826	8648	56.96	8822	8694	48.47	8797	8686	36.06	8765	8632	75.76	8699	8585	63.0	8806	8716	39.18	8869	8794	33.08
2_100	1514	1512	1512	0.0	1512	1512	0.0	1512	1512	0.0	1512	1512	0.0	1512	1512	0.0	1512	1512	0.0	1512	1512	0.0	1512	1512	0.0
2_2000	18,051	16,945	16,799	65.45	17,215	16,690	134.39	17,048	16,763	94.4	17,033	16,803	98.61	17,051	16,693	130.21	17,041	16,708	133.87	17,029	16,795	94.51	17,005	16,758	88.87
2_200	1634	1634	1634	0.0	1634	1634	0.0	1634	1634	0.0	1634	1634	0.25	1634	1633	0.3	1634	1633	3.07	1634	1634	0.25	1634	1633	0.4
2_500	4566	4554	4500	22.8	4514	4474	16.53	4520	4499	14.88	4541	4485	22.55	4503	4432	34.85	4492	4405	37.15	4528	4494	15.41	4537	4507	12.11
3_1000	14,390	13,383	13,089	106.11	13,485	13,082	131.22	13,386	13,090	120.06	13,481	13,086	113.25	13,383	13,075	165.61	13,467	13,076	149.51	13,283	13,088	86.19	13,789	13,590	66.4
3_100	2397	2397	2397	0.18	2397	2397	1.29	2397	2397	0.25	2397	2397	1.27	2397	2396	2.4	2397	2397	0.0	2397	2397	0.3	2397	2397	2.1
3_2000	28,919	25,818	25,317	210.12	25,618	25,313	230.17	26,004	25,310	281.31	25,909	25,415	249.86	25,616	25,314	202.94	26,014	25,307	218.36	26,113	25,214	278.27	25,818	25,312	220.74
3_200	2697	2697	2697	0.5	2697	2697	0.8	2697	2697	0.56	2697	2697	0.43	2697	2694	5.45	2697	2695	3.19	2697	2697	0.25	2697	2697	0.0
3_500	7117	6915	6795	55.6	6909	6716	52.3	6909	6810	58.2	6915	6800	57.67	6817	6693	84.64	6815	6683	89.15	6915	6805	62.45	7013	6917	41.58
**SCA**																									
Instance	Opt	COM			COM_LOG			COM_PIECE			COM_SINE			COM_SINGER			COM_SINU			COM_TENT			COM_CIRCLE		
		best	median	std	best	median	std	best	median	std	best	median	std	best	median	std	best	median	std	best	median	std	best	median	std
1_1000	54,503	49,110	46,696	889.22	48,051	46,965	819.06	48,216	46,484	859.93	48,435	46,811	796.95	48,477	46,294	1097.11	47,996	46,663	791.03	48,867	46,400	823.79	48,510	47,090	482.77
1_100	9147	9147	9147	0.0	9147	9147	0.0	9147	9147	0.0	9147	9147	0.0	9147	9147	0.0	9147	9147	0.0	9147	9147	0.0	9147	9147	0.0
1_2000	110,625	94,027	90,319	1462.6	95,094	90,600	1530.11	94,640	90,076	1537.12	95,557	90,457	1493.6	94,604	90,276	1565.28	94,238	90,822	1890.13	95,329	90,367	1697.37	93,304	90,247	1441.95
1_200	11,238	11,238	11,227	35.74	11,238	11,227	33.54	11,238	11,227	35.86	11,238	11,227	5.52	11,238	11,125	81.96	11,238	11,227	5.52	11,238	11,227	15.08	11,238	11,099	78.94
1_500	28,857	27,534	26,636	469.39	26,993	26,484	320.49	27,409	26,394	302.34	27,353	26,634	383.81	27,610	26,051	635.09	27,051	26,058	631.32	27,446	26,540	377.14	27,201	26,545	337.16
2_1000	9052	8718	8614	47.83	8837	8609	79.35	8712	8608	55.39	8784	8621	63.55	8759	8590	72.19	8815	8576	82.28	8769	8610	59.88	8753	8645	44.7
2_100	1514	1512	1512	0.0	1512	1512	0.0	1512	1512	0.0	1512	1512	0.0	1512	1512	0.0	1512	1501	6.37	1512	1512	0.0	1512	1512	0.0
2_2000	18,051	17,009	16,750	134.58	16,964	16,693	116.25	16,946	16,665	115.61	16,982	16,689	115.11	16,906	16,677	140.28	16,980	16,725	107.82	16,965	16,718	132.14	16,954	16,695	122.59
2_200	1634	1634	1634	0.51	1634	1633	0.44	1634	1634	0.4	1634	1634	0.44	1634	1633	1.84	1634	1627	2.4	1634	1633	0.51	1634	1633	3.14
2_500	4566	4505	4454	24.16	4499	4461	24.14	4512	4447	22.51	4517	4451	19.22	4497	4413	33.43	4459	4401	40.33	4537	4453	28.3	4507	4437	30.11
3_1000	14,390	13,388	12,989	137.91	13,384	12,989	160.74	13,276	12,983	125.46	13,383	12,990	154.91	13,490	12,987	153.1	13,377	13,073	140.98	13,390	12,984	154.52	13,287	12,988	95.71
3_100	2397	2397	2396	3.34	2397	2396	3.95	2397	2397	18.02	2397	2396	2.64	2397	2396	33.25	2397	2397	0.0	2397	2397	2.51	2397	2396	3.41
3_2000	28,919	25,908	25,315	214.13	25,917	25,311	246.47	26,211	25,308	308.62	26,014	25,316	273.28	26,314	25,218	315.92	26,004	25,313	241.45	25,705	25,314	188.07	25,714	25,315	195.95
3_200	2697	2697	2696	2.14	2697	2697	2.2	2697	2696	2.19	2697	2697	1.91	2697	2685	45.17	2697	2696	4.43	2697	2696	2.36	2697	2694	2.88
3_500	7117	6814	6691	67.88	6815	6707	72.74	6817	6617	72.64	6817	6706	70.42	6806	6689	71.73	6817	6617	95.37	6816	6705	62.97	6913	6704	73.5
**WOA**																									
Instance	Opt	COM			COM_LOG			COM_PIECE			COM_SINE			COM_SINGER			COM_SINU			COM_TENT			COM_CIRCLE		
		best	median	std	best	median	std	best	median	std	best	median	std	best	median	std	best	median	std	best	median	std	best	median	std
1_1000	54,503	49,135	47,952	591.05	48,767	47,697	558.63	49,832	47,946	637.67	49,312	47,990	563.3	50,170	46,554	1053.53	49,464	46,488	953.99	49,743	48,267	670.32	50,372	49,078	620.05
1_100	9147	9147	9147	0.0	9147	9147	0.0	9147	9147	0.0	9147	9147	0.0	9147	9147	0.0	9147	9147	0.0	9147	9147	0.0	9147	9147	0.0
1_2000	110,625	95,052	91,624	1369.53	92,635	90,947	1064.07	94,761	91,229	1265.33	94,146	90,911	1204.2	95,646	90,545	1661.75	96,128	90,753	2127.33	95,027	91,279	1269.32	95,741	91,819	1534.08
1_200	11,238	11,238	11,238	3.31	11,238	11,238	4.42	11,238	11,238	4.11	11,238	11,238	4.46	11,238	11,238	5.85	11,238	11,238	3.75	11,238	11,238	1.98	11,238	11,238	5.45
1_500	28,857	28,076	27,424	293.99	27,788	27,103	289.37	28,108	27,402	308.64	27,953	27,380	267.36	27,972	26,591	408.81	27,347	26,288	503.47	28,173	27,520	327.15	28,247	27,528	282.72
2_1000	9052	8893	8744	44.68	8826	8674	57.29	8810	8751	36.0	8790	8714	33.4	8758	8595	57.07	8830	8536	87.7	8944	8733	68.47	8879	8773	38.91
2_100	1514	1512	1512	0.0	1512	1512	0.0	1512	1512	0.0	1512	1512	0.0	1512	1512	0.0	1512	1512	4.42	1512	1512	0.0	1512	1512	0.0
2_2000	18,051	16,984	16,797	88.47	17,062	16,796	83.94	17,279	16,802	122.41	17,068	16,787	84.86	17,031	16,725	112.85	17,133	16,714	139.67	17,092	16,832	115.45	17,083	16,863	92.48
2_200	1634	1634	1634	0.0	1634	1634	0.0	1634	1634	0.0	1634	1634	0.0	1634	1634	0.51	1634	1627	3.26	1634	1634	0.0	1634	1634	1.29
2_500	4566	4555	4503	22.12	4549	4492	16.52	4529	4498	14.96	4533	4497	17.22	4513	4453	24.6	4525	4461	34.59	4555	4505	20.26	4551	4504	18.16
3_1000	14,390	13,469	13,167	115.36	13,290	13,087	91.94	13,489	13,178	112.59	13,486	13,178	109.54	13,375	12,982	135.73	13,282	13,072	122.92	13,366	13,090	99.23	13,985	13,385	156.14
3_100	2397	2397	2397	0.18	2397	2397	0.3	2397	2397	0.18	2397	2397	0.25	2397	2397	0.25	2397	2397	0.0	2397	2397	0.18	2397	2397	0.18
3_2000	28,919	25,916	25,318	233.63	25,811	25,203	257.18	25,718	25,311	207.2	25,906	25,212	237.73	25,811	25,318	233.19	26,015	25,306	268.7	25,715	25,403	223.74	25,815	25,319	215.09
3_200	2697	2697	2697	0.0	2697	2697	0.43	2697	2697	0.18	2697	2697	0.43	2697	2697	1.2	2697	2697	1.08	2697	2697	0.0	2697	2697	0.0
3_500	7117	6913	6810	54.0	6912	6717	61.15	6917	6812	49.45	6900	6805	49.56	6908	6708	75.08	6817	6706	89.14	6901	6808	44.38	6998	6813	48.43

**Table A2:** Statistical performance summary of the proposed chaotic binarization framework for the 0–1 Knapsack Problem (KP) instances. Columns report the known global optimum (Opt), best fitness achieved, median, and standard deviation (std) calculated over 31 independent runs for the GWO, SCA, and WOA metaheuristics under the Elitist (ELIT) rule across different chaotic map configurations.

**GWO**																									
Instance	Opt	ELIT			ELIT_LOG			ELIT_PIECE			ELIT_SINE			ELIT_SINGER			ELIT_SINU			ELIT_TENT			ELIT_CIRCLE		
		best	median	std	best	median	std	best	median	std	best	median	std	best	median	std	best	median	std	best	median	std	best	median	std
1_1000	54,503	49,583	46,546	1065.28	48,212	46,831	807.57	49,049	46,461	1029.28	48,975	46,292	1051.02	49,107	46,631	971.26	48,424	46,239	991.92	49,590	46,561	972.97	48,220	46,640	1189.19
1_100	9147	9147	8911	205.58	9147	8911	170.83	9147	8911	154.39	9147	8911	197.5	9147	8911	154.01	9147	8940	185.09	9147	8940	209.83	9147	8911	203.62
1_2000	110,625	94,037	90,751	1419.67	93,222	90,382	1292.5	95,236	90,374	1659.55	94,328	91,085	1763.41	92,560	90,467	1407.99	93,540	90,374	1517.64	93,337	90,089	1611.9	93,257	90,636	1250.97
1_200	11,238	11,227	10,936	201.06	11,227	10,831	187.53	11,238	10,899	210.94	11,238	10,869	233.04	11,227	10,907	239.67	11,238	10,899	236.03	11,227	10,853	249.51	11,238	10,874	212.08
1_500	28,857	27,516	26,140	592.47	27,952	26,209	831.49	27,624	26,214	679.44	27,473	26,079	522.73	27,238	26,101	704.54	26,995	25,992	539.98	27,007	25,815	640.06	26,947	25,876	533.19
2_1000	9052	8731	8570	70.81	8767	8608	87.98	8789	8598	92.5	8727	8576	82.27	8752	8574	67.79	8795	8567	79.08	8753	8590	82.65	8721	8579	78.78
2_100	1514	1512	1501	8.4	1512	1501	8.81	1512	1501	11.16	1512	1501	9.02	1512	1501	8.93	1512	1501	12.55	1512	1498	14.5	1512	1501	11.29
2_2000	18,051	16,933	16,683	114.27	16,984	16,723	150.14	17,164	16,770	141.89	17,000	16,722	129.74	16,954	16,719	141.14	17,129	16,736	145.95	17,059	16,696	133.25	16,992	16,714	126.26
2_200	1634	1634	1618	11.14	1634	1624	11.38	1633	1618	8.49	1634	1623	6.75	1634	1617	12.24	1634	1619	9.91	1634	1618	14.23	1634	1619	9.93
2_500	4566	4492	4409	43.01	4495	4417	55.24	4503	4412	40.57	4530	4412	50.94	4532	4389	56.98	4486	4392	45.92	4500	4394	50.76	4482	4409	43.63
3_1000	14,390	13,284	13,082	145.98	13,386	13,072	134.34	13,178	12,989	112.83	13,290	12,987	143.72	13,284	12,990	147.18	13,482	12,990	172.84	13,285	12,984	131.09	13,384	12,978	157.37
3_100	2397	2396	2294	42.4	2397	2294	37.61	2397	2295	50.45	2390	2295	41.33	2396	2296	40.27	2396	2293	36.92	2390	2295	40.15	2397	2293	31.74
3_2000	28,919	25,619	25,216	182.57	26,111	25,319	243.86	25,806	25,314	181.76	26,216	25,313	331.2	25,619	25,307	212.85	25,817	25,215	239.68	25,714	25,218	210.63	25,916	25,211	264.12
3_200	2697	2695	2596	49.79	2697	2656	48.8	2697	2673	48.49	2697	2597	49.17	2697	2597	48.4	2696	2649	47.15	2697	2596	50.18	2697	2684	50.85
3_500	7117	6916	6708	91.23	6812	6703	74.52	6805	6617	73.17	6811	6684	80.17	6815	6706	78.95	6889	6696	93.85	6812	6617	89.81	6814	6708	84.42
**SCA**																									
Instance	Opt	ELIT			ELIT_LOG			ELIT_PIECE			ELIT_SINE			ELIT_SINGER			ELIT_SINU			ELIT_TENT			ELIT_CIRCLE		
		best	median	std	best	median	std	best	median	std	best	median	std	best	median	std	best	median	std	best	median	std	best	median	std
1_1000	54,503	48,700	46,425	1051.95	49,297	46,339	1018.71	48,445	46,608	975.59	48,313	46,881	780.95	48,866	46,581	1164.87	49,053	46,603	1168.56	49,839	46,193	1340.05	49,340	47,102	1011.96
1_100	9147	9147	8817	192.98	9147	8929	215.51	9147	8940	163.26	9147	8842	198.58	9147	8911	216.25	9147	8940	125.78	9147	8810	161.29	9147	8842	181.86
1_2000	110,625	93,627	90,425	1843.25	94,817	90,424	1760.31	94,780	90,984	1675.54	95,798	90,972	1713.31	95,383	90,378	1850.16	96,188	90,146	1767.23	95,551	91,294	1802.45	94,775	90,404	1844.93
1_200	11,238	11,238	10,914	205.72	11,227	10,929	266.48	11,238	10,896	263.67	11,238	10,878	232.3	11,238	10,943	193.27	11,183	10,928	201.15	11,227	10,869	223.7	11,238	10,888	274.64
1_500	28,857	27,088	26,208	560.18	27,540	25,976	662.7	27,207	25,946	646.74	27,046	26,105	564.48	26,614	25,790	503.98	27,665	26,071	672.77	27,248	25,929	568.81	27,270	26,133	547.92
2_1000	9052	8684	8595	65.08	8752	8604	72.01	8756	8590	69.69	8746	8603	70.9	8798	8581	98.46	8706	8589	66.44	8720	8571	62.02	8808	8589	76.38
2_100	1514	1512	1498	12.82	1512	1498	8.5	1512	1496	15.36	1512	1498	10.36	1512	1496	14.1	1512	1501	10.35	1512	1496	8.93	1512	1501	12.38
2_2000	18,051	17,042	16,743	131.05	17,005	16,708	116.95	16,950	16,719	128.6	17,063	16,734	154.79	16,993	16,637	147.12	16,840	16,667	98.77	17,056	16,715	147.6	16,972	16,744	138.18
2_200	1634	1627	1617	9.25	1634	1618	9.4	1634	1622	9.76	1634	1618	11.2	1634	1619	9.31	1633	1619	9.79	1627	1617	11.29	1627	1619	11.66
2_500	4566	4509	4400	45.69	4504	4420	56.05	4515	4419	50.54	4514	4407	54.88	4479	4418	45.78	4533	4403	41.39	4491	4405	47.3	4483	4389	45.59
3_1000	14,390	13,378	13,078	135.99	13,478	13,081	160.57	13,287	12,990	145.74	13,286	12,985	155.99	13,285	13,078	112.55	13,287	13,076	144.59	13,380	13,082	150.05	13,390	12,983	175.74
3_100	2397	2390	2293	28.02	2396	2295	43.54	2396	2296	45.06	2397	2293	41.85	2397	2295	46.76	2396	2295	44.09	2397	2295	48.75	2390	2295	42.18
3_2000	28,919	25,819	25,309	243.28	25,914	25,215	243.76	25,719	25,314	204.15	25,705	25,216	225.9	25,616	25,314	151.09	25,915	25,218	263.22	26,111	25,409	269.64	25,816	25,313	212.5
3_200	2697	2697	2681	44.18	2697	2660	48.28	2695	2685	42.94	2697	2596	49.29	2697	2688	46.58	2695	2663	47.5	2697	2673	46.28	2697	2666	47.1
3_500	7117	6916	6612	100.46	6816	6705	86.84	6813	6617	77.02	6815	6698	80.56	6910	6696	82.34	6796	6690	61.8	7015	6612	102.51	6903	6616	94.55
**WOA**																									
Instance	Opt	ELIT			ELIT_LOG			ELIT_PIECE			ELIT_SINE			ELIT_SINGER			ELIT_SINU			ELIT_TENT			ELIT_CIRCLE		
		best	median	std	best	median	std	best	median	std	best	median	std	best	median	std	best	median	std	best	median	std	best	median	std
1_1000	54,503	48,421	46,439	1038.38	48,497	46,554	887.11	49,052	46,148	1112.38	48,976	46,025	1256.73	49,767	46,603	1326.69	47,838	46,535	882.01	49,784	46,943	1016.36	49,289	46,578	1214.84
1_100	9147	9147	8842	153.55	9147	8929	153.21	9147	8940	148.28	9147	8940	167.89	9147	8911	220.96	9147	8911	186.52	9147	8842	162.04	9147	8817	166.37
1_2000	110,625	93,971	91,021	1435.26	94,071	90,316	1322.2	94,861	90,476	1426.88	93,616	90,301	1568.62	95,689	90,161	1676.85	93,962	90,578	1592.57	94,309	90,237	1514.36	93,525	90,608	1629.1
1_200	11,238	11,238	10,954	281.97	11,238	10,888	244.73	11,238	10,874	247.95	11,227	10,805	245.09	11,238	10,888	242.19	11,223	10,896	156.75	11,238	10,727	245.93	11,227	10,736	262.77
1_500	28,857	27,670	26,312	797.28	28,187	26,042	710.75	27,241	25,944	607.92	27,318	25,894	536.28	27,655	25,922	576.58	27,717	26,169	818.26	27,442	26,031	682.12	27,296	25,983	617.32
2_1000	9052	8720	8588	76.74	8677	8575	62.88	8748	8585	64.83	8734	8603	83.96	8737	8581	70.93	8732	8551	61.82	8802	8582	86.5	8780	8583	76.95
2_100	1514	1512	1498	8.14	1512	1496	10.03	1512	1496	15.1	1512	1501	12.19	1512	1496	8.05	1512	1498	11.45	1512	1496	11.01	1512	1496	9.66
2_2000	18,051	16,969	16,714	129.28	16,990	16,771	136.61	17,039	16,725	151.51	17,009	16,742	129.97	16,957	16,699	137.96	17,135	16,663	154.31	16,990	16,683	119.25	17,124	16,769	140.78
2_200	1634	1634	1618	11.04	1633	1617	11.47	1634	1619	12.72	1634	1618	10.21	1633	1617	8.01	1634	1617	13.83	1634	1617	9.71	1634	1619	8.17
2_500	4566	4509	4397	43.26	4526	4418	44.49	4472	4408	43.49	4507	4395	52.38	4535	4404	56.51	4486	4398	54.04	4508	4404	42.6	4473	4402	51.8
3_1000	14,390	13,385	12,979	193.13	13,482	13,079	123.46	13,283	13,080	132.05	13,284	12,989	131.51	13,289	12,986	142.62	13,484	13,076	197.84	13,482	13,075	218.26	13,380	13,079	133.89
3_100	2397	2396	2294	41.17	2390	2295	44.97	2396	2294	43.07	2396	2293	44.33	2397	2295	41.95	2397	2295	35.95	2390	2296	45.9	2397	2292	37.92
3_2000	28,919	26,418	25,309	324.95	25,809	25,217	231.92	25,818	25,319	203.52	26,011	25,309	247.81	26,010	25,309	259.49	25,611	25,217	205.09	25,910	25,306	241.29	26,013	25,219	241.65
3_200	2697	2697	2686	46.56	2694	2596	45.92	2697	2597	48.1	2697	2673	49.7	2697	2684	43.83	2697	2684	49.59	2697	2684	46.6	2695	2597	46.34
3_500	7117	6904	6694	94.83	6908	6699	99.18	6816	6615	86.66	7016	6683	101.17	6815	6698	72.7	6808	6680	83.98	6908	6612	78.66	6914	6701	89.33

**Table A3:** Statistical performance summary of the proposed chaotic binarization framework for the 0–1 Knapsack Problem (KP) instances. Columns report the known global optimum (Opt), best fitness achieved, median, and standard deviation (std) calculated over 31 independent runs for the GWO, SCA, and WOA metaheuristics under the Standard (STD) rule across different chaotic map configurations.

**GWO**																									
Instance	Opt	STD			STD_LOG			STD_PIECE			STD_SINE			STD_SINGER			STD_SINU			STD_TENT			STD_CIRCLE		
		best	median	std	best	median	std	best	median	std	best	median	std	best	median	std	best	median	std	best	median	std	best	median	std
1_1000	54,503	53,838	53,392	245.44	52,617	52,158	286.52	53,702	53,206	239.25	53,673	52,866	380.91	49,162	48,016	490.34	48,934	46,759	1017.94	53,760	53,346	205.52	54,264	54,038	62.63
1_100	9147	9147	9147	0.0	9147	9147	0.0	9147	9147	0.0	9147	9147	0.0	9147	9147	0.0	9147	9147	0.0	9147	9147	0.0	9147	9147	0.0
1_2000	110,625	106,338	104,519	667.82	103,254	101,805	671.8	105,308	104,308	447.58	104,502	103,306	509.83	94,623	91,362	1022.83	95,130	90,465	1458.55	106,008	105,134	493.16	108,462	108,080	163.02
1_200	11,238	11,238	11,238	0.0	11,238	11,238	0.0	11,238	11,238	0.0	11,238	11,238	0.0	11,238	11,238	0.0	11,238	11,238	0.0	11,238	11,238	0.0	11,238	11,238	0.0
1_500	28,857	28,857	28,834	17.12	28,834	28,682	116.25	28,857	28,834	35.4	28,857	28,759	41.2	28,328	27,596	231.02	27,513	26,200	392.31	28,857	28,834	16.14	28,857	28,857	10.61
2_1000	9052	9030	9011	12.01	8997	8964	17.02	9033	9009	11.01	9029	8992	12.37	8888	8766	42.8	8738	8589	79.24	9032	9003	15.02	9028	9010	5.95
2_100	1514	1512	1512	0.0	1512	1512	0.0	1512	1512	0.0	1512	1512	0.0	1512	1512	0.0	1512	1512	0.0	1512	1512	0.0	1512	1512	0.0
2_2000	18,051	17,818	17,727	39.25	17,640	17,547	40.78	17,778	17,707	34.1	17,706	17,654	26.92	17,081	16,852	98.49	16,932	16,698	126.43	17,787	17,715	30.76	17,970	17,918	21.05
2_200	1634	1634	1634	0.0	1634	1634	0.0	1634	1634	0.0	1634	1634	0.0	1634	1634	0.25	1634	1633	0.49	1634	1634	0.0	1634	1634	2.36
2_500	4566	4566	4559	4.4	4566	4554	5.72	4566	4566	0.0	4566	4566	0.0	4544	4517	9.76	4534	4455	26.84	4566	4559	4.08	4557	4551	1.12
3_1000	14,390	14,189	14,089	44.28	13,988	13,889	37.28	14,189	14,089	36.57	14,187	14,078	79.42	13,387	13,190	90.64	13,381	13,075	166.62	14,188	14,090	45.41	14,290	14,290	3.2
3_100	2397	2397	2397	0.0	2397	2397	0.0	2397	2397	0.0	2397	2397	0.0	2397	2397	0.0	2397	2397	0.0	2397	2397	0.0	2397	2396	0.3
3_2000	28,919	27,910	27,708	86.79	27,315	27,112	89.7	27,811	27,619	69.31	27,616	27,409	75.72	25,815	25,315	223.32	25,818	25,310	257.77	28,112	27,803	125.45	28,419	28,317	47.07
3_200	2697	2697	2697	0.0	2697	2697	0.0	2697	2697	0.0	2697	2697	0.0	2697	2697	0.0	2697	2697	0.0	2697	2697	0.0	2697	2697	0.0
3_500	7117	7117	7116	17.82	7017	7017	0.25	7117	7115	29.38	7117	7017	48.19	6914	6817	28.75	6816	6676	82.41	7117	7114	24.5	7117	7117	0.0
**SCA**																									
Instance	Opt	STD			STD_LOG			STD_PIECE			STD_SINE			STD_SINGER			STD_SINU			STD_TENT			STD_CIRCLE		
		best	median	std	best	median	std	best	median	std	best	median	std	best	median	std	best	median	std	best	median	std	best	median	std
1_1000	54,503	53,681	52,959	290.58	52,931	51,840	420.52	53,662	52,649	317.46	53,318	52,702	311.23	49,265	47,856	559.55	48,741	46,386	975.47	53,416	52,859	238.22	54,234	54,038	100.87
1_100	9147	9147	9147	0.0	9147	9147	0.0	9147	9147	0.0	9147	9147	0.0	9147	9147	0.0	9147	9147	0.0	9147	9147	0.0	9147	9147	0.0
1_2000	110,625	105,363	103,730	698.52	102,554	101,501	695.39	105,728	103,287	779.41	103,910	102,574	468.36	95,257	90,410	1560.6	95,017	90,992	1613.87	106,046	104,068	820.37	108,845	108,467	262.64
1_200	11,238	11,238	11,238	0.0	11,238	11,238	0.0	11,238	11,238	0.0	11,238	11,238	0.0	11,238	11,238	1.98	11,238	11,238	2.75	11,238	11,238	0.0	11,238	11,238	0.0
1_500	28,857	28,857	28,801	64.58	28,764	28,596	47.18	28,834	28,794	31.8	28,857	28,688	55.93	28,182	27,460	264.39	27,261	26,147	450.18	28,857	28,819	59.34	28,857	28,857	5.74
2_1000	9052	9045	8989	17.02	9001	8948	21.59	9030	8986	18.08	9013	8992	18.7	8827	8746	41.54	8729	8572	70.39	9028	8987	12.41	9030	9013	6.34
2_100	1514	1512	1512	0.0	1512	1512	0.0	1512	1512	0.0	1512	1512	0.0	1512	1512	0.0	1513	1512	0.18	1512	1512	0.0	1512	1512	0.0
2_2000	18,051	17,794	17,662	52.53	17,643	17,497	58.32	17,757	17,652	43.42	17,736	17,632	55.84	17,005	16,804	76.03	17,111	16,738	167.15	17,773	17,643	36.33	17,974	17,939	7.79
2_200	1634	1634	1634	0.0	1634	1634	0.0	1634	1634	0.0	1634	1634	0.0	1634	1634	0.0	1634	1633	3.13	1634	1634	0.0	1634	1634	0.0
2_500	4566	4566	4557	4.43	4566	4554	3.58	4566	4559	3.73	4566	4566	0.0	4551	4508	14.83	4501	4437	27.27	4566	4566	5.1	4566	4552	4.95
3_1000	14,390	14,090	14,067	46.55	13,990	13,881	57.59	14,090	13,990	48.26	14,087	13,988	37.57	13,467	13,176	93.67	13,286	13,072	130.91	14,187	14,083	50.41	14,290	14,290	17.96
3_100	2397	2397	2397	0.0	2397	2397	0.0	2397	2397	0.0	2397	2397	0.0	2397	2397	0.0	2397	2397	0.0	2397	2397	0.0	2397	2396	0.3
3_2000	28,919	27,716	27,512	99.55	27,214	27,011	103.95	27,619	27,511	84.92	27,418	27,312	69.7	25,618	25,219	167.79	26,012	25,411	254.9	27,814	27,516	120.89	28,518	28,405	55.36
3_200	2697	2697	2697	0.0	2697	2697	0.0	2697	2697	0.0	2697	2697	0.0	2697	2697	0.0	2697	2697	0.75	2697	2697	0.0	2697	2697	0.0
3_500	7117	7116	7108	48.89	7017	7017	1.11	7116	7017	47.14	7116	7017	27.39	6915	6814	30.07	6815	6617	78.4	7117	7108	42.58	7117	7117	0.0
**WOA**																									
Instance	Opt	STD			STD_LOG			STD_PIECE			STD_SINE			STD_SINGER			STD_SINU			STD_TENT			STD_CIRCLE		
		best	median	std	best	median	std	best	median	std	best	median	std	best	median	std	best	median	std	best	median	std	best	median	std
1_1000	54,503	54,485	54,341	70.63	54,205	53,941	146.86	54,503	54,356	74.72	54,503	54,112	162.1	53,458	52,812	292.38	52,687	52,163	397.65	54,503	54,380	75.97	54,481	54,481	22.14
1_100	9147	9147	9147	0.0	9147	9147	0.0	9147	9147	0.0	9147	9147	0.0	9147	9147	0.0	9147	9147	0.0	9147	9147	0.0	9147	9147	0.0
1_2000	110,625	109,623	108,837	297.94	108,467	107,012	595.05	109,791	108,961	326.56	108,598	107,785	338.8	104,903	103,719	610.55	103,389	100,389	861.56	109,959	109,079	309.98	110,555	110,218	162.54
1_200	11,238	11,238	11,238	0.0	11,238	11,238	0.0	11,238	11,238	0.0	11,238	11,238	0.0	11,238	11,238	0.0	11,238	11,238	0.0	11,238	11,238	0.0	11,238	11,238	0.0
1_500	28,857	28,857	28,857	4.13	28,857	28,857	11.68	28,857	28,857	4.13	28,857	28,857	10.93	28,857	28,794	80.86	28,857	28,675	105.58	28,857	28,857	8.6	28,857	28,834	4.13
2_1000	9052	9051	9051	1.71	9048	9031	9.6	9051	9051	1.92	9051	9046	6.32	9027	8986	19.36	9006	8970	20.59	9051	9051	1.73	9051	9051	1.49
2_100	1514	1512	1512	0.0	1512	1512	0.0	1512	1512	0.0	1512	1512	0.0	1512	1512	0.0	1513	1512	0.3	1512	1512	0.0	1512	1512	0.0
2_2000	18,051	18,027	17,970	23.71	17,960	17,871	42.97	18,022	17,972	18.96	17,981	17,902	28.69	17,774	17,651	54.16	17,626	17,494	59.67	18,009	17,968	23.35	18,040	18,023	5.15
2_200	1634	1634	1634	0.0	1634	1634	0.0	1634	1634	0.0	1634	1634	0.0	1634	1634	0.0	1634	1634	0.0	1634	1634	0.0	1634	1634	0.0
2_500	4566	4566	4566	2.01	4566	4566	2.16	4566	4566	0.0	4566	4566	1.75	4566	4556	4.94	4566	4559	5.31	4566	4566	0.0	4552	4551	0.46
3_1000	14,390	14,390	14,290	48.04	14,290	14,262	46.49	14,389	14,290	46.59	14,290	14,288	24.69	14,186	14,076	55.39	13,989	13,886	61.87	14,390	14,290	41.48	14,390	14,390	0.56
3_100	2397	2397	2397	0.0	2397	2397	0.0	2397	2397	0.0	2397	2397	0.0	2397	2397	0.0	2397	2397	0.0	2397	2397	0.0	2396	2396	0.0
3_2000	28,919	28,809	28,617	71.1	28,319	28,214	70.61	28,713	28,606	50.33	28,519	28,387	69.19	27,808	27,517	124.76	27,416	27,004	193.92	28,719	28,611	69.17	28,916	28,818	33.38
3_200	2697	2697	2697	0.0	2697	2697	0.0	2697	2697	0.0	2697	2697	0.0	2697	2697	0.0	2697	2697	0.0	2697	2697	0.0	2697	2697	0.0
3_500	7117	7117	7117	0.0	7117	7117	0.3	7117	7117	0.0	7117	7117	0.0	7117	7017	47.17	7117	7017	24.04	7117	7117	0.0	7117	7117	0.0

**Table A4:** Statistical performance summary of the proposed chaotic binarization framework for the Set Covering Problem (SCP) instances. Columns report the known global optimum (Opt), best fitness achieved, median, and standard deviation (std) calculated over 31 independent runs for the GWO, SCA, and WOA metaheuristics under the Complement (COM) rule across different chaotic map configurations.

**GWO**																									
Instance	Opt	COM			COM_LOG			COM_PIECE			COM_SINE			COM_SINGER			COM_SINU			COM_TENT			COM_CIRCLE		
		best	median	std	best	median	std	best	median	std	best	median	std	best	median	std	best	median	std	best	median	std	best	median	std
41	429	449	458	3.02	449	457	3.48	446	457	3.48	450	457	3.27	452	460	2.66	449	458	3.27	448	457	3.28	454	459	1.87
51	253	274	280	3.25	276	281	3.15	278	280	2.29	274	279	1.93	277	282	2.99	280	286	2.36	268	279	3.02	276	279	1.69
61	138	143	150	2.86	145	152	2.63	145	151	2.9	143	151	3.25	145	152	2.82	145	153	2.82	144	152	3.09	145	151	2.64
a1	253	271	276	3.05	272	277	2.95	271	278	2.8	269	277	3.91	274	279	3.03	275	279	2.6	271	277	3.27	271	276	2.57
b1	69	71	72	0.67	71	72	0.82	71	71	0.8	71	71	0.72	71	73	0.96	72	73	0.78	71	72	0.67	71	72	0.66
c1	227	246	251	1.75	246	251	1.8	248	251	1.11	247	251	1.52	248	252	1.54	251	253	1.3	248	251	1.59	248	251	1.71
d1	60	64	65	0.69	64	65	0.64	63	65	0.75	63	65	0.65	64	65	0.73	64	65	1.17	63	65	0.72	63	65	0.62
nre1	29	29	29	0.0	29	29	0.18	29	29	0.18	29	29	0.18	29	29	0.4	29	30	0.5	29	29	0.18	29	29	0.0
nrf1	14	14	14	0.0	14	14	0.0	14	14	0.0	14	14	0.0	14	14	0.0	14	14	0.0	14	14	0.0	14	14	0.0
**SCA**																									
Instance	Opt	COM			COM_LOG			COM_PIECE			COM_SINE			COM_SINGER			COM_SINU			COM_TENT			COM_CIRCLE		
		best	median	std	best	median	std	best	median	std	best	median	std	best	median	std	best	median	std	best	median	std	best	median	std
41	429	440	447	3.27	440	447	2.77	440	447	2.79	440	448	3.16	442	446	2.31	437	447	3.01	440	447	2.78	441	448	3.14
51	253	276	280	2.32	275	281	2.38	270	280	2.74	277	280	1.77	274	280	2.16	273	279	2.87	273	280	2.31	275	281	2.37
61	138	146	150	1.67	145	150	1.92	146	151	1.73	145	150	2.4	143	149	2.47	143	148	2.16	145	149	1.95	145	148	2.11
a1	253	269	273	2.55	266	273	2.35	267	274	2.46	270	275	2.13	267	274	2.35	267	274	2.72	269	276	2.92	269	274	2.05
b1	69	72	73	1.11	72	72	0.89	72	72	1.01	72	73	0.94	72	73	0.99	72	73	0.87	72	73	1.16	72	72	0.97
c1	227	244	247	1.57	241	247	1.96	243	248	1.93	243	247	2.06	244	248	2.05	243	247	2.21	242	247	2.33	244	248	1.77
d1	60	64	66	0.96	64	65	0.65	64	66	0.95	64	65	1.06	64	66	0.88	64	66	0.85	64	65	0.73	64	65	1.06
nre1	29	29	29	0.34	29	29	0.4	29	29	0.44	29	29	0.43	29	29	0.5	29	29	0.46	29	29	0.44	29	29	0.4
nrf1	14	14	14	0.18	14	14	0.18	14	14	0.25	14	14	0.0	14	14	0.25	14	14	0.18	14	14	0.18	14	14	0.0
**WOA**																									
Instance	Opt	COM			COM_LOG			COM_PIECE			COM_SINE			COM_SINGER			COM_SINU			COM_TENT			COM_CIRCLE		
		best	median	std	best	median	std	best	median	std	best	median	std	best	median	std	best	median	std	best	median	std	best	median	std
41	429	436	445	5.0	438	447	4.63	440	446	3.53	439	445	3.74	441	446	2.92	443	449	3.51	434	445	4.04	441	447	2.89
51	253	273	279	3.46	271	277	3.17	273	279	2.05	274	279	2.32	272	280	2.8	271	279	3.27	268	278	3.73	270	278	3.02
61	138	144	149	2.8	144	149	2.07	142	149	3.11	144	148	3.0	144	149	2.51	144	150	2.63	144	149	2.27	142	145	2.46
a1	253	265	272	3.65	264	270	3.45	266	273	3.33	261	271	4.09	267	272	2.72	267	273	3.7	267	272	2.93	263	271	3.66
b1	69	69	72	1.07	71	72	1.06	71	72	1.0	71	72	1.0	71	72	0.52	71	72	1.0	71	72	0.96	72	72	0.93
c1	227	242	247	2.36	239	247	3.23	242	245	2.51	241	246	2.53	241	246	2.65	240	247	3.25	241	245	2.81	239	246	2.5
d1	60	63	64	0.81	63	65	0.68	63	64	0.72	63	64	0.71	63	65	0.75	63	65	0.78	62	64	0.77	63	64	0.79
nre1	29	29	29	0.34	29	29	0.18	29	29	0.18	29	29	0.0	29	29	0.3	29	29	0.5	29	29	0.0	29	29	0.3
nrf1	14	14	14	0.18	14	14	0.0	14	14	0.25	14	14	0.18	14	14	0.18	14	14	0.18	14	14	0.25	14	14	0.0

**Table A5:** Statistical performance summary of the proposed chaotic binarization framework for the Set Covering Problem (SCP) instances. Columns report the known global optimum (Opt), best fitness achieved, median, and standard deviation (std) calculated over 31 independent runs for the GWO, SCA, and WOA metaheuristics under the Elitist (ELIT) rule across different chaotic map configurations.

**GWO**																									
Instance	Opt	ELIT			ELIT_LOG			ELIT_PIECE			ELIT_SINE			ELIT_SINGER			ELIT_SINU			ELIT_TENT			ELIT_CIRCLE		
		best	median	std	best	median	std	best	median	std	best	median	std	best	median	std	best	median	std	best	median	std	best	median	std
41	429	433	433	1.5	433	433	0.81	431	433	1.61	431	433	1.83	434	436	0.68	435	436	0.6	433	433	1.78	433	434	2.15
51	253	267	267	0.62	256	268	2.79	265	268	0.91	257	267	1.91	267	268	0.62	268	269	0.48	267	267	0.72	267	269	2.06
61	138	141	141	1.92	141	143	1.97	141	144	1.93	141	145	1.66	141	142	1.74	141	142	0.5	141	143	1.66	141	146	2.45
a1	253	257	257	0.25	257	258	0.59	257	257	0.34	257	257	0.68	257	259	1.47	259	261	1.1	257	257	0.73	257	257	0.84
b1	69	69	71	0.96	69	71	1.03	69	71	1.17	69	71	0.96	69	71	0.93	69	70	0.67	69	71	1.02	70	71	1.23
c1	227	231	234	0.93	232	235	0.98	232	233	0.88	233	235	0.89	234	237	1.02	235	237	1.02	232	234	0.75	232	234	0.99
d1	60	60	62	1.03	60	62	1.0	60	62	0.88	60	61	1.03	60	62	1.02	62	63	0.56	61	62	0.82	61	62	1.67
nre1	29	29	29	0.0	29	29	0.18	29	29	0.18	29	29	0.0	29	29	0.0	29	29	0.0	29	29	0.0	29	29	0.3
nrf1	14	14	14	0.34	14	14	0.34	14	14	0.44	14	14	0.49	14	14	0.3	14	14	0.0	14	14	0.44	14	15	0.5
**SCA**																									
Instance	Opt	ELIT			ELIT_LOG			ELIT_PIECE			ELIT_SINE			ELIT_SINGER			ELIT_SINU			ELIT_TENT			ELIT_CIRCLE		
		best	median	std	best	median	std	best	median	std	best	median	std	best	median	std	best	median	std	best	median	std	best	median	std
41	429	433	433	1.66	433	433	1.39	433	433	1.86	433	433	1.9	434	436	1.04	435	436	1.28	433	433	1.74	433	434	2.14
51	253	267	268	0.89	258	267	1.94	257	268	2.09	267	267	1.18	258	268	2.49	267	269	0.62	256	268	2.88	267	268	1.59
61	138	141	145	1.53	141	143	1.89	141	145	1.71	141	145	1.51	141	144	1.75	141	142	2.08	141	145	1.87	141	145	2.23
a1	253	257	257	0.44	257	258	0.86	257	257	0.5	257	258	0.6	258	260	1.57	258	261	1.59	257	257	0.51	257	257	0.92
b1	69	69	70	1.38	69	70	0.98	69	71	0.99	69	70	1.16	69	71	0.85	69	70	0.91	69	71	1.09	69	71	1.11
c1	227	232	234	1.29	232	235	1.14	231	234	1.24	232	235	1.12	235	238	0.9	236	238	0.96	230	234	1.37	232	234	1.23
d1	60	60	62	0.75	60	62	0.73	60	62	1.09	60	62	0.93	60	62	0.73	61	63	0.72	60	62	1.25	61	62	1.07
nre1	29	29	29	0.0	29	29	0.25	29	29	0.18	29	29	0.25	29	29	0.18	29	29	0.0	29	29	0.0	29	29	0.3
nrf1	14	14	14	0.37	14	14	0.34	14	14	0.49	14	14	0.43	14	14	0.4	14	14	0.18	14	14	0.37	14	14	0.5
**WOA**																									
Instance	Opt	ELIT			ELIT_LOG			ELIT_PIECE			ELIT_SINE			ELIT_SINGER			ELIT_SINU			ELIT_TENT			ELIT_CIRCLE		
		best	median	std	best	median	std	best	median	std	best	median	std	best	median	std	best	median	std	best	median	std	best	median	std
41	429	433	433	1.95	433	433	1.26	433	433	2.01	433	433	1.85	433	433	0.18	433	433	1.2	433	433	2.03	433	439	3.83
51	253	256	267	2.32	266	267	1.04	267	268	1.55	267	268	0.82	267	268	0.97	267	268	0.75	256	268	2.56	268	274	3.66
61	138	141	145	1.34	141	145	1.66	141	144	1.93	141	145	1.73	141	142	1.76	139	142	1.73	141	144	1.97	141	147	3.51
a1	253	257	257	0.25	257	257	1.06	256	257	1.23	256	257	1.06	257	257	1.09	257	257	0.96	257	257	0.5	257	260	1.54
b1	69	69	71	1.1	69	71	0.83	69	71	0.89	69	71	1.06	69	71	1.15	69	69	1.01	69	71	1.26	70	72	1.52
c1	227	231	234	1.25	231	234	1.42	230	234	1.62	231	234	1.48	233	234	0.96	233	234	0.89	231	234	1.4	233	234	1.61
d1	60	60	62	1.63	60	62	0.92	60	62	1.72	60	62	1.7	60	62	0.94	60	62	0.8	61	62	1.15	61	63	2.06
nre1	29	29	29	0.43	29	29	0.18	29	29	0.18	29	29	0.25	29	29	0.25	29	29	0.0	29	29	0.3	29	29	0.72
nrf1	14	14	14	0.46	14	14	0.4	14	14	0.5	14	14	0.5	14	14	0.46	14	14	0.25	14	14	0.5	14	15	0.43

**Table A6:** Statistical performance summary of the proposed chaotic binarization framework for the Set Covering Problem (SCP) instances. Columns report the known global optimum (Opt), best fitness achieved, median, and standard deviation (std) calculated over 31 independent runs for the GWO, SCA, and WOA metaheuristics under the Standard (STD) rule across different chaotic map configurations.

**GWO**																									
Instance	Opt	STD			STD_LOG			STD_PIECE			STD_SINE			STD_SINGER			STD_SINU			STD_TENT			STD_CIRCLE		
		best	median	std	best	median	std	best	median	std	best	median	std	best	median	std	best	median	std	best	median	std	best	median	std
41	429	433	435	1.1	435	437	1.0	434	434	1.01	435	438	0.77	435	437	1.01	434	436	0.86	433	435	0.79	435	440	2.49
51	253	267	269	0.92	269	271	0.98	267	268	1.01	269	272	1.45	268	270	0.97	268	269	0.43	267	268	0.82	268	271	3.75
61	138	141	142	1.78	141	143	1.76	141	142	1.52	143	144	2.38	142	142	0.51	141	142	0.26	141	141	1.89	142	151	3.48
a1	253	258	260	1.5	261	264	1.46	259	261	1.18	262	265	1.44	259	261	1.0	260	261	1.03	258	261	1.26	259	262	1.39
b1	69	69	71	0.84	70	71	0.71	69	71	1.03	70	71	0.66	69	71	0.56	69	70	0.66	69	71	0.92	70	72	1.33
c1	227	235	237	1.07	238	240	1.12	235	237	1.13	237	240	1.37	237	239	0.88	235	238	1.1	236	237	0.68	238	240	1.2
d1	60	61	62	0.53	62	63	0.5	62	63	0.51	62	63	0.75	62	63	0.41	61	62	0.6	61	62	0.56	61	62	0.92
nre1	29	29	29	0.0	29	29	0.0	29	29	0.0	29	29	0.0	29	29	0.0	29	29	0.0	29	29	0.0	29	29	0.18
nrf1	14	14	14	0.4	14	15	0.4	14	14	0.18	14	15	0.51	14	14	0.0	14	14	0.0	14	14	0.25	14	15	0.51
**SCA**																									
Instance	Opt	STD			STD_LOG			STD_PIECE			STD_SINE			STD_SINGER			STD_SINU			STD_TENT			STD_CIRCLE		
		best	median	std	best	median	std	best	median	std	best	median	std	best	median	std	best	median	std	best	median	std	best	median	std
41	429	434	435	1.5	435	437	1.06	434	436	1.51	434	438	1.59	437	438	0.37	435	438	1.23	434	436	1.53	436	438	2.42
51	253	267	269	0.87	268	270	0.98	267	270	1.44	268	270	1.06	268	271	1.45	265	271	1.51	267	269	1.19	268	270	2.38
61	138	141	146	2.42	142	145	1.87	141	146	2.26	141	143	2.27	142	143	0.6	142	143	1.0	141	145	2.53	142	147	1.94
a1	253	259	261	1.67	261	262	1.32	258	263	1.75	258	263	1.71	260	262	1.06	262	263	1.26	258	262	1.59	259	263	1.85
b1	69	69	71	0.79	69	71	0.84	69	71	0.96	69	71	0.91	70	71	0.7	70	71	0.5	69	70	0.85	70	72	0.81
c1	227	236	239	1.43	236	239	1.28	236	238	1.09	236	239	1.29	239	240	0.96	236	240	1.49	236	238	1.06	236	238	1.59
d1	60	61	63	0.62	62	63	0.51	61	63	0.76	61	63	0.77	61	63	0.62	62	63	0.43	62	63	0.54	61	63	1.06
nre1	29	29	29	0.0	29	29	0.0	29	29	0.0	29	29	0.25	29	29	0.0	29	29	0.0	29	29	0.0	29	29	0.18
nrf1	14	14	14	0.44	14	14	0.46	14	14	0.34	14	14	0.51	14	14	0.0	14	14	0.0	14	14	0.46	14	15	0.51
**WOA**																									
Instance	Opt	STD			STD_LOG			STD_PIECE			STD_SINE			STD_SINGER			STD_SINU			STD_TENT			STD_CIRCLE		
		best	median	std	best	median	std	best	median	std	best	median	std	best	median	std	best	median	std	best	median	std	best	median	std
41	429	433	434	2.09	433	436	1.92	431	433	1.97	433	435	2.96	435	437	0.77	433	436	1.06	433	435	1.47	439	444	3.53
51	253	267	268	1.42	267	269	1.23	267	269	1.83	267	269	1.33	267	269	1.48	268	269	0.4	267	268	1.1	269	274	5.25
61	138	141	145	1.51	141	145	1.98	141	145	2.0	142	145	1.56	141	142	1.42	141	142	1.7	141	145	1.69	142	147	4.25
a1	253	257	258	1.39	258	261	1.6	257	259	1.53	259	261	1.65	258	260	1.88	258	261	1.81	257	259	1.21	258	261	1.63
b1	69	69	71	1.09	69	71	0.86	69	71	0.68	70	71	0.82	69	71	0.86	69	71	0.81	69	71	0.93	70	72	0.99
c1	227	233	236	1.47	237	238	1.09	234	236	1.6	235	237	1.08	237	239	1.17	235	237	1.2	234	236	1.09	235	238	2.3
d1	60	61	62	0.6	61	62	0.61	60	62	0.85	61	62	1.28	60	63	0.81	61	62	0.74	61	62	1.31	61	64	2.15
nre1	29	29	29	0.34	29	29	0.0	29	29	0.25	29	29	0.3	29	29	0.25	29	29	0.0	29	29	0.34	29	29	0.81
nrf1	14	14	14	0.51	14	14	0.51	14	14	0.5	14	15	0.56	14	14	0.43	14	14	0.18	14	14	0.51	15	15	0.0

**Table A7:** Statistical performance summary of the proposed chaotic binarization framework for the Unicost Set Covering Problem (USCP) instances. Columns report the known global optimum (Opt), best fitness achieved, median, and standard deviation (std) calculated over 31 independent runs for the GWO, SCA, and WOA metaheuristics under the Complement (COM) rule across different chaotic map configurations.

**GWO**																									
Instance	Opt	COM			COM_LOG			COM_PIECE			COM_SINE			COM_SINGER			COM_SINU			COM_TENT			COM_CIRCLE		
		best	median	std	best	median	std	best	median	std	best	median	std	best	median	std	best	median	std	best	median	std	best	median	std
u41	38	41	42	0.73	42	44	0.72	40	42	0.6	42	44	0.91	40	41	0.46	39	40	0.43	41	42	0.71	41	42	0.7
u51	34	37	38	0.56	38	39	0.54	37	38	0.43	38	40	0.81	36	37	0.51	35	36	0.48	36	38	0.54	37	38	0.43
u61	21	22	22	0.48	22	23	0.5	22	22	0.5	22	23	0.58	21	22	0.5	21	21	0.0	22	22	0.46	21	22	0.53
uclr10	25	28	29	0.51	28	29	0.62	28	29	0.66	28	30	0.57	27	28	0.7	26	28	0.73	28	29	0.45	28	29	0.52
uclr11	23	27	28	0.61	28	29	0.41	27	28	0.57	28	29	0.58	25	27	1.12	25	25	0.62	27	28	0.6	26	28	0.81
uclr12	23	28	30	0.66	30	30	0.48	28	30	0.53	29	31	0.57	26	28	0.84	26	28	0.75	28	30	0.71	29	30	0.46
uclr13	23	29	31	0.66	31	31	0.44	30	31	0.4	30	32	0.55	28	30	0.66	27	30	0.84	28	31	0.6	30	31	0.4
ucyc06	60	63	63	0.51	63	64	0.57	63	63	0.51	63	64	0.7	60	62	1.18	60	60	0.0	62	63	0.65	62	63	0.68
ucyc07	144	159	160	0.72	157	162	1.19	157	160	1.17	160	162	1.24	156	158	0.96	144	146	1.12	157	160	1.08	157	160	1.09
ud1	24	26	27	0.51	26	28	0.69	26	27	0.43	27	28	0.62	25	26	0.34	25	25	0.0	26	27	0.44	25	26	0.56
unrf1	10	10	11	0.48	10	11	0.18	10	11	0.46	10	11	0.18	10	10	0.25	10	10	0.3	10	11	0.4	10	11	0.5
**SCA**																									
Instance	Opt	COM			COM_LOG			COM_PIECE			COM_SINE			COM_SINGER			COM_SINU			COM_TENT			COM_CIRCLE		
		best	median	std	best	median	std	best	median	std	best	median	std	best	median	std	best	median	std	best	median	std	best	median	std
u41	38	39	41	0.53	39	41	0.66	40	41	0.34	39	41	0.59	39	41	0.48	39	41	0.56	40	41	0.3	39	41	0.48
u51	34	35	36	0.56	35	35	0.5	35	36	0.6	35	36	0.66	35	35	0.5	35	36	0.5	35	36	0.65	35	36	0.6
u61	21	21	21	0.72	21	21	0.57	21	22	0.56	21	22	0.56	21	21	0.55	21	21	0.51	21	22	0.6	21	22	0.74
uclr10	25	25	27	0.98	26	27	0.66	25	28	0.98	25	28	0.92	27	28	0.58	26	28	0.85	25	27	0.88	25	27	0.9
uclr11	23	23	25	0.92	23	25	1.15	23	24	0.91	23	25	1.23	23	25	0.77	23	25	0.71	23	25	0.82	23	25	1.17
uclr12	23	25	26	0.77	26	26	0.8	25	26	0.81	25	26	0.93	26	27	0.81	26	27	0.98	25	27	0.94	25	26	0.89
uclr13	23	26	29	1.2	27	29	1.11	27	29	1.08	25	29	1.37	26	29	1.14	27	29	1.11	27	29	1.12	26	30	0.94
ucyc06	60	60	60	0.0	60	60	0.0	60	60	0.0	60	60	0.0	60	60	0.0	60	60	0.0	60	60	0.0	60	60	0.0
ucyc07	144	144	145	0.86	144	145	0.91	144	145	0.88	144	146	1.06	144	145	0.62	144	145	0.74	144	145	0.88	144	145	0.81
ud1	24	25	25	0.55	25	26	0.5	25	25	0.6	25	25	0.57	25	26	0.51	25	25	0.48	25	25	0.49	25	25	0.55
unrf1	10	10	11	0.51	10	11	0.51	10	11	0.5	10	11	0.5	10	11	0.5	10	11	0.5	10	11	0.44	10	11	0.48
**WOA**																									
Instance	Opt	COM			COM_LOG			COM_PIECE			COM_SINE			COM_SINGER			COM_SINU			COM_TENT			COM_CIRCLE		
		best	median	std	best	median	std	best	median	std	best	median	std	best	median	std	best	median	std	best	median	std	best	median	std
u41	38	39	41	0.72	39	41	0.76	39	41	0.65	39	41	0.62	39	41	0.56	40	41	0.3	39	41	0.5	39	41	0.43
u51	34	35	36	0.53	35	36	0.5	35	35	0.62	35	36	0.54	35	36	0.56	35	36	0.84	35	35	0.51	35	36	0.56
u61	21	21	22	0.64	21	22	0.56	21	22	0.53	21	22	0.5	21	21	0.5	21	22	0.8	21	22	0.64	21	22	0.7
uclr10	25	25	26	1.18	25	27	1.24	25	27	1.19	25	26	1.21	25	28	1.2	25	26	1.31	25	27	1.26	25	27	1.27
uclr11	23	23	24	1.09	23	24	1.51	23	23	1.49	23	23	1.6	23	24	1.19	23	24	1.47	23	23	1.19	23	23	1.68
uclr12	23	23	26	1.65	24	26	1.27	24	26	1.45	23	26	1.66	25	26	0.96	23	26	1.67	23	26	1.54	23	26	1.11
uclr13	23	24	29	1.68	25	29	1.29	25	29	1.47	24	29	1.86	26	29	1.28	25	30	1.45	25	29	1.56	25	30	1.37
ucyc06	60	60	60	0.0	60	60	0.0	60	60	0.0	60	60	0.0	60	60	0.0	60	60	0.0	60	60	0.0	60	60	0.0
ucyc07	144	144	144	0.67	144	144	0.92	144	144	0.3	144	144	0.62	144	144	0.68	144	144	0.58	144	144	0.5	144	144	0.3
ud1	24	25	26	0.7	25	25	0.51	25	26	0.71	25	26	0.62	25	25	0.5	25	26	0.71	25	26	0.68	25	25	0.63
unrf1	10	10	11	0.44	10	11	0.48	10	11	0.51	10	11	0.48	10	11	0.51	10	11	0.34	10	11	0.48	10	11	0.46

**Table A8:** Statistical performance summary of the proposed chaotic binarization framework for the Unicost Set Covering Problem (USCP) instances. Columns report the known global optimum (Opt), best fitness achieved, median, and standard deviation (std) calculated over 31 independent runs for the GWO, SCA, and WOA metaheuristics under the Elitist (ELIT) rule across different chaotic map configurations.

**GWO**																									
Instance	Opt	ELIT			ELIT_LOG			ELIT_PIECE			ELIT_SINE			ELIT_SINGER			ELIT_SINU			ELIT_TENT			ELIT_CIRCLE		
		best	median	std	best	median	std	best	median	std	best	median	std	best	median	std	best	median	std	best	median	std	best	median	std
u41	38	40	41	0.76	39	41	0.88	39	41	0.86	38	41	1.11	39	40	0.57	38	40	0.51	40	41	0.84	40	42	0.83
u51	34	36	37	0.6	36	37	0.6	36	37	0.68	36	37	0.88	35	36	0.5	35	35	0.5	35	37	0.69	36	38	0.76
u61	21	21	22	0.58	21	22	0.63	21	22	0.56	21	22	0.64	21	21	0.5	21	21	0.49	21	22	0.56	22	23	0.64
uclr10	25	25	25	0.92	25	25	1.25	25	25	1.1	25	26	1.11	25	27	1.12	27	29	0.67	25	25	1.22	25	27	1.26
uclr11	23	23	26	1.81	23	27	2.01	23	26	1.86	23	27	2.12	23	23	0.5	24	25	1.14	23	25	2.03	23	28	1.38
uclr12	23	23	28	1.7	23	26	1.96	23	28	1.82	23	28	1.76	25	26	0.9	28	28	0.48	23	28	2.02	26	29	0.72
uclr13	23	23	29	1.69	23	29	1.9	23	29	1.55	27	29	0.75	27	30	0.96	29	31	0.7	23	29	1.38	28	29	0.65
ucyc06	60	60	62	0.97	60	62	0.95	60	62	0.92	60	62	0.87	60	62	0.72	60	61	0.91	60	62	1.12	61	63	0.67
ucyc07	144	150	153	1.6	144	153	2.19	148	153	1.83	147	153	1.86	144	152	1.9	144	153	1.96	144	153	2.04	152	156	2.37
ud1	24	25	26	0.43	25	26	0.43	26	26	0.4	25	27	0.67	25	25	0.0	25	25	0.4	26	26	0.4	26	27	0.81
unrf1	10	10	11	0.49	10	11	0.51	10	11	0.5	10	11	0.43	10	11	0.48	10	11	0.48	10	11	0.51	10	11	0.18
**SCA**																									
Instance	Opt	ELIT			ELIT_LOG			ELIT_PIECE			ELIT_SINE			ELIT_SINGER			ELIT_SINU			ELIT_TENT			ELIT_CIRCLE		
		best	median	std	best	median	std	best	median	std	best	median	std	best	median	std	best	median	std	best	median	std	best	median	std
u41	38	40	41	0.76	39	41	0.79	39	41	0.95	39	41	0.75	39	40	0.79	39	40	0.54	40	41	0.93	40	42	0.91
u51	34	35	37	0.91	36	37	0.64	35	37	0.63	36	37	0.87	35	36	0.55	35	36	0.5	36	37	0.58	36	38	0.97
u61	21	21	22	0.51	21	22	0.31	21	22	0.6	21	22	0.53	21	22	0.51	21	21	0.4	21	22	0.48	22	22	0.63
uclr10	25	25	26	1.22	25	27	1.12	25	26	1.08	25	26	1.28	25	26	1.0	26	27	0.69	25	26	1.19	25	27	1.38
uclr11	23	23	27	1.76	23	27	1.78	23	27	1.77	23	27	1.48	23	24	1.56	23	25	1.14	23	27	1.6	23	27	1.72
uclr12	23	24	28	1.05	23	28	1.3	23	28	1.34	26	29	0.89	24	28	1.18	26	28	0.81	25	29	0.97	23	29	1.26
uclr13	23	29	30	0.5	28	30	0.57	27	30	0.68	28	30	0.62	27	30	0.77	27	29	1.11	29	30	0.45	29	30	0.67
ucyc06	60	60	62	1.05	60	62	1.02	60	62	0.81	60	62	0.9	60	62	0.94	60	62	0.78	60	62	0.76	60	63	1.31
ucyc07	144	151	154	1.32	148	154	2.2	146	153	2.11	150	153	2.01	151	154	1.31	152	154	1.33	148	154	1.89	150	155	2.13
ud1	24	25	26	0.45	25	26	0.51	25	26	0.45	25	26	0.58	25	25	0.43	25	25	0.25	25	26	0.37	26	28	1.0
unrf1	10	10	11	0.5	10	11	0.5	10	11	0.49	10	11	0.37	10	10	0.46	10	11	0.51	10	11	0.5	10	11	0.43
**WOA**																									
Instance	Opt	ELIT			ELIT_LOG			ELIT_PIECE			ELIT_SINE			ELIT_SINGER			ELIT_SINU			ELIT_TENT			ELIT_CIRCLE		
		best	median	std	best	median	std	best	median	std	best	median	std	best	median	std	best	median	std	best	median	std	best	median	std
u41	38	40	42	1.28	39	42	1.22	41	43	0.88	40	42	1.07	39	40	0.83	39	40	0.63	40	42	0.99	43	45	1.18
u51	34	36	38	1.0	36	38	0.85	36	38	1.24	36	38	0.88	35	36	0.64	35	36	0.68	36	38	0.96	37	40	1.34
u61	21	22	23	0.7	22	23	0.5	21	23	0.65	22	23	0.67	21	22	0.61	21	22	0.51	22	23	0.6	22	24	0.95
uclr10	25	25	28	1.22	25	27	1.34	25	27	1.34	25	27	1.31	25	26	1.26	25	26	1.1	25	27	1.3	26	28	0.99
uclr11	23	23	28	1.42	23	27	1.91	24	28	1.02	23	27	1.47	23	24	2.07	23	23	1.89	23	28	1.64	24	29	1.43
uclr12	23	23	29	1.34	23	29	1.59	28	29	0.6	26	29	0.87	24	28	1.75	25	26	1.54	27	29	0.65	27	30	0.92
uclr13	23	28	30	0.7	23	30	1.79	27	30	0.93	28	30	0.76	26	30	1.13	27	30	1.06	28	30	0.71	29	31	0.85
ucyc06	60	60	62	0.92	60	62	1.17	60	62	0.83	60	62	0.82	60	62	0.99	60	62	0.85	60	62	1.06	60	65	1.8
ucyc07	144	152	156	1.69	149	154	2.2	151	155	2.36	152	155	1.91	151	154	1.63	144	153	3.02	149	155	2.17	154	160	2.81
ud1	24	26	27	0.66	25	27	0.72	26	27	0.68	25	27	0.82	25	26	0.51	25	25	0.51	26	27	0.85	26	29	1.13
unrf1	10	11	11	0.0	10	11	0.25	10	11	0.18	11	11	0.0	10	11	0.5	10	10	0.51	10	11	0.25	11	11	0.0

**Table A9:** Statistical performance summary of the proposed chaotic binarization framework for the Unicost Set Covering Problem (USCP) instances. Columns report the known global optimum (Opt), best fitness achieved, median, and standard deviation (std) calculated over 31 independent runs for the GWO, SCA, and WOA metaheuristics under the Standard (STD) rule across different chaotic map configurations.

**GWO**																									
Instance	Opt	STD			STD_LOG			STD_PIECE			STD_SINE			STD_SINGER			STD_SINU			STD_TENT			STD_CIRCLE		
		best	median	std	best	median	std	best	median	std	best	median	std	best	median	std	best	median	std	best	median	std	best	median	std
u41	38	46	49	1.58	55	57	1.11	45	50	1.91	57	62	2.18	39	41	0.92	39	40	0.49	45	49	1.65	42	44	1.48
u51	34	41	45	1.74	50	52	1.3	41	46	1.69	54	58	1.65	35	38	0.83	35	35	0.44	42	44	1.29	38	43	2.43
u61	21	25	27	1.05	26	30	1.08	25	27	0.89	29	32	1.31	21	22	0.66	21	21	0.0	24	26	0.81	22	24	1.23
uclr10	25	31	33	0.72	33	34	0.75	30	32	0.84	34	36	0.79	28	29	0.6	27	28	0.65	31	32	0.6	25	29	1.56
uclr11	23	31	32	0.67	32	35	1.11	31	32	0.85	35	36	1.09	27	29	0.58	25	25	0.75	30	32	0.84	30	32	0.75
uclr12	23	32	34	0.8	35	36	0.9	33	34	0.82	35	38	1.15	29	30	0.51	26	28	0.89	31	33	0.8	32	33	0.73
uclr13	23	33	35	0.82	35	38	0.81	33	35	0.83	37	40	1.36	30	31	0.41	27	29	1.11	32	35	0.89	33	34	0.71
ucyc06	60	64	70	2.11	72	75	1.38	66	69	1.61	76	78	1.29	64	65	0.55	60	62	0.52	64	69	1.82	62	65	1.45
ucyc07	144	176	183	2.81	185	193	2.28	181	183	1.56	195	201	2.3	160	165	1.46	151	154	1.18	177	182	1.94	157	162	3.26
ud1	24	31	33	0.93	32	36	1.31	32	33	0.78	36	39	1.46	26	27	0.55	25	25	0.0	31	33	0.88	29	32	1.12
unrf1	10	11	12	0.5	11	12	0.36	11	12	0.46	11	13	0.48	10	11	0.44	10	10	0.0	11	12	0.51	11	12	0.43
**SCA**																									
Instance	Opt	STD			STD_LOG			STD_PIECE			STD_SINE			STD_SINGER			STD_SINU			STD_TENT			STD_CIRCLE		
		best	median	std	best	median	std	best	median	std	best	median	std	best	median	std	best	median	std	best	median	std	best	median	std
u41	38	42	45	1.49	41	45	1.55	41	45	1.47	41	46	1.64	40	42	0.89	40	41	0.64	41	45	1.59	43	45	1.38
u51	34	37	41	1.53	38	41	1.15	38	41	1.37	38	41	1.41	37	38	0.64	36	37	0.51	38	40	1.23	39	42	1.81
u61	21	22	25	1.06	22	24	0.75	23	24	0.81	23	24	0.77	22	23	0.49	21	22	0.18	23	25	0.85	23	24	0.88
uclr10	25	29	30	0.7	29	30	0.63	28	30	0.72	28	31	0.99	27	29	0.85	27	29	0.62	28	30	0.8	28	30	0.79
uclr11	23	28	30	0.77	29	30	0.73	28	30	0.56	29	30	0.61	27	29	0.51	25	28	0.91	29	30	0.63	28	30	0.79
uclr12	23	30	32	0.62	30	31	0.69	30	32	0.62	30	32	0.64	28	30	0.55	28	29	0.57	30	32	0.68	30	32	0.79
uclr13	23	30	33	0.76	31	32	0.6	32	33	0.57	31	33	0.78	30	31	0.41	29	30	0.59	31	32	0.61	32	34	0.77
ucyc06	60	63	65	1.04	63	66	1.37	63	66	1.33	62	66	1.57	63	64	0.65	61	63	0.7	63	65	1.08	62	64	1.63
ucyc07	144	159	165	2.44	164	166	1.97	162	166	1.99	163	169	2.72	158	162	1.41	157	159	0.97	161	165	1.95	161	165	2.0
ud1	24	29	30	0.62	28	29	0.98	29	30	0.89	28	31	0.89	26	27	0.54	26	26	0.37	28	30	0.77	30	32	1.17
unrf1	10	11	11	0.51	11	11	0.4	11	11	0.5	11	11	0.48	10	11	0.3	10	11	0.5	11	11	0.46	11	11	0.51
**WOA**																									
Instance	Opt	STD			STD_LOG			STD_PIECE			STD_SINE			STD_SINGER			STD_SINU			STD_TENT			STD_CIRCLE		
		best	median	std	best	median	std	best	median	std	best	median	std	best	median	std	best	median	std	best	median	std	best	median	std
u41	38	41	43	1.13	40	43	1.13	41	43	1.26	41	43	1.26	39	41	0.91	38	40	0.73	41	44	1.21	43	46	1.68
u51	34	37	39	0.91	36	39	1.38	37	39	0.99	37	39	1.0	36	37	0.82	35	36	0.54	37	39	1.4	38	41	1.38
u61	21	22	24	0.72	21	23	0.67	22	24	0.79	22	24	0.79	21	22	0.53	21	22	0.44	22	23	0.75	24	25	0.85
uclr10	25	25	28	1.26	26	29	1.18	25	28	1.07	25	29	1.25	25	28	1.12	25	27	1.18	25	28	1.14	28	29	0.81
uclr11	23	24	28	1.2	27	29	0.67	27	28	0.66	26	29	0.99	23	28	1.51	23	26	1.9	27	28	0.75	28	29	0.87
uclr12	23	25	30	1.17	28	30	0.71	28	30	0.87	28	30	0.85	28	29	0.61	23	28	1.75	28	30	0.76	28	31	0.81
uclr13	23	30	31	0.63	28	31	0.9	29	31	0.65	29	31	0.84	29	30	0.64	24	29	1.46	30	31	0.6	29	32	1.03
ucyc06	60	60	62	1.11	60	63	1.08	60	63	0.99	62	63	1.1	60	62	1.17	60	62	0.84	60	62	1.17	63	66	1.52
ucyc07	144	153	158	2.21	156	160	2.0	153	157	2.26	155	161	2.72	155	158	1.75	144	154	3.21	153	157	1.68	158	162	2.46
ud1	24	27	28	0.65	27	28	0.73	27	29	0.98	27	29	0.73	26	26	0.51	25	25	0.5	28	28	0.51	26	28	1.57
unrf1	10	11	11	0.34	11	11	0.18	11	11	0.37	11	11	0.44	10	11	0.3	10	11	0.51	11	11	0.18	11	11	0.18

## Appendix C. Computational Efficiency Analysis

This appendix provides a comprehensive computational runtime analysis to supplement the theoretical complexity discussion presented in Section 5.3. To empirically validate the efficiency of the proposed chaotic binarization framework, the following tables report the mean total wall-clock execution time and the standard deviation (std) in seconds, calculated over 31 independent experimental runs for the GWO, SCA, and WOA metaheuristics. The data is organized by problem domain and binarization rule: Table A10, Table A11 and Table A12 detail the execution times for the 0–1 Knapsack Problem (KP); Table A13, Table A14 and Table A15 present the findings for the Set Covering Problem (SCP); and Table A16, Table A17 and Table A18 contain the runtime data for the Unicost Set Covering Problem (USCP) configurations.

**Table A10:** Computational efficiency summary of the proposed chaotic binarization framework for the 0–1 Knapsack Problem (KP) instances. Columns report the mean execution time and standard deviation (std) in seconds, calculated over 31 independent runs for the GWO, SCA, and WOA metaheuristics under the Complement (COM) rule across different chaotic map configurations.

**GWO**																
Instance	COM		COM_LOG		COM_PIECE		COM_SINE		COM_SINGER		COM_SINU		COM_TENT		COM_CIRCLE	
	avg	std	avg	std	avg	std	avg	std	avg	std	avg	std	avg	std	avg	std
1_1000	63.06	15.87	62.84	17.54	62.98	18.56	63.64	19.31	59.99	18.24	55.74	15.67	62.62	15.84	64.55	16.32
1_100	7.5	1.4	7.49	1.48	7.51	1.57	7.58	1.56	7.06	1.29	6.92	1.22	7.19	1.48	7.23	1.37
1_2000	126.03	33.81	126.52	35.25	124.87	36.89	124.18	39.02	114.59	34.3	106.67	32.47	122.95	35.89	127.18	37.33
1_200	11.64	3.59	11.58	3.54	11.64	3.71	11.74	3.62	11.33	4.21	10.66	3.92	12.11	4.25	13.32	4.1
1_500	28.93	8.21	28.89	8.43	28.98	8.48	29.36	8.66	27.64	7.89	26.14	7.71	29.33	8.38	30.13	8.49
2_1000	63.27	17.25	62.94	16.17	62.91	16.15	63.75	16.22	59.67	15.23	56.88	14.21	63.42	14.92	64.5	15.37
2_100	6.59	1.7	6.44	1.64	6.55	1.64	6.73	1.63	5.95	1.67	5.77	1.53	6.55	1.68	6.86	1.62
2_2000	123.67	38.23	123.56	39.47	124.0	39.89	125.79	40.36	115.98	36.13	106.8	31.3	123.67	35.07	126.9	33.82
2_200	13.08	3.06	13.07	3.16	13.02	3.17	13.29	3.3	12.46	3.02	11.81	3.04	13.2	3.22	13.67	3.4
2_500	29.18	8.51	29.15	8.58	29.14	8.5	29.52	8.65	27.81	8.16	26.1	7.47	29.55	8.66	30.34	8.66
3_1000	59.25	19.63	59.1	19.7	59.14	19.62	60.08	20.24	56.36	19.03	52.98	17.95	59.32	18.25	61.06	18.5
3_100	6.76	1.53	6.73	1.49	7.02	1.45	7.45	1.49	6.67	1.42	6.26	1.35	7.0	1.51	7.2	1.47
3_2000	126.0	35.0	125.1	33.92	126.11	34.99	127.3	34.65	118.89	33.21	110.76	31.94	127.04	33.37	131.32	35.56
3_200	12.17	3.13	12.12	3.15	12.1	3.05	12.18	3.27	11.42	3.7	10.91	3.49	12.02	3.86	12.32	3.88
3_500	31.25	7.17	31.33	7.25	31.72	7.83	32.27	8.12	29.65	7.89	27.69	7.57	30.94	8.51	31.42	8.8
**SCA**																
Instance	COM		COM_LOG		COM_PIECE		COM_SINE		COM_SINGER		COM_SINU		COM_TENT		COM_CIRCLE	
	avg	std	avg	std	avg	std	avg	std	avg	std	avg	std	avg	std	avg	std
1_1000	53.75	13.26	53.86	13.7	53.95	13.69	54.97	13.7	51.24	13.28	48.74	12.49	55.36	14.46	56.91	14.06
1_100	6.03	1.41	5.93	1.29	5.99	1.3	6.11	1.4	5.8	1.21	5.44	1.19	6.04	1.39	6.23	1.33
1_2000	104.62	27.96	104.56	29.22	106.01	32.95	107.75	32.15	98.83	29.44	90.96	27.23	106.23	28.49	110.59	30.09
1_200	10.98	2.85	10.81	2.88	10.51	3.05	10.59	2.98	9.82	2.97	9.17	2.74	10.37	3.2	10.68	3.22
1_500	27.81	7.0	27.67	7.13	27.83	7.12	28.04	7.01	26.41	6.78	24.63	6.28	28.17	7.18	28.95	7.19
2_1000	53.9	12.68	53.65	12.84	52.15	14.16	53.08	14.56	49.3	13.37	45.7	12.11	53.07	13.8	55.04	14.57
2_100	5.73	1.18	5.67	1.27	5.66	1.19	5.87	1.42	5.41	1.2	5.08	1.37	6.27	2.04	6.43	2.07
2_2000	111.18	27.55	111.41	29.33	111.5	29.15	113.43	29.08	104.31	28.0	95.8	26.19	112.72	28.12	116.58	27.44
2_200	10.36	3.09	10.18	2.7	10.15	2.67	10.34	2.78	9.82	2.89	9.16	2.59	10.47	3.17	10.69	3.07
2_500	25.54	7.33	25.38	7.28	25.43	7.25	25.83	7.35	24.16	7.05	22.53	6.62	25.8	7.33	26.67	7.49
3_1000	51.52	15.67	51.51	16.35	51.67	16.35	52.23	16.01	48.35	14.2	44.83	12.87	51.69	14.55	52.57	12.25
3_100	5.67	1.34	5.62	1.31	5.64	1.33	5.7	1.4	5.38	1.3	5.02	1.27	5.69	1.34	5.93	1.41
3_2000	110.28	28.91	109.91	29.0	110.09	28.83	112.14	29.1	103.14	27.96	95.55	26.8	111.96	29.88	115.59	29.37
3_200	10.2	3.13	10.14	3.1	10.1	2.94	10.23	2.93	9.64	2.72	9.05	2.54	10.22	2.86	10.55	3.03
3_500	25.39	7.0	25.3	7.03	25.39	7.07	25.75	7.12	24.03	6.71	22.5	6.31	25.73	7.21	26.5	7.15
**WOA**																
Instance	COM		COM_LOG		COM_PIECE		COM_SINE		COM_SINGER		COM_SINU		COM_TENT		COM_CIRCLE	
	avg	std	avg	std	avg	std	avg	std	avg	std	avg	std	avg	std	avg	std
1_1000	58.77	14.73	60.3	15.64	60.0	14.4	58.84	13.52	54.95	12.91	51.64	12.33	58.73	13.85	60.86	15.01
1_100	5.74	1.48	5.82	1.54	5.83	1.47	5.91	1.73	5.82	1.59	5.7	1.49	6.6	1.46	6.7	1.51
1_2000	112.39	31.82	111.82	32.05	111.78	31.01	113.65	31.25	105.26	29.1	97.72	28.58	113.79	32.25	117.74	33.68
1_200	12.22	2.85	12.21	2.99	12.14	2.88	12.36	2.99	11.57	2.73	11.08	2.72	12.32	2.86	12.71	3.13
1_500	26.77	7.61	26.68	7.54	26.77	7.61	27.06	7.52	25.61	7.76	24.36	8.0	27.42	8.66	28.11	8.51
2_1000	57.83	13.69	57.57	13.69	57.9	13.64	58.43	13.68	54.7	12.88	51.37	12.53	58.92	15.14	60.7	14.36
2_100	6.33	1.47	6.21	1.51	6.38	1.43	6.33	1.54	6.07	1.38	5.86	1.33	6.52	1.4	6.57	1.52
2_2000	119.2	29.62	117.54	27.48	119.69	33.25	120.76	31.95	111.32	28.74	102.56	26.38	119.77	29.55	123.93	30.64
2_200	11.57	2.99	11.55	3.16	11.55	3.06	11.57	3.28	10.6	2.86	10.1	2.8	11.27	3.16	11.59	3.33
2_500	27.18	7.47	27.2	8.08	27.44	8.52	27.77	8.65	26.02	8.06	24.43	7.71	27.77	8.63	28.57	8.57
**WOA**																
Instance	COM		COM_LOG		COM_PIECE		COM_SINE		COM_SINGER		COM_SINU		COM_TENT		COM_CIRCLE	
	avg	std	avg	std	avg	std	avg	std	avg	std	avg	std	avg	std	avg	std
3_1000	54.22	13.95	54.22	14.71	54.64	16.64	56.02	18.02	52.56	17.08	49.15	16.09	55.55	16.85	57.1	16.62
3_100	6.38	1.3	6.34	1.3	6.36	1.29	6.42	1.32	6.05	1.31	5.79	1.24	6.43	1.34	6.61	1.39
3_2000	117.72	30.34	117.26	30.04	117.84	31.38	119.63	30.85	110.66	28.29	102.22	25.93	119.21	31.46	121.9	27.44
3_200	10.77	2.91	10.73	3.01	10.81	3.2	10.92	3.13	10.26	2.8	9.72	2.71	10.89	3.08	11.21	3.14
3_500	28.01	7.93	27.96	8.05	27.33	8.22	27.67	8.14	26.02	7.64	24.55	7.39	27.7	8.44	28.45	8.24

**Table A11:** Computational efficiency summary of the proposed chaotic binarization framework for the 0–1 Knapsack Problem (KP) instances. Columns report the mean execution time and standard deviation (std) in seconds, calculated over 31 independent runs for the GWO, SCA, and WOA metaheuristics under the Elitist (ELIT) rule across different chaotic map configurations.

**GWO**																
Instance	ELIT		ELIT_LOG		ELIT_PIECE		ELIT_SINE		ELIT_SINGER		ELIT_SINU		ELIT_TENT		ELIT_CIRCLE	
	avg	std	avg	std	avg	std	avg	std	avg	std	avg	std	avg	std	avg	std
1_1000	54.53	15.18	54.77	15.92	55.71	14.62	55.11	15.82	54.59	15.64	54.87	15.35	55.26	15.56	55.4	16.06
1_100	6.14	1.36	6.64	1.11	6.01	1.42	5.96	1.28	5.89	1.46	5.69	1.39	5.76	1.58	5.77	1.55
1_2000	104.67	31.78	105.01	32.27	105.16	33.07	105.28	32.73	103.97	29.54	103.72	27.52	104.39	27.42	104.23	27.31
1_200	11.69	2.81	11.89	2.97	11.63	2.86	11.66	3.06	11.54	2.83	11.66	2.99	11.71	2.93	11.74	3.07
1_500	25.54	7.49	25.66	7.84	25.77	8.05	25.8	8.06	25.67	7.82	25.67	7.46	25.81	7.61	25.82	7.79
2_1000	55.01	13.54	55.36	13.5	54.37	14.74	54.56	15.33	54.6	15.71	54.79	15.73	55.3	16.42	55.34	16.98
2_100	5.73	1.55	5.71	1.46	5.88	1.5	5.97	1.45	5.91	1.48	5.91	1.43	6.01	1.53	6.03	1.57
2_2000	104.62	31.05	105.79	35.15	105.46	33.17	104.5	30.14	106.98	29.42	109.35	32.22	109.16	30.42	108.52	28.46
2_200	11.7	2.93	11.63	2.94	11.6	2.8	11.58	2.86	11.53	2.73	11.62	2.83	11.71	2.7	11.66	2.76
2_500	25.64	7.46	25.55	7.36	25.65	7.79	25.67	7.68	25.53	7.35	25.74	7.58	25.91	7.85	25.84	7.72
3_1000	51.68	16.5	51.68	17.1	51.34	15.7	51.51	16.03	51.64	16.62	51.89	16.82	52.2	16.91	51.82	16.21
3_100	6.15	1.31	6.14	1.35	6.12	1.44	6.09	1.4	6.02	1.35	6.08	1.35	6.14	1.33	6.14	1.38
3_2000	108.65	31.41	108.55	31.52	109.24	32.9	108.57	30.17	108.38	30.78	109.73	32.39	109.18	30.35	109.85	32.1
3_200	10.8	3.4	10.78	3.45	10.72	3.4	10.68	3.32	10.58	3.22	10.66	3.29	10.75	3.32	10.76	3.34
3_500	26.6	7.61	26.6	7.83	26.54	7.55	26.58	7.3	26.43	7.28	26.67	7.64	26.82	7.62	26.76	7.64
**SCA**																
Instance	ELIT		ELIT_LOG		ELIT_PIECE		ELIT_SINE		ELIT_SINGER		ELIT_SINU		ELIT_TENT		ELIT_CIRCLE	
	avg	std	avg	std	avg	std	avg	std	avg	std	avg	std	avg	std	avg	std
1_1000	46.92	12.23	46.5	11.91	46.9	12.12	46.87	12.25	46.94	12.79	47.05	12.16	47.14	11.81	47.3	11.92
1_100	5.09	1.21	5.16	1.23	5.3	1.14	5.11	1.18	4.84	1.4	5.14	1.51	5.54	1.69	5.57	1.88
1_2000	89.27	26.46	88.71	26.35	88.94	26.39	88.29	23.99	87.7	22.61	88.24	22.79	88.72	22.45	88.57	22.73
1_200	8.93	2.66	8.89	2.69	8.9	2.69	8.89	2.61	8.92	2.75	8.95	2.66	9.03	2.74	8.99	2.73
1_500	24.26	6.13	24.13	6.17	24.19	6.12	24.22	6.03	24.12	5.98	24.15	5.75	24.32	5.66	24.19	5.57
2_1000	44.83	12.63	44.65	12.48	44.75	12.5	44.95	12.83	45.14	13.8	45.6	14.15	45.65	13.6	45.7	13.58
2_100	5.42	1.83	5.41	1.76	5.3	1.22	5.26	1.1	5.28	1.17	5.29	1.16	5.24	1.21	4.85	1.31
2_2000	92.5	23.22	93.18	25.06	93.45	25.86	93.49	25.5	93.21	25.44	94.04	25.95	93.52	23.19	94.55	25.73
2_200	9.02	2.56	8.95	2.57	9.01	2.77	8.97	2.59	8.95	2.67	9.0	2.55	9.12	2.65	9.03	2.61
2_500	22.07	6.55	22.01	6.62	22.02	6.57	22.09	6.67	22.03	6.68	22.17	6.69	22.28	6.67	22.28	6.87
3_1000	44.12	12.56	43.69	12.18	43.89	12.47	43.78	11.87	44.0	12.68	45.46	11.99	48.34	10.31	47.99	10.67
3_100	4.92	1.22	4.75	1.28	4.72	1.28	4.73	1.23	4.65	1.14	4.69	1.17	4.74	1.15	4.71	1.05
3_2000	93.41	26.28	93.61	26.79	93.41	25.99	93.52	25.79	92.9	24.81	94.1	26.74	94.37	26.41	92.11	21.43
3_200	8.97	2.82	8.87	2.65	8.84	2.55	8.86	2.57	8.86	2.61	8.92	2.63	8.96	2.61	9.01	2.82
3_500	22.05	6.21	21.84	6.37	21.86	6.38	21.86	6.25	21.88	6.37	22.01	6.45	22.08	6.35	22.04	6.36
**WOA**																
Instance	ELIT		ELIT_LOG		ELIT_PIECE		ELIT_SINE		ELIT_SINGER		ELIT_SINU		ELIT_TENT		ELIT_CIRCLE	
	avg	std	avg	std	avg	std	avg	std	avg	std	avg	std	avg	std	avg	std
1_1000	50.96	13.54	50.83	13.51	50.86	13.41	50.89	13.24	50.4	12.63	50.33	12.31	50.42	12.07	50.82	13.45
1_100	5.53	1.41	5.53	1.44	5.43	1.44	5.51	1.46	5.66	1.4	5.74	1.38	5.75	1.36	5.87	1.37
1_2000	95.55	27.38	95.34	26.68	95.45	27.04	95.36	26.44	95.77	27.65	96.53	28.78	97.05	29.06	96.35	27.2
1_200	10.92	2.52	10.9	2.56	10.89	2.62	10.88	2.54	10.32	2.81	10.4	2.78	10.5	2.86	10.44	2.84
1_500	23.64	7.06	23.59	7.11	23.68	7.28	23.75	7.44	23.76	7.59	23.91	7.6	24.01	7.64	23.92	7.51
2_1000	50.0	11.53	50.02	12.2	50.38	12.61	50.69	13.37	50.6	14.07	51.26	14.6	51.24	14.23	51.19	13.99
2_100	5.39	1.31	5.38	1.38	5.36	1.36	5.28	1.39	5.23	1.42	5.28	1.41	5.35	1.37	5.36	1.44
2_2000	100.5	27.15	100.95	28.83	101.11	28.25	100.95	27.91	100.83	28.4	101.84	29.31	102.21	29.63	101.48	27.77
2_200	10.0	2.69	9.94	2.7	9.82	2.99	9.76	2.89	9.76	2.99	9.78	2.88	9.89	2.99	9.83	2.96
2_500	24.03	7.44	23.96	7.72	23.85	7.23	23.82	6.93	23.75	6.91	23.87	6.88	23.97	6.96	23.95	7.03
3_1000	48.21	15.37	48.12	15.86	48.12	15.71	47.64	13.98	47.52	13.76	47.74	13.71	47.94	13.68	47.91	13.95
3_100	5.74	1.21	5.65	1.23	5.69	1.18	5.67	1.21	5.53	1.26	5.65	1.23	5.75	1.17	5.77	1.23
3_2000	100.05	25.28	100.18	26.65	100.91	28.27	101.01	27.93	100.59	27.46	101.0	27.19	101.9	27.67	101.69	27.92
3_200	9.54	2.58	9.56	2.73	9.63	2.9	9.69	3.05	9.68	3.07	9.73	3.08	9.77	3.04	9.77	3.1
3_500	23.93	6.82	23.79	6.61	23.78	6.57	23.99	6.96	24.04	7.2	24.1	7.09	24.22	7.0	24.16	6.95

**Table A12:** Computational efficiency summary of the proposed chaotic binarization framework for the 0–1 Knapsack Problem (KP) instances. Columns report the mean execution time and standard deviation (std) in seconds, calculated over 31 independent runs for the GWO, SCA, and WOA metaheuristics under the Standard (STD) rule across different chaotic map configurations.

**GWO**																
Instance	STD		STD_LOG		STD_PIECE		STD_SINE		STD_SINGER		STD_SINU		STD_TENT		STD_CIRCLE	
	avg	std	avg	std	avg	std	avg	std	avg	std	avg	std	avg	std	avg	std
1_1000	63.28	15.09	63.16	15.32	62.32	15.08	62.28	15.62	57.75	14.94	54.18	14.37	61.0	15.38	62.7	14.92
1_100	6.91	1.56	7.24	1.4	7.2	1.45	7.32	1.43	6.89	1.41	6.54	1.28	7.32	1.4	7.5	1.4
1_2000	127.56	30.37	121.94	34.51	121.14	34.56	122.92	34.69	113.2	30.13	105.02	27.14	124.16	31.47	129.58	33.52
1_200	11.59	4.17	11.54	3.87	11.55	3.75	11.57	3.71	10.82	3.33	10.19	3.03	11.38	3.43	11.69	3.52
1_500	28.33	8.4	28.14	8.33	28.22	8.25	28.62	8.44	26.97	8.0	25.39	7.47	28.46	8.4	29.23	8.69
2_1000	61.44	15.28	61.22	15.99	61.49	16.39	62.05	16.17	57.63	14.03	54.28	13.24	61.64	15.19	64.17	16.91
2_100	6.37	1.63	6.79	1.39	6.44	1.59	6.28	1.68	5.86	1.6	5.72	1.42	6.28	1.69	6.7	1.58
2_2000	124.8	30.7	122.44	33.89	119.62	35.07	120.88	34.97	112.35	33.5	104.08	31.3	120.89	36.02	125.49	37.76
2_200	12.55	3.22	12.71	3.07	12.78	3.22	12.86	3.25	12.19	3.12	11.51	2.89	12.94	3.16	13.23	3.28
2_500	28.42	8.09	28.28	8.3	28.32	8.24	28.61	8.15	26.97	7.84	25.36	7.52	28.54	8.05	29.48	8.51
3_1000	57.43	17.75	57.03	17.89	57.27	18.18	58.01	18.6	54.72	18.27	51.39	17.23	58.27	19.53	60.2	20.36
3_100	6.4	1.43	6.52	1.39	6.55	1.47	6.63	1.5	6.34	1.38	6.03	1.32	6.65	1.5	6.81	1.52
3_2000	126.39	33.45	125.33	32.15	125.47	32.5	127.27	31.4	118.99	30.19	111.09	28.73	128.66	35.19	129.86	35.98
3_200	12.39	2.85	12.37	2.84	11.84	3.07	11.91	2.93	11.33	2.97	10.85	2.76	11.98	3.12	12.22	3.06
3_500	28.24	8.52	28.06	8.57	28.19	8.63	28.57	8.86	26.97	8.41	25.45	7.93	28.25	8.62	30.14	8.01
**SCA**																
Instance	STD		STD_LOG		STD_PIECE		STD_SINE		STD_SINGER		STD_SINU		STD_TENT		STD_CIRCLE	
	avg	std	avg	std	avg	std	avg	std	avg	std	avg	std	avg	std	avg	std
1_1000	52.63	13.13	51.89	12.45	51.98	12.33	52.96	12.77	49.32	11.95	45.68	10.69	52.67	12.56	54.86	13.27
1_100	5.89	1.29	5.87	1.3	5.87	1.31	5.91	1.19	5.62	1.18	5.36	1.28	5.96	1.45	6.05	1.47
1_2000	101.16	25.01	100.28	24.41	100.91	24.86	102.4	24.99	94.63	24.68	87.35	23.4	102.87	27.38	106.95	28.39
1_200	10.72	2.67	10.62	2.73	10.64	2.77	10.74	2.77	10.07	2.65	9.32	2.51	10.81	2.82	11.02	2.9
1_500	24.62	7.57	24.4	7.42	25.87	7.01	27.14	6.53	25.53	6.31	23.91	5.93	27.22	6.69	28.04	6.87
2_1000	52.93	13.04	52.73	13.1	52.71	12.67	53.32	12.48	49.5	11.85	46.36	11.6	53.44	13.04	55.2	13.13
2_100	5.89	1.23	5.73	1.31	5.54	1.28	5.62	1.36	5.23	1.26	4.77	1.27	5.59	1.27	5.78	1.28
2_2000	108.19	26.82	108.18	28.63	107.86	27.52	109.31	26.93	100.41	25.4	92.45	23.9	109.45	27.65	113.3	27.67
2_200	10.11	2.92	10.01	2.89	10.01	2.99	10.07	2.84	9.53	2.75	8.8	2.6	10.15	3.01	10.43	3.1
2_500	24.66	6.79	24.5	6.89	24.56	6.85	24.91	7.03	23.23	6.62	21.63	6.2	24.91	7.02	25.82	7.37
3_1000	49.44	13.15	49.3	14.1	50.03	15.85	50.66	15.91	46.99	14.55	43.76	13.49	50.45	15.28	52.31	15.78
3_100	5.83	1.15	5.65	1.15	5.48	1.12	5.58	1.3	5.2	1.27	4.78	1.25	5.55	1.29	5.66	1.36
3_2000	107.7	27.0	107.04	28.51	107.84	29.42	108.32	27.27	100.48	27.25	92.67	25.91	108.86	29.74	112.83	29.79
3_200	9.94	3.02	9.88	3.0	9.88	3.03	10.02	3.1	9.43	2.88	8.82	2.77	10.0	3.09	10.31	3.18
3_500	24.67	6.66	24.49	6.83	24.59	6.96	24.95	7.09	23.34	6.64	21.82	6.24	24.87	6.94	25.66	7.07
**WOA**																
Instance	STD		STD_LOG		STD_PIECE		STD_SINE		STD_SINGER		STD_SINU		STD_TENT		STD_CIRCLE	
	avg	std	avg	std	avg	std	avg	std	avg	std	avg	std	avg	std	avg	std
1_1000	57.13	14.7	56.63	15.01	56.79	14.99	57.58	15.43	54.06	14.94	50.55	14.23	57.24	14.42	59.11	14.65
1_100	5.79	1.52	5.77	1.48	5.8	1.5	5.83	1.47	5.69	1.29	5.51	1.16	5.75	1.43	5.83	1.56
1_2000	110.11	27.29	109.47	27.89	110.03	29.79	111.08	32.31	102.9	31.57	95.12	29.72	111.38	34.53	115.01	34.41
1_200	11.91	2.76	11.88	2.93	11.86	2.78	12.04	2.99	11.3	2.67	10.76	2.64	12.01	2.95	12.34	3.07
1_500	26.16	7.2	26.09	7.3	26.15	7.31	26.42	7.34	24.87	7.03	23.32	6.6	26.55	7.37	26.93	7.57
2_1000	57.31	15.88	56.94	16.06	57.39	16.76	57.93	16.48	53.78	15.14	50.28	13.83	56.9	13.61	58.86	13.7
2_100	5.76	1.62	5.7	1.61	5.68	1.59	5.69	1.52	5.45	1.58	5.03	1.39	5.77	1.6	6.2	1.53
2_2000	114.83	26.85	114.78	28.25	114.7	27.88	115.95	26.66	108.03	28.73	99.55	26.69	117.29	32.08	121.41	32.19
2_200	11.89	2.45	11.83	2.68	11.55	2.72	11.39	2.97	10.67	2.57	10.14	2.49	11.33	2.81	11.68	3.09
2_500	26.61	7.87	26.55	8.26	26.55	8.17	26.73	7.61	24.89	6.82	23.39	6.69	26.76	7.64	27.51	7.76
3_1000	53.26	14.95	52.9	15.31	52.96	15.04	53.72	15.35	50.36	15.4	47.43	15.58	54.18	16.91	55.06	14.59
3_100	6.25	1.24	6.08	1.29	6.21	1.28	6.33	1.28	5.96	1.22	5.67	1.19	6.27	1.27	6.46	1.31
3_2000	115.07	30.14	114.7	30.69	114.97	31.04	116.44	31.27	107.56	28.95	99.03	25.9	116.75	32.58	120.45	32.12
3_200	10.87	3.12	10.8	3.22	10.75	3.03	10.79	2.87	10.16	2.65	9.67	2.54	10.82	2.94	10.92	3.06
3_500	27.09	6.68	26.91	6.88	27.03	6.99	27.35	7.05	25.85	7.18	24.23	6.73	27.58	7.63	28.35	7.76

**Table A13:** Computational efficiency summary of the proposed chaotic binarization framework for the Set Covering Problem (SCP) instances. Columns report the mean execution time and standard deviation (std) in seconds, calculated over 31 independent runs for the GWO, SCA, and WOA metaheuristics under the Complement (COM) rule across different chaotic map configurations.

**GWO**																
Instance	COM		COM_LOG		COM_PIECE		COM_SINE		COM_SINGER		COM_SINU		COM_TENT		COM_CIRCLE	
	avg	std	avg	std	avg	std	avg	std	avg	std	avg	std	avg	std	avg	std
41	85.65	11.05	92.79	13.19	90.5	14.74	87.65	11.33	91.24	15.52	94.34	16.76	90.16	12.3	84.54	11.42
51	146.24	14.42	146.95	10.24	148.28	10.73	145.28	11.75	149.34	13.59	153.65	12.75	149.69	12.59	148.52	13.12
61	65.39	11.65	62.15	9.37	61.79	9.08	63.88	8.61	64.59	9.38	65.35	7.44	61.75	9.58	63.35	8.44
a1	329.96	22.53	324.9	22.35	322.29	24.87	318.78	20.82	329.95	25.27	341.34	20.3	333.52	27.28	317.4	18.34
b1	201.09	15.26	200.01	19.99	195.72	15.02	194.06	14.41	200.55	16.07	211.16	13.14	195.51	14.36	196.54	14.28
c1	419.37	44.78	421.19	43.84	425.15	36.47	395.79	31.34	419.37	45.84	445.05	37.98	417.55	38.78	405.64	42.47
d1	213.72	25.39	212.12	25.89	222.35	29.36	214.81	29.59	235.67	38.25	242.1	25.71	209.41	24.66	217.69	27.81
nre1	178.12	28.5	170.13	24.5	176.8	25.69	164.51	25.19	193.51	28.42	191.97	31.94	176.17	32.03	166.95	31.58
nrf1	111.52	18.62	104.75	17.3	107.69	13.01	105.31	16.48	121.04	14.65	132.76	20.11	108.42	14.35	103.25	18.32
**SCA**																
Instance	COM		COM_LOG		COM_PIECE		COM_SINE		COM_SINGER		COM_SINU		COM_TENT		COM_CIRCLE	
	avg	std	avg	std	avg	std	avg	std	avg	std	avg	std	avg	std	avg	std
41	84.24	9.31	83.58	8.97	86.67	14.61	84.16	11.49	85.63	9.51	86.03	15.62	84.89	13.13	88.09	11.57
51	161.16	21.88	160.59	28.84	162.12	26.8	166.12	20.37	161.28	22.09	167.21	21.74	165.62	17.52	158.55	17.59
61	63.98	9.4	64.72	12.03	61.69	10.67	64.28	8.15	61.2	10.89	61.36	10.86	61.73	8.39	64.0	9.37
a1	333.01	25.01	333.74	22.07	337.0	26.76	330.56	27.43	336.97	29.24	338.74	30.9	337.36	30.98	329.06	28.65
b1	202.79	18.78	205.92	19.49	203.7	20.53	207.67	18.11	210.53	22.1	210.9	18.4	203.13	17.88	203.36	15.12
c1	441.48	51.99	429.24	56.22	435.06	50.4	438.3	50.45	435.25	48.28	435.23	42.6	416.81	43.41	438.74	45.0
d1	236.97	36.23	244.37	39.45	243.5	28.56	249.04	32.12	252.17	29.76	243.17	26.32	238.26	30.15	233.21	27.75
nre1	179.31	24.45	195.6	32.01	196.78	17.79	198.79	19.35	198.33	21.83	197.32	24.07	195.86	28.04	201.75	35.66
nrf1	129.76	11.06	129.02	10.73	128.94	7.89	128.85	11.65	131.99	9.58	136.06	10.52	129.4	10.62	128.67	10.12
**WOA**																
Instance	COM		COM_LOG		COM_PIECE		COM_SINE		COM_SINGER		COM_SINU		COM_TENT		COM_CIRCLE	
	avg	std	avg	std	avg	std	avg	std	avg	std	avg	std	avg	std	avg	std
41	83.97	11.9	83.67	11.85	86.35	14.09	83.08	12.04	87.79	13.94	86.79	12.98	82.08	12.09	83.07	13.39
51	161.84	23.99	163.83	19.91	165.92	24.31	154.98	17.97	159.02	18.46	164.93	21.54	156.5	12.29	156.3	19.86
61	62.08	8.75	61.31	10.28	57.81	7.74	58.13	7.47	59.91	10.16	59.34	7.61	62.38	10.06	59.93	10.02
a1	337.28	33.4	321.74	25.18	319.51	29.02	326.85	27.55	326.34	23.22	324.6	24.31	326.01	28.12	328.13	22.07
b1	200.17	19.87	202.69	25.38	198.31	20.51	197.25	19.31	196.43	19.5	198.19	19.66	202.11	19.06	197.78	19.02
c1	429.12	52.62	416.73	45.25	407.6	37.89	430.78	51.85	409.95	45.04	428.2	62.96	408.95	49.91	411.46	39.03
d1	222.66	22.4	225.89	31.55	227.84	32.56	227.49	33.52	218.86	27.52	226.71	26.83	212.69	25.33	217.46	31.98
nre1	173.53	29.25	174.07	29.15	178.61	26.42	179.35	32.0	182.27	39.34	180.06	30.33	167.31	24.81	174.07	29.74
nrf1	120.35	9.66	116.83	7.55	114.51	9.32	117.13	8.48	119.32	10.91	121.58	9.32	116.38	9.43	114.8	9.84

**Table A14:** Computational efficiency summary of the proposed chaotic binarization framework for the Set Covering Problem (SCP) instances. Columns report the mean execution time and standard deviation (std) in seconds, calculated over 31 independent runs for the GWO, SCA, and WOA metaheuristics under the Elitist (ELIT) rule across different chaotic map configurations.

**GWO**																
Instance	ELIT		ELIT_LOG		ELIT_PIECE		ELIT_SINE		ELIT_SINGER		ELIT_SINU		ELIT_TENT		ELIT_CIRCLE	
	avg	std	avg	std	avg	std	avg	std	avg	std	avg	std	avg	std	avg	std
41	45.65	5.42	51.27	6.28	49.92	8.89	51.4	7.83	60.62	8.14	62.85	9.19	49.8	7.11	44.98	5.87
51	85.55	8.88	88.07	7.84	84.08	8.07	87.32	7.8	105.59	10.2	111.63	9.63	84.97	8.23	74.73	6.18
61	42.34	6.2	41.4	5.99	41.5	5.75	43.74	6.31	49.08	5.26	52.32	6.63	41.26	6.04	39.77	7.12
a1	150.06	10.94	163.4	13.16	145.44	13.93	153.9	10.83	206.84	19.5	223.4	19.88	147.68	11.04	121.55	9.86
b1	109.63	9.08	118.22	10.62	109.27	8.06	114.89	9.14	140.36	12.65	150.95	13.85	111.26	9.74	96.42	6.1
c1	168.81	27.71	194.34	24.16	172.52	24.77	183.29	22.28	247.32	39.49	284.33	32.74	174.35	22.69	132.04	14.35
d1	115.39	15.2	129.96	18.45	120.49	16.46	127.92	16.85	161.39	19.74	172.8	25.93	120.67	16.54	101.27	12.97
nre1	98.08	15.68	101.09	16.37	95.78	18.24	98.56	18.11	125.69	18.91	135.12	22.2	95.26	16.29	76.78	12.13
nrf1	82.79	14.78	86.96	13.01	80.75	13.5	80.76	13.15	102.31	16.92	110.49	19.55	81.25	14.63	69.68	10.97
**SCA**																
Instance	ELIT		ELIT_LOG		ELIT_PIECE		ELIT_SINE		ELIT_SINGER		ELIT_SINU		ELIT_TENT		ELIT_CIRCLE	
	avg	std	avg	std	avg	std	avg	std	avg	std	avg	std	avg	std	avg	std
41	46.14	7.66	48.04	6.72	48.64	9.49	47.87	7.25	59.11	8.64	62.26	8.72	47.89	7.16	40.91	6.62
51	89.02	11.02	96.82	14.58	88.01	12.87	89.5	12.07	110.96	12.84	119.33	15.67	86.45	11.62	79.69	10.87
61	41.04	8.04	40.83	5.58	40.08	6.2	40.91	5.87	47.89	8.55	49.44	7.81	39.41	6.4	35.68	4.98
a1	151.32	17.04	168.75	13.25	149.3	12.94	158.67	14.26	209.64	19.91	219.57	18.1	149.59	11.57	128.22	12.36
b1	115.67	10.0	119.91	10.07	114.06	9.44	108.92	10.14	141.22	13.55	151.21	14.6	113.98	11.01	101.21	9.99
c1	176.21	25.25	186.15	18.47	176.83	20.51	182.73	27.1	249.29	33.29	260.85	42.12	178.09	27.66	143.43	20.4
**SCA**																
Instance	ELIT		ELIT_LOG		ELIT_PIECE		ELIT_SINE		ELIT_SINGER		ELIT_SINU		ELIT_TENT		ELIT_CIRCLE	
	avg	std	avg	std	avg	std	avg	std	avg	std	avg	std	avg	std	avg	std
d1	118.24	24.81	130.11	19.46	118.12	19.65	122.12	17.66	152.25	29.04	172.06	26.2	128.75	21.71	94.81	13.25
nre1	86.96	10.92	105.9	17.06	92.91	11.27	94.88	12.52	124.89	15.04	129.21	17.04	93.83	14.14	80.52	10.75
nrf1	82.11	6.88	86.22	6.33	80.98	7.84	82.38	6.86	98.23	8.49	107.56	11.14	83.03	8.12	75.68	5.37
**WOA**																
Instance	ELIT		ELIT_LOG		ELIT_PIECE		ELIT_SINE		ELIT_SINGER		ELIT_SINU		ELIT_TENT		ELIT_CIRCLE	
	avg	std	avg	std	avg	std	avg	std	avg	std	avg	std	avg	std	avg	std
41	39.26	6.94	45.25	6.73	40.69	6.09	45.07	6.85	51.02	6.36	54.43	8.43	40.27	5.57	34.81	6.15
51	75.66	10.25	86.72	13.39	75.49	11.77	85.57	14.15	95.85	13.21	101.46	11.93	78.03	12.19	69.19	9.98
61	37.22	5.75	37.81	8.88	36.56	6.31	37.88	5.43	42.67	7.96	41.78	6.16	35.65	6.05	32.74	4.64
a1	125.12	16.27	145.88	14.24	123.6	10.63	136.7	14.88	181.72	21.73	180.55	13.03	129.56	13.95	102.35	9.2
b1	98.91	8.47	103.38	10.29	94.97	11.92	104.58	8.53	122.58	13.02	125.76	13.74	96.72	7.9	86.13	9.09
c1	143.89	18.51	177.01	34.07	133.18	19.03	156.5	29.09	216.41	36.3	213.1	29.13	148.18	19.24	106.05	16.84
d1	97.47	17.55	115.75	14.03	96.98	12.89	105.96	19.26	138.86	22.41	130.75	21.68	93.99	14.68	85.02	15.23
nre1	74.71	9.57	89.47	16.07	76.48	12.58	83.41	15.18	107.53	21.52	102.61	18.33	77.97	12.98	69.08	11.56
nrf1	71.3	5.96	79.0	6.75	69.16	6.77	75.92	5.88	87.06	7.19	87.92	7.05	71.43	6.39	88.49	47.33

**Table A15:** Computational efficiency summary of the proposed chaotic binarization framework for the Set Covering Problem (SCP) instances. Columns report the mean execution time and standard deviation (std) in seconds, calculated over 31 independent runs for the GWO, SCA, and WOA metaheuristics under the Standard (STD) rule across different chaotic map configurations.

**GWO**																
Instance	STD		STD_LOG		STD_PIECE		STD_SINE		STD_SINGER		STD_SINU		STD_TENT		STD_CIRCLE	
	avg	std	avg	std	avg	std	avg	std	avg	std	avg	std	avg	std	avg	std
41	53.5	10.96	48.88	7.0	46.35	7.13	45.33	7.31	60.92	7.67	63.41	7.91	45.85	6.04	39.43	5.14
51	94.82	8.96	82.19	6.53	77.07	6.57	74.83	7.2	101.48	10.11	106.78	9.62	77.51	6.05	67.2	5.78
61	45.7	5.97	40.89	5.32	40.11	5.13	36.86	5.03	45.4	5.76	51.53	7.52	41.03	6.46	33.93	5.92
a1	143.84	11.99	137.74	9.57	126.53	8.72	122.02	8.99	190.6	15.52	216.94	15.74	128.74	10.22	105.86	7.82
b1	116.82	10.32	101.71	6.8	97.9	7.42	96.21	7.88	131.36	11.42	147.89	10.46	99.46	5.47	89.83	5.28
c1	159.27	25.81	151.78	22.72	147.3	18.61	136.58	16.04	225.3	19.07	266.37	26.12	143.75	24.17	94.07	13.85
d1	117.71	12.37	104.08	15.18	97.52	13.55	96.74	13.45	143.72	23.15	167.0	26.59	100.05	16.36	72.24	10.89
nre1	93.8	11.16	85.75	12.0	81.48	7.93	75.27	10.98	119.94	17.84	131.38	15.27	83.25	12.87	67.37	8.66
nrf1	80.76	14.16	62.17	7.34	68.96	12.17	60.78	9.04	91.29	14.57	102.3	14.86	68.26	12.43	60.8	10.05
**SCA**																
Instance	STD		STD_LOG		STD_PIECE		STD_SINE		STD_SINGER		STD_SINU		STD_TENT		STD_CIRCLE	
	avg	std	avg	std	avg	std	avg	std	avg	std	avg	std	avg	std	avg	std
41	53.92	10.14	48.52	6.9	43.32	8.03	44.72	7.24	56.54	8.37	61.08	9.42	42.82	6.84	41.31	6.46
51	94.81	7.65	88.2	12.58	79.76	11.9	83.68	9.7	105.06	14.74	108.63	13.08	80.83	10.15	73.02	7.69
61	41.93	9.28	38.78	7.04	35.99	5.58	38.32	7.41	44.89	8.46	46.57	7.46	37.1	5.11	35.74	5.31
a1	150.36	12.66	152.05	14.68	133.54	10.17	142.24	14.2	183.29	12.79	204.92	22.55	134.25	11.7	118.73	13.02
b1	119.1	9.52	111.03	13.2	102.21	8.46	105.5	10.23	128.06	10.86	140.81	15.32	104.1	10.4	91.54	7.96
c1	190.08	32.29	175.09	32.18	149.87	21.45	161.81	22.79	234.7	29.86	254.38	38.6	152.22	22.68	126.8	24.51
d1	118.31	21.84	110.1	20.23	100.22	15.06	104.32	16.77	133.38	18.58	155.15	25.35	101.86	16.66	91.68	13.67
nre1	89.5	14.57	89.49	11.74	81.72	11.92	86.05	11.02	109.34	13.38	121.66	15.59	86.92	12.94	72.84	8.94
nrf1	86.16	10.26	76.66	7.38	71.37	6.33	72.07	6.79	88.3	7.32	93.66	7.06	73.47	5.51	68.05	5.67
**WOA**																
Instance	STD		STD_LOG		STD_PIECE		STD_SINE		STD_SINGER		STD_SINU		STD_TENT		STD_CIRCLE	
	avg	std	avg	std	avg	std	avg	std	avg	std	avg	std	avg	std	avg	std
41	45.15	9.25	37.88	5.5	35.6	5.48	35.41	5.11	47.35	6.02	49.09	8.92	34.01	5.65	30.16	5.45
51	81.47	12.75	71.45	14.4	63.99	7.66	66.9	9.08	88.51	12.35	95.3	14.08	67.86	12.31	59.74	9.57
61	38.97	8.77	33.2	5.47	30.19	4.92	30.6	5.04	36.64	5.25	37.54	6.42	31.77	4.88	27.9	5.42
a1	114.25	14.24	110.44	9.45	97.64	10.26	103.98	10.5	148.21	15.44	166.52	14.94	101.87	11.47	84.63	8.43
b1	99.06	11.03	85.48	8.34	77.16	9.78	84.25	7.87	102.8	11.52	117.33	11.72	79.45	7.06	76.61	7.62
c1	117.63	19.39	125.27	17.34	108.58	18.03	115.2	17.95	172.8	23.54	197.76	31.21	106.39	16.48	84.1	9.51
d1	88.93	10.96	83.8	15.92	73.15	11.65	79.28	14.45	108.9	14.42	127.09	20.46	75.84	13.18	71.02	9.43
nre1	67.27	9.55	61.53	5.54	58.11	5.31	59.66	4.71	87.86	9.94	100.26	12.19	57.25	5.45	54.27	7.25
nrf1	64.83	7.11	55.59	6.0	52.56	5.95	52.74	5.77	70.92	7.59	78.24	7.11	53.02	4.43	51.34	4.54

**Table A16:** Computational efficiency summary of the proposed chaotic binarization framework for the Unicost Set Covering Problem (USCP) instances. Columns report the mean execution time and standard deviation (std) in seconds, calculated over 31 independent runs for the GWO, SCA, and WOA metaheuristics under the Complement (COM) rule across different chaotic map configurations.

**GWO**																
Instance	COM		COM_LOG		COM_PIECE		COM_SINE		COM_SINGER		COM_SINU		COM_TENT		COM_CIRCLE	
	avg	std	avg	std	avg	std	avg	std	avg	std	avg	std	avg	std	avg	std
u41	40.43	24.53	43.75	23.66	39.76	23.33	42.05	22.13	47.99	26.88	55.58	29.23	41.04	24.18	41.56	23.88
u51	74.87	40.08	81.51	36.31	71.56	40.29	69.24	35.25	84.69	45.09	89.3	45.77	73.12	40.4	76.21	37.08
u61	35.18	18.97	32.18	18.49	35.68	19.64	33.11	18.65	37.5	21.61	40.28	22.33	36.4	19.07	35.3	19.04
uclr10	2.79	0.87	2.64	0.82	2.69	0.83	2.51	0.76	3.25	1.08	3.79	1.2	2.79	0.84	2.63	0.74
uclr11	5.85	1.41	5.6	1.55	5.79	1.51	5.51	1.49	7.03	1.99	8.4	2.25	5.99	1.64	5.79	1.61
uclr12	61.39	37.24	58.17	32.6	63.98	41.1	54.37	31.08	86.86	38.88	112.05	63.64	54.65	37.35	51.2	34.2
uclr13	170.65	94.05	168.77	80.36	179.69	85.12	158.01	75.55	220.05	122.63	290.43	148.53	167.5	87.61	143.8	81.71
ucyc06	3.02	1.12	2.78	0.91	3.05	1.09	2.73	0.85	3.39	1.18	3.93	1.44	2.85	0.89	2.78	0.95
ucyc07	18.81	5.71	18.01	4.98	18.31	5.49	17.32	5.37	22.49	6.88	25.25	7.22	18.77	5.54	17.54	4.97
ud1	109.51	50.05	102.91	46.58	103.02	49.04	92.77	43.59	130.58	55.78	149.08	66.61	100.26	46.69	105.77	47.45
unrf1	80.93	9.95	77.56	8.99	82.08	10.11	75.42	10.01	91.41	11.14	107.8	11.44	83.1	9.54	75.81	7.17
**SCA**																
Instance	COM		COM_LOG		COM_PIECE		COM_SINE		COM_SINGER		COM_SINU		COM_TENT		COM_CIRCLE	
	avg	std	avg	std	avg	std	avg	std	avg	std	avg	std	avg	std	avg	std
u41	57.85	31.92	53.27	32.56	48.24	30.82	54.85	31.88	50.83	33.42	53.82	32.23	55.09	33.89	52.69	33.76
u51	94.93	44.7	89.42	47.43	88.39	46.21	92.14	46.11	91.28	44.58	91.65	49.98	88.78	48.35	87.94	45.74
u61	42.75	25.91	40.17	25.31	40.12	25.79	39.13	24.18	38.98	23.42	41.74	24.52	38.98	23.26	40.78	22.71
uclr10	3.5	1.04	3.54	1.2	3.47	1.11	3.52	1.19	3.62	1.16	3.87	1.31	3.49	1.09	3.34	0.96
uclr11	7.94	2.17	7.88	2.27	7.96	2.29	7.79	2.23	8.27	2.37	8.36	2.25	7.77	2.09	7.85	2.22
uclr12	104.76	57.47	86.05	44.98	85.84	51.69	87.05	48.32	106.66	52.92	104.2	55.57	85.06	52.11	86.3	46.68
uclr13	275.96	111.41	280.33	122.21	261.69	159.87	259.02	114.09	284.46	144.76	316.94	159.96	276.33	134.25	241.48	131.9
ucyc06	3.77	1.24	3.83	1.45	3.74	1.31	3.57	1.07	4.04	1.53	3.81	1.28	3.61	1.14	3.86	1.39
ucyc07	24.82	6.5	24.92	7.21	25.11	7.17	25.0	7.88	25.33	6.46	26.77	7.71	24.28	5.87	24.58	7.32
ud1	144.89	65.01	145.73	70.75	137.93	67.76	134.53	64.12	132.91	65.57	144.5	72.18	138.86	64.85	142.76	65.8
unrf1	99.6	17.16	106.35	14.83	108.03	12.31	106.42	12.29	111.57	23.84	111.0	16.53	111.44	18.45	110.93	18.82
**WOA**																
Instance	COM		COM_LOG		COM_PIECE		COM_SINE		COM_SINGER		COM_SINU		COM_TENT		COM_CIRCLE	
	avg	std	avg	std	avg	std	avg	std	avg	std	avg	std	avg	std	avg	std
u41	50.11	31.85	50.44	29.5	46.88	29.49	47.91	29.97	48.83	30.76	54.5	31.14	47.29	31.38	53.67	31.62
u51	83.85	43.93	84.01	44.47	81.34	43.08	80.58	44.09	81.87	45.84	87.69	46.78	82.64	42.92	79.37	47.88
u61	37.09	23.71	38.88	20.3	36.24	23.41	32.45	20.51	33.79	20.67	37.36	23.84	36.66	25.16	38.38	23.03
uclr10	2.96	0.97	3.09	1.03	2.89	0.84	3.02	1.01	2.95	0.86	3.31	1.14	2.81	0.82	2.85	0.88
uclr11	6.63	1.84	6.73	1.92	6.5	1.73	6.72	1.9	6.71	1.77	7.15	1.96	6.54	1.78	6.27	1.52
uclr12	85.94	46.45	78.45	50.1	71.84	45.83	68.78	37.83	76.43	45.27	83.12	46.84	80.58	43.39	67.8	40.58
uclr13	222.59	115.75	233.53	99.63	222.18	97.32	229.41	98.89	230.9	115.19	235.46	133.72	201.69	99.54	196.28	93.73
ucyc06	3.47	1.29	3.32	1.22	3.42	1.21	3.17	0.93	3.53	1.32	3.69	1.26	3.32	1.17	3.33	1.15
ucyc07	21.46	6.17	21.64	6.8	20.94	5.7	21.45	6.6	21.68	5.49	24.98	8.0	20.7	5.52	21.11	6.43
ud1	116.53	60.45	117.64	65.23	120.59	67.39	113.73	64.46	120.53	59.93	147.95	62.93	120.33	62.66	120.68	55.17
unrf1	96.08	11.65	101.44	22.57	97.4	16.6	104.06	17.96	101.92	17.7	107.56	20.41	99.17	21.63	100.89	18.02

**Table A17:** Computational efficiency summary of the proposed chaotic binarization framework for the Unicost Set Covering Problem (USCP) instances. Columns report the mean execution time and standard deviation (std) in seconds, calculated over 31 independent runs for the GWO, SCA, and WOA metaheuristics under the Elitist (ELIT) rule across different chaotic map configurations.

**GWO**																
Instance	ELIT		ELIT_LOG		ELIT_PIECE		ELIT_SINE		ELIT_SINGER		ELIT_SINU		ELIT_TENT		ELIT_CIRCLE	
	avg	std	avg	std	avg	std	avg	std	avg	std	avg	std	avg	std	avg	std
u41	38.51	21.32	38.73	20.5	36.16	20.78	39.98	20.16	44.14	23.85	46.91	24.5	38.37	19.38	34.68	18.6
u51	61.14	32.7	67.22	34.07	61.16	32.66	67.6	32.46	78.17	39.46	80.55	38.95	66.37	31.83	62.35	29.92
u61	30.78	16.86	32.69	18.2	33.16	17.28	32.72	16.83	36.58	19.1	37.77	20.44	31.44	17.23	32.34	16.38
uclr10	2.21	0.64	2.52	0.66	2.22	0.66	2.38	0.76	3.52	1.2	3.96	1.15	2.26	0.69	1.93	0.57
uclr11	5.4	1.55	5.7	1.52	5.31	1.5	5.36	1.22	7.39	2.08	9.24	2.62	5.06	1.17	4.54	1.26
uclr12	58.29	35.04	71.05	41.28	63.51	38.38	62.83	39.94	94.62	52.65	105.19	64.04	63.55	30.22	53.14	29.43
uclr13	192.76	89.64	195.71	107.48	221.89	96.78	201.21	91.84	323.14	146.87	313.44	174.51	186.62	98.62	140.94	79.33
ucyc06	1.94	0.62	2.18	0.73	1.94	0.59	2.12	0.75	2.72	0.88	3.41	1.29	1.89	0.56	1.63	0.54
ucyc07	11.02	3.05	13.0	4.0	11.1	3.13	11.95	3.72	17.5	5.21	21.17	6.47	11.17	3.14	8.4	2.33
ud1	87.98	43.99	107.7	43.04	89.77	39.92	90.56	44.67	117.46	57.43	133.58	59.85	86.76	40.51	81.36	38.58
unrf1	90.04	17.41	90.86	17.11	87.86	20.03	83.89	14.24	106.61	23.66	107.32	17.63	85.53	20.1	71.13	11.87
**SCA**																
Instance	ELIT		ELIT_LOG		ELIT_PIECE		ELIT_SINE		ELIT_SINGER		ELIT_SINU		ELIT_TENT		ELIT_CIRCLE	
	avg	std	avg	std	avg	std	avg	std	avg	std	avg	std	avg	std	avg	std
u41	37.52	24.76	38.74	24.0	37.99	22.19	37.39	22.68	45.29	25.38	46.81	26.36	35.8	24.06	34.48	20.87
u51	60.05	36.57	68.73	35.67	67.83	32.65	62.38	35.51	81.28	38.05	80.09	40.24	66.73	33.94	58.51	33.03
u61	31.07	20.74	30.1	17.98	33.7	19.76	32.48	19.87	37.35	21.67	37.36	22.02	34.09	19.97	30.06	18.3
uclr10	2.4	0.76	2.6	0.84	2.32	0.68	2.46	0.85	3.01	0.88	3.39	1.14	2.25	0.61	2.19	0.74
uclr11	5.54	1.5	5.88	1.65	5.42	1.31	5.7	1.62	6.87	1.8	7.6	2.05	5.67	1.47	4.6	1.13
uclr12	63.15	37.09	60.8	36.13	63.45	38.45	57.08	34.33	81.95	47.36	86.13	50.35	58.17	33.37	50.47	31.54
uclr13	171.46	87.0	201.9	104.92	183.18	90.62	171.16	77.74	247.6	137.26	244.75	132.17	184.14	91.82	146.03	75.02
ucyc06	1.96	0.58	2.14	0.68	1.91	0.59	2.13	0.71	2.76	0.97	3.1	1.13	1.9	0.55	1.72	0.63
ucyc07	11.8	3.62	13.06	3.82	12.14	3.55	11.82	3.29	17.54	5.53	18.96	5.13	11.81	3.72	9.02	2.57
ud1	92.04	45.08	87.27	48.76	103.92	51.91	96.4	48.94	115.66	57.93	122.78	60.38	94.03	46.76	85.68	41.91
unrf1	75.7	11.93	79.51	7.23	76.2	6.98	78.35	6.2	90.81	12.15	97.57	8.84	76.26	7.52	71.96	5.95
**WOA**																
Instance	ELIT		ELIT_LOG		ELIT_PIECE		ELIT_SINE		ELIT_SINGER		ELIT_SINU		ELIT_TENT		ELIT_CIRCLE	
	avg	std	avg	std	avg	std	avg	std	avg	std	avg	std	avg	std	avg	std
u41	31.61	19.76	33.44	19.05	31.78	19.59	35.05	21.47	40.02	24.14	40.48	23.38	32.22	19.35	26.33	16.69
u51	53.75	29.55	60.09	32.65	58.25	28.92	61.72	32.39	63.45	37.79	69.33	37.73	60.14	32.81	45.94	26.16
u61	28.6	17.38	29.87	17.98	27.14	17.15	29.71	16.99	31.52	20.33	31.59	19.87	27.74	16.06	24.6	13.3
uclr10	1.83	0.51	2.19	0.72	1.82	0.53	2.0	0.6	2.51	0.83	2.49	0.77	1.89	0.6	2.12	1.21
uclr11	4.23	0.96	5.04	1.44	4.2	1.03	4.71	1.28	5.52	1.42	5.87	1.74	4.32	1.16	3.71	1.14
uclr12	41.11	26.94	53.02	30.31	42.55	25.82	52.78	28.4	62.74	35.14	63.47	36.7	44.31	27.51	33.15	19.21
uclr13	109.72	67.92	142.37	75.33	113.77	65.48	146.97	77.15	171.51	87.04	179.94	99.94	117.68	67.68	108.14	61.93
ucyc06	1.55	0.41	1.78	0.55	1.58	0.49	1.7	0.5	2.3	0.81	2.32	0.8	1.64	0.59	1.35	0.46
ucyc07	8.55	1.91	10.34	2.8	8.91	2.77	9.85	2.77	14.12	4.46	14.12	4.04	9.01	2.71	6.57	1.91
ud1	79.21	40.49	83.16	42.85	75.11	42.07	79.56	41.13	101.29	51.85	95.3	53.81	76.46	41.44	62.91	32.76
unrf1	67.07	7.08	72.21	11.42	64.78	7.12	67.6	7.57	79.73	8.91	78.41	8.78	65.74	4.79	65.27	19.2

**Table A18:** Computational efficiency summary of the proposed chaotic binarization framework for the Unicost Set Covering Problem (USCP) instances. Columns report the mean execution time and standard deviation (std) in seconds, calculated over 31 independent runs for the GWO, SCA, and WOA metaheuristics under the Standard (STD) rule across different chaotic map configurations.

**GWO**																
Instance	STD		STD_LOG		STD_PIECE		STD_SINE		STD_SINGER		STD_SINU		STD_TENT		STD_CIRCLE	
	avg	std	avg	std	avg	std	avg	std	avg	std	avg	std	avg	std	avg	std
u41	43.85	23.34	31.67	18.85	34.1	18.99	31.76	16.15	42.08	20.89	43.35	24.59	36.02	17.85	32.55	17.04
u51	76.25	34.33	56.54	29.46	62.19	29.5	51.24	27.67	74.05	34.33	79.03	39.09	56.68	30.41	56.68	27.13
u61	42.04	20.55	29.5	15.49	27.04	16.16	28.29	14.8	35.5	18.28	35.92	20.21	29.02	15.87	29.95	14.99
uclr10	2.1	0.54	1.83	0.54	1.86	0.49	1.69	0.48	2.64	0.81	3.51	1.06	1.96	0.61	1.82	0.53
uclr11	4.72	1.33	3.84	0.95	4.2	1.0	3.67	0.95	5.92	1.66	7.91	1.92	4.22	1.0	3.66	0.99
uclr12	39.15	23.07	33.19	18.81	41.81	21.58	32.99	18.73	63.69	37.46	95.79	55.31	34.26	21.04	34.49	17.24
uclr13	106.74	59.49	91.76	49.14	104.63	56.9	78.83	46.01	173.52	98.01	282.4	124.0	102.3	43.33	97.96	39.02
ucyc06	1.99	0.63	1.73	0.54	1.77	0.59	1.6	0.51	2.65	0.9	3.18	1.1	1.76	0.53	1.49	0.49
ucyc07	10.39	3.29	9.51	2.67	9.57	2.77	8.13	2.36	16.43	5.25	19.88	5.78	9.97	3.02	7.2	2.27
ud1	96.75	40.77	69.97	33.56	75.42	33.61	68.73	30.21	107.84	49.64	114.61	58.03	73.38	37.07	60.9	26.04
unrf1	74.67	7.74	60.64	8.23	63.3	8.02	54.88	6.81	81.46	12.93	92.25	13.19	65.47	10.45	55.45	7.32
**SCA**																
Instance	STD		STD_LOG		STD_PIECE		STD_SINE		STD_SINGER		STD_SINU		STD_TENT		STD_CIRCLE	
	avg	std	avg	std	avg	std	avg	std	avg	std	avg	std	avg	std	avg	std
u41	41.54	21.17	34.3	22.5	33.73	19.83	33.52	21.05	39.33	24.05	42.51	25.42	34.28	20.21	30.34	18.9
u51	78.89	35.08	61.62	30.94	63.29	31.38	55.74	31.11	67.99	34.04	70.24	38.34	61.33	31.59	56.86	29.35
u61	32.92	14.91	28.33	17.33	28.94	17.63	30.34	17.62	29.14	19.53	35.73	19.37	30.1	16.92	28.75	17.01
uclr10	3.33	1.86	1.93	0.53	1.99	0.66	1.85	0.5	2.52	0.87	2.53	0.72	2.01	0.66	1.72	0.47
uclr11	6.95	3.84	4.52	1.13	4.36	1.21	4.26	1.03	5.52	1.49	5.93	1.57	4.34	1.08	3.9	0.98
uclr12	46.0	24.76	42.45	23.83	39.16	26.55	41.59	25.86	62.88	35.58	58.88	35.32	44.28	23.12	39.69	23.2
uclr13	119.48	53.55	133.46	67.41	116.38	60.31	121.35	56.86	161.83	77.84	180.82	88.95	113.05	61.86	98.99	52.3
ucyc06	2.06	0.64	1.99	0.72	1.72	0.47	1.84	0.63	2.43	0.83	2.7	0.96	1.79	0.61	1.5	0.47
ucyc07	12.1	3.96	11.09	3.32	10.34	3.22	10.18	2.93	15.04	4.79	16.43	4.76	10.32	3.21	8.38	2.48
ud1	102.05	46.63	82.23	40.86	81.8	37.31	78.99	38.6	98.74	47.74	103.92	54.77	76.52	38.98	74.0	37.1
unrf1	72.68	11.9	64.63	9.29	62.87	6.8	62.67	6.74	73.39	9.13	78.68	8.8	63.2	6.2	62.36	6.46
**WOA**																
Instance	STD		STD_LOG		STD_PIECE		STD_SINE		STD_SINGER		STD_SINU		STD_TENT		STD_CIRCLE	
	avg	std	avg	std	avg	std	avg	std	avg	std	avg	std	avg	std	avg	std
u41	39.97	21.67	30.59	18.74	28.8	16.77	30.45	18.51	35.07	20.46	34.09	21.06	29.52	16.79	25.14	15.72
u51	66.59	33.22	55.99	28.5	49.59	28.8	49.46	28.51	61.67	34.72	57.04	35.56	51.33	26.31	48.22	26.41
u61	33.94	20.54	27.18	15.99	25.39	14.33	26.08	14.55	28.94	16.95	29.98	18.44	25.36	14.8	25.36	12.52
uclr10	1.82	0.58	1.69	0.52	1.5	0.49	1.56	0.48	2.1	0.73	2.1	0.59	1.59	0.5	1.33	0.37
uclr11	3.71	1.06	3.64	0.93	3.39	0.92	3.43	0.93	4.45	1.21	4.97	1.39	3.36	0.93	2.91	0.72
uclr12	31.29	19.45	35.76	20.29	30.84	15.18	33.69	19.64	50.92	24.25	62.62	32.58	30.04	16.73	28.42	15.86
uclr13	89.82	45.51	87.67	45.71	94.38	46.03	82.89	49.47	133.62	64.76	160.08	84.0	79.66	43.44	66.62	33.52
ucyc06	1.61	0.52	1.61	0.55	1.37	0.45	1.54	0.55	1.97	0.66	2.2	0.83	1.43	0.46	1.15	0.36
ucyc07	8.25	2.6	9.11	2.77	7.39	2.1	8.0	2.25	12.35	3.74	12.88	3.64	7.58	2.04	5.69	1.53
ud1	80.44	44.67	68.18	31.46	62.08	29.34	62.9	34.33	83.07	41.44	88.65	43.42	63.26	30.38	57.7	29.62
unrf1	57.49	7.89	50.68	4.94	49.09	5.49	49.24	7.29	59.6	9.55	64.71	10.21	49.02	7.69	47.74	7.16
